# Metagenomic evaluation of peanut rhizosphere microbiome from the farms of Saurashtra regions of Gujarat, India

**DOI:** 10.1038/s41598-024-61343-5

**Published:** 2024-05-08

**Authors:** Krunal R. Hirpara, Ankit T. Hinsu, Ramesh K. Kothari

**Affiliations:** 1 Department of Biosciences, Saurashtra University, Rajkot, Gujarat 360005 India; 2 Present Address: Royal Veterinary College, London, AL9 7TA UK

**Keywords:** Next-generation sequencing, Soil microbiology, Microbial communities

## Abstract

The narrow zone of soil around the plant roots with maximum microbial activity termed as rhizosphere. Rhizospheric bacteria promote the plant growth directly or indirectly by providing the nutrients and producing antimicrobial compounds. In this study, the rhizospheric microbiota of peanut plants was characterized from different farms using an Illumina-based partial 16S rRNA gene sequencing to evaluate microbial diversity and identify the core microbiome through culture-independent (CI) approach. Further, all rhizospheric bacteria that could grow on various nutrient media were identified, and the diversity of those microbes through culture-dependent method (CD) was then directly compared with their CI counterparts. The microbial population profiles showed a significant correlation with organic carbon and concentration of phosphate, manganese, and potassium in the rhizospheric soil. Genera like *Sphingomicrobium, Actinoplanes, Aureimonas _A, Chryseobacterium,* members from *Sphingomonadaceae*, *Burkholderiaceae*, *Pseudomonadaceae*, *Enterobacteriaceae* family, and Bacilli class were found in the core microbiome of peanut plants. As expected, the current study demonstrated more bacterial diversity in the CI method. However, a higher number of sequence variants were exclusively present in the CD approach compared to the number of sequence variants shared between both approaches. These CD-exclusive variants belonged to organisms that are more typically found in soil. Overall, this study portrayed the changes in the rhizospheric microbiota of peanuts in different rhizospheric soil and environmental conditions and gave an idea about core microbiome of peanut plant and comparative bacterial diversity identified through both approaches.

## Introduction

Plant-associated microbes can be differentiated into different types based on their locations and vicinity to plants^1^. The rhizosphere is home to a diverse microbial population that engages in microbe–microbe and microbe–plant communication via plant exudates^2,3^. Rhizospheric bacteria respond to plant exudates and assist plants in various ways, including nutrient uptake, stress tolerance, disease resistance, and by participating in major biogeochemical processes^2,4^. Although the value of the rhizospheric microbiome for plant growth has long been understood, very little is known about the vast majority of rhizospheric bacteria. To enhance plant growth and health, it is crucial to comprehend who the individuals in the rhizosphere microbiome are and what they are accomplishing^4^. Understanding the rhizospheric microbiome is essential for the growth of agriculturally important crops like peanuts in a sustainable manner.

On the other hand, the rhizospheric microbiome is highly dynamic and changes in response to various internal and external conditions, making it an incredibly complex ecosystem^1,5^. However**,** the challenging aspect is isolating and characterizing these bacteria, as most microorganisms are not yet culturable for various reasons. As a result, traditional microbiological techniques, also known as culture-dependent approaches (CD), are unable to provide a complete picture of bacterial diversity. Conversely, sequence-driven metagenomics (also called as culture-independent, CI) has emerged as the approach of choice to study microbiota from various habitats.

Many prior researchers have used 16S rRNA gene-based community profiling to analyze rhizospheric microbiota from a variety of plant and crop species, including Arabidopsis, rice, millet, soybean, corn, barley, wheat, tomato, grapes, and many more^1,2,6–11^. While numerous studies have been conducted to investigate the impacts of soil type, geographic location, crop developmental cycle, crop genotypes, and a variety of other variables^1,2,5,7,10,12–26^. Limited studies have been conducted for bacterial community profiling on the peanut rhizosphere through metagenomic approach under natural environmental conditions^27–29^. However, many studies have shown that many species miss out on even the metagenomic technique^30^. However, some of these overlooked species thrive well in adequate environments, showing that metagenomics overlooks certain commonly occurring organisms^31,32^. To overcome the limitation arising from both approaches, in this study, the peanut rhizospheric microbiome was evaluated and characterized through both approaches. For metagenomic study, 16S rRNA gene amplicon-based approach was used to study the microbiota and discover the core microbiome of peanut rhizosphere. Moreover, samples were also studied through the CD approach and compared with the CI approach to get a complete idea of bacterial diversity. To do that, the microorganisms were identified by next-generation sequencing (NGS) after being cultivated on eight distinct media that were appropriate for various microbe kinds.

Unlike most previous studies comparing culture-dependent versus culture-independent techniques, this study used NGS to sequence the partial 16S rRNA gene of all the colonies present on the plate rather than Sanger sequencing to sequence individual colonies^32–34^. So, the comparative analysis was done to evaluate the presence-absence of organisms as observed by both approaches from the peanut rhizosphere. For that, all rhizospheric soil samples were collected from 5 districts of Gujarat (INDIA) covering approximately 25,000 km^2^.

To create effective bio-strategies, such as bio-fertilizers, to boost crop output, the study's goal was to identify the overall bacterial diversity using both approaches and further discover the core microbiome with reported potential plant growth-promoting genera in the peanut rhizosphere.

## Results

In the present study, the structural diversities of bacterial community were analyzed from the rhizospheric soils obtained from agricultural farms (Fig. S1, Table 1). The analysis of physico-chemical properties of the rhizospheric soils indicated that there were significant differences (Kruskal–Wallis p-value < 0.05) in pH and electrical conductivity (EC) as well as concentrations of all measured macro and micronutrients, except for phosphate and sulfur among all the farms (Fig. S2). While the Principal Component Analysis (PCA) of rhizospheric soil property shows the distribution of all samples based on the farms (Fig. S3).

**Table 1:** Details about the samples collection and sample code used during analysis.

Sr. no.	Collection date	Sample codes		District	Location/Village
		Culture independent approach	Culture dependent approach		
1	14-08-2019	F01(1-5)	F01	Rajkot	Jamvadi
2	17-08-2019	F02(1-5)	F02	Porbandar	Vada
3	17-08-2019	F03(1-5)	F03	Junagadh	Galvav
4	17-08-2019	F04(1-5)	F04	Porbandar	Ranakandorna
5	19-08-2019	F05(1-5)	F05	Amreli	Rajula
6	19-08-2019	F07(1-5)	F07	Amreli	Piperllag
7	21-08-2019	F08(1-5)	F08	Gir Somnath	Mitiyaj
8	21-08-2019	F10(1-5)	F10	Gir Somnath	Khorasa Gir
9	21-08-2019	F11(1-5)	F11	Junagadh	Keshod
10	23-08-2019	F12(1-5)	F12	Junagadh	Nava gam
11	23-08-2019	F13(1-5)	F13	Rajkot	Fareni
12	23-08-2019	F14(1-5)	F14	Rajkot	Sidsar
13	23-08-2019	F15(1-5)	F15	Amreli	Utvad

### Characterization of rhizospheric microbiota through metagenomic study

#### Culture-independent diversity

In the CI approach run, around 7.5 million sequence reads were generated from 65 samples, with an average count of 116,687 reads per sample. The DADA2 pipeline inferred 17,719 Amplicon Sequence Variants (ASVs) from 4.8 million reads (64.33%). After filtering ASVs, 8,042 ASVs from 59 samples remained which were further analyzed (detailed information in supplementary method). All ASVs were taxonomically classified as bacteria, further belonging to 31 phyla, 67 classes, 177 orders, 315 families, 665 genera and 87 species. The reads distribution across taxonomic levels was highlighted in Table S1.

**Figure 1:**
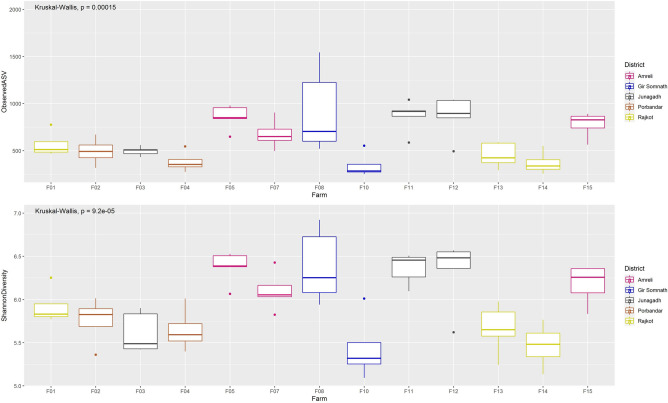
Plot highlighting each farm’s alpha diversity. Alpha diversity measures Observed ASVs (top), and Shannon Index (bottom) plotted for each farm. p-value from Kruskal–Wallis test comparing all farms is mentioned on the top.

The number of observed ASVs ranged from 252 (F10(4)) to 1543 (F08(1)) (Fig. 1). The Shannon diversity index, which accounts for the relative proportion of each ASV, was observed in the range of 5.09 (F-10(4)) to 6.92 (F-08(1)) (Fig. 1). There were significant differences in the number of observed ASVs (Kruskal–Wallis p-value = 0.00015) and Shannon index (Kruskal–Wallis p-value = 0.000092) among all farms.

#### Taxonomic content of microbial communities

**Figure 2:**
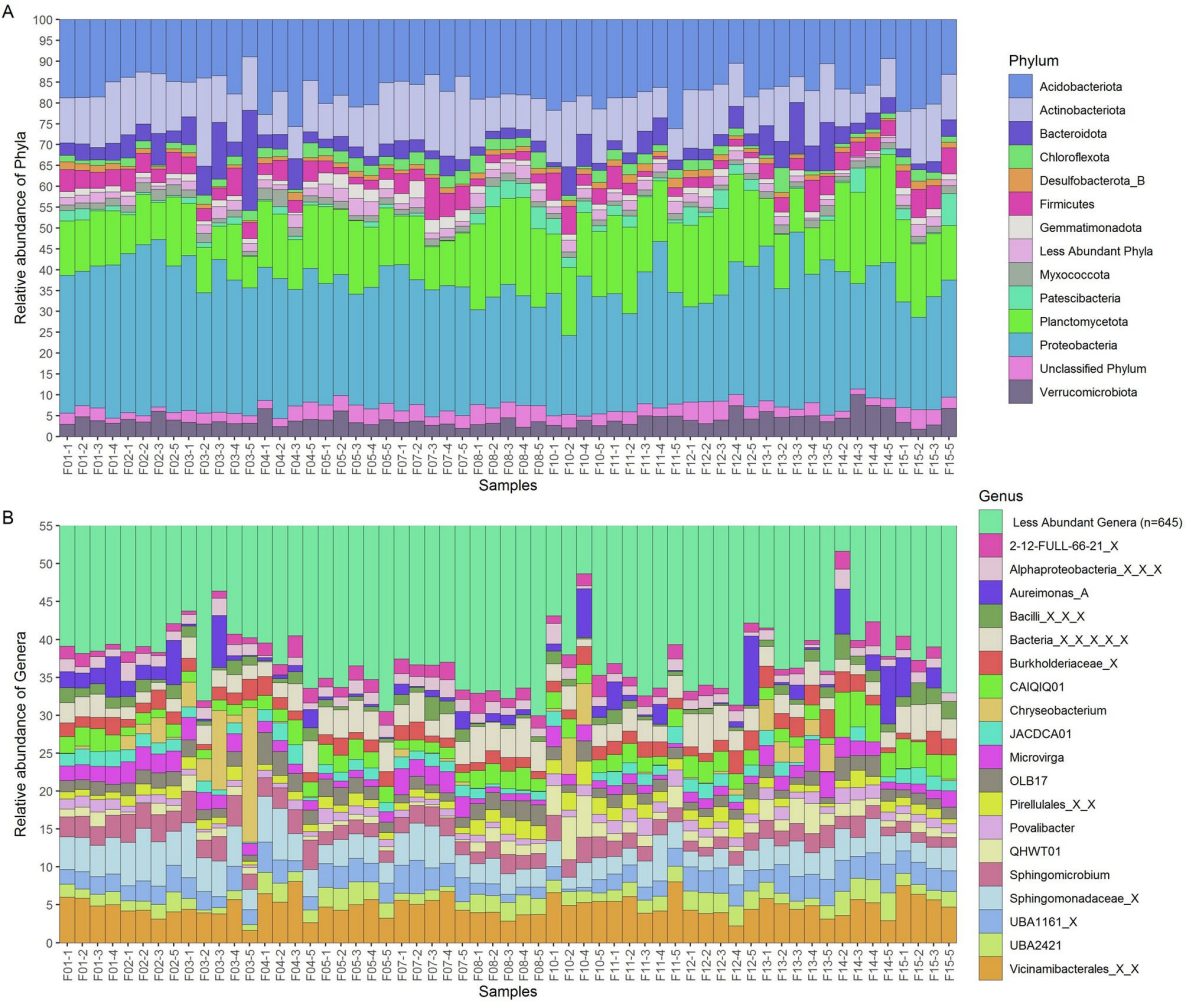
Taxonomic distribution of samples. Taxonomic distribution at (**A**) phylum level and (**B**) Genus level. Only the most abundant taxa are plotted for both levels.

Phyla like Acidobacteriota, Actinobacteriota, Planctomycetota and Proteobacteria were found most abundant in all rhizospheric soil samples (mean relative abundance > 5%) (Fig. 2A). At the phylum level, 17 out of 20 phyla (with mean relative abundance > 0.0001) showed significant differences (Kruskal–Wallis, BH p-value 0.05) among all farms (Table S2, Fig. S4). Genera like *Sphingomicrobium, CAIQIQ01*, *Povalibacter*, *UBA2421*, *QHWT01,* unknown members of *Sphingomonadaceae*, *UBA1161* family*,* and Vicinamibacterales order*,* and genus of unknown bacterium were the most abundant in all samples (mean relative abundance > 1%) (Fig. 2B, Fig. S5). Similarly, at genus level, 315 out of 647 (mean relative abundance > 0.00001) differed significantly (Kruskal–Wallis, BH p-value < 0.05) among different farms (Table S3). Very few highly abundant ASVs were assigned up to species level, including *Sphingomicrobium sp003097155*, *Microvirga lupini_A*, *P52-10 sp000516555*, *Ectobacillus funiculus* (Fig. S6). Other species observed in higher abundance include *Microvirga makkahensis*, *Pseudoduganella eburnean*, *Planctomyces_A sp001610835*, *Pseudomonas_M indica*, *Bacillus_BD endozanthoxylicus*, *Mycoplana sp900469965*, and *Metabacillus sp002871465*.

#### Changes among rhizospheric samples

**Figure 3:**
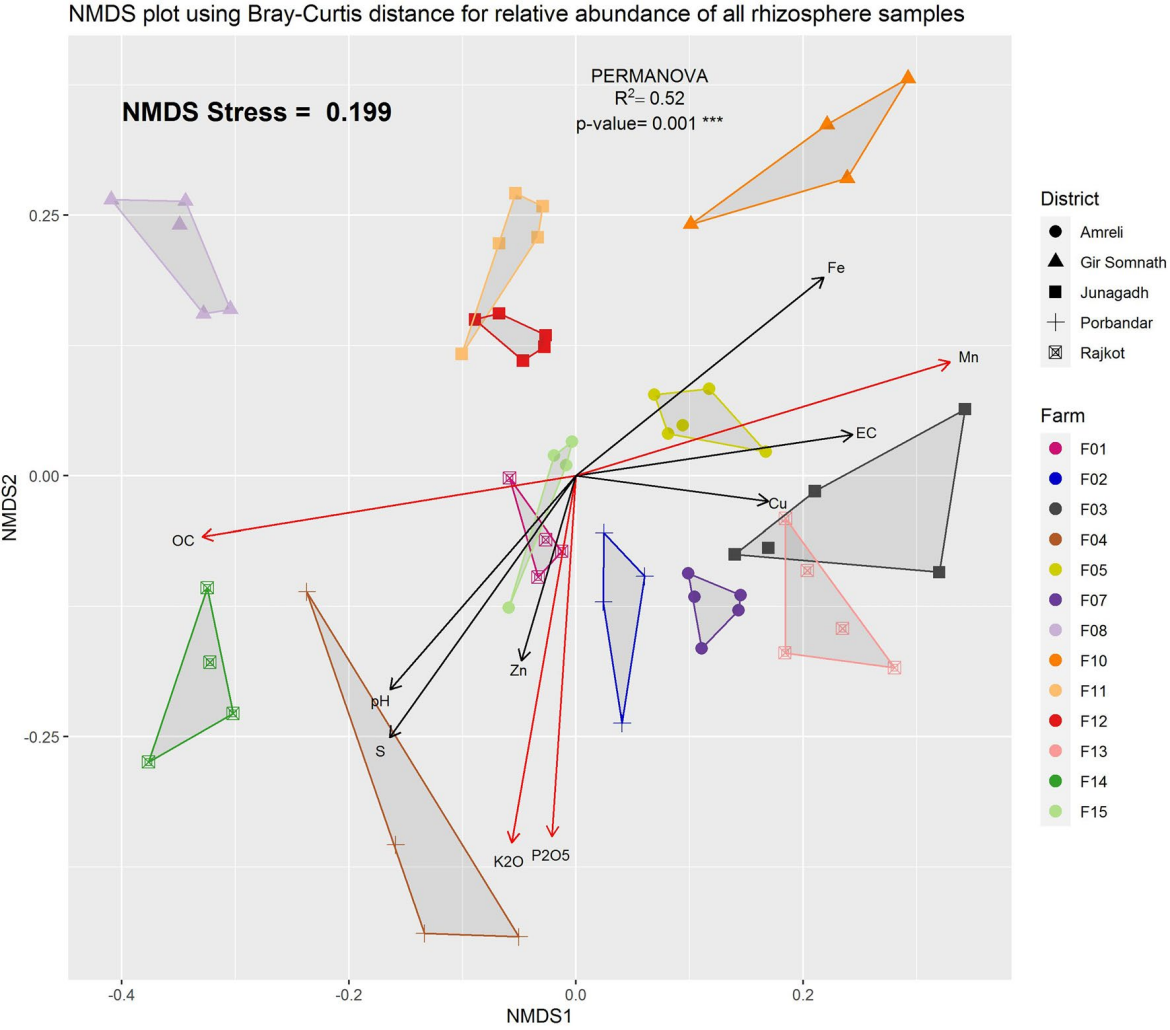
NMDS plot of Bray–Curtis distance calculated from all Rhizosphere samples. District of each farm is used as shape to denote the sample. Arrows are environment fit vectors that represent physical factors and nutritional concentrations. Vectors with significant associations are shown in red coloured arrow.

The changes in the rhizospheric samples were evaluated with respect to their geographic location. NMDS ordination on Bray–Curtis distance revealed that there were separate and distinct clusters of samples as per their geographical location. For example, samples of Rajkot, Amreli and Porbandar district farms formed clusters near to each other, while Gir-Somnath district samples formed separate clusters (Fig. 3). All the samples of Junagadh district were grouped near to Gir-Somnath district samples except F03 farm samples. The difference among geographic locations was further confirmed through PERMANOVA, where a significant difference was observed (p-value < 0.001). A pairwise-adonis between all pairs of farms was significantly different (Table S4). An environmental fit of all variables also revealed a significant association of organic carbon (OC), concentrations of potassium (K_2_O), phosphate (P_2_O_5_) and manganese (Mn) (Figs. 3, S7).

#### Core rhizosphere microbiome

**Figure 4:**
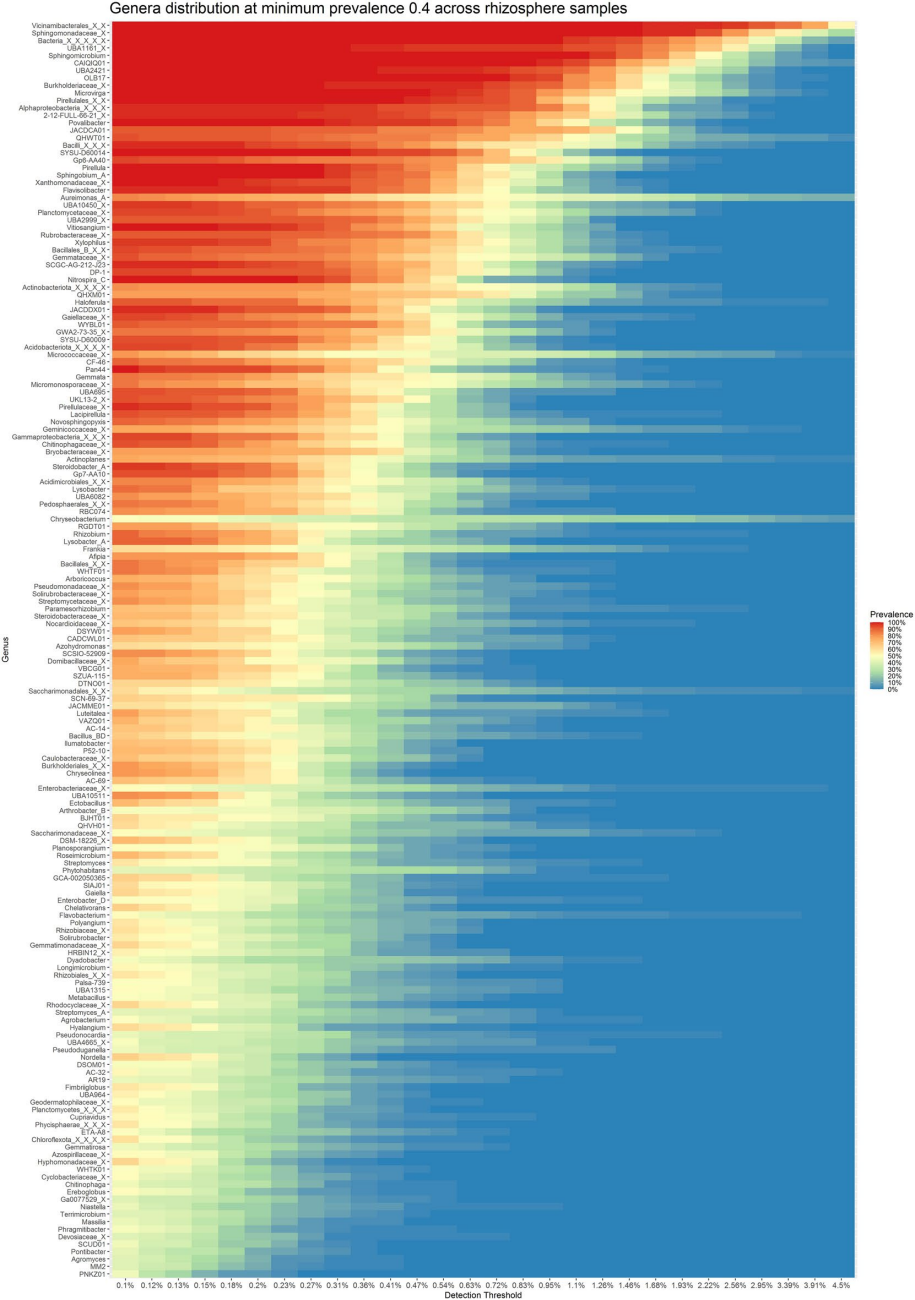
Plot representing core microbiome from rhizosphere samples. The graphic compares genus occurrence in samples with varied degrees of abundance. Only the genera with minimum prevalence of 0.4 at 0.001 abundance are plotted.

**Figure 5:**
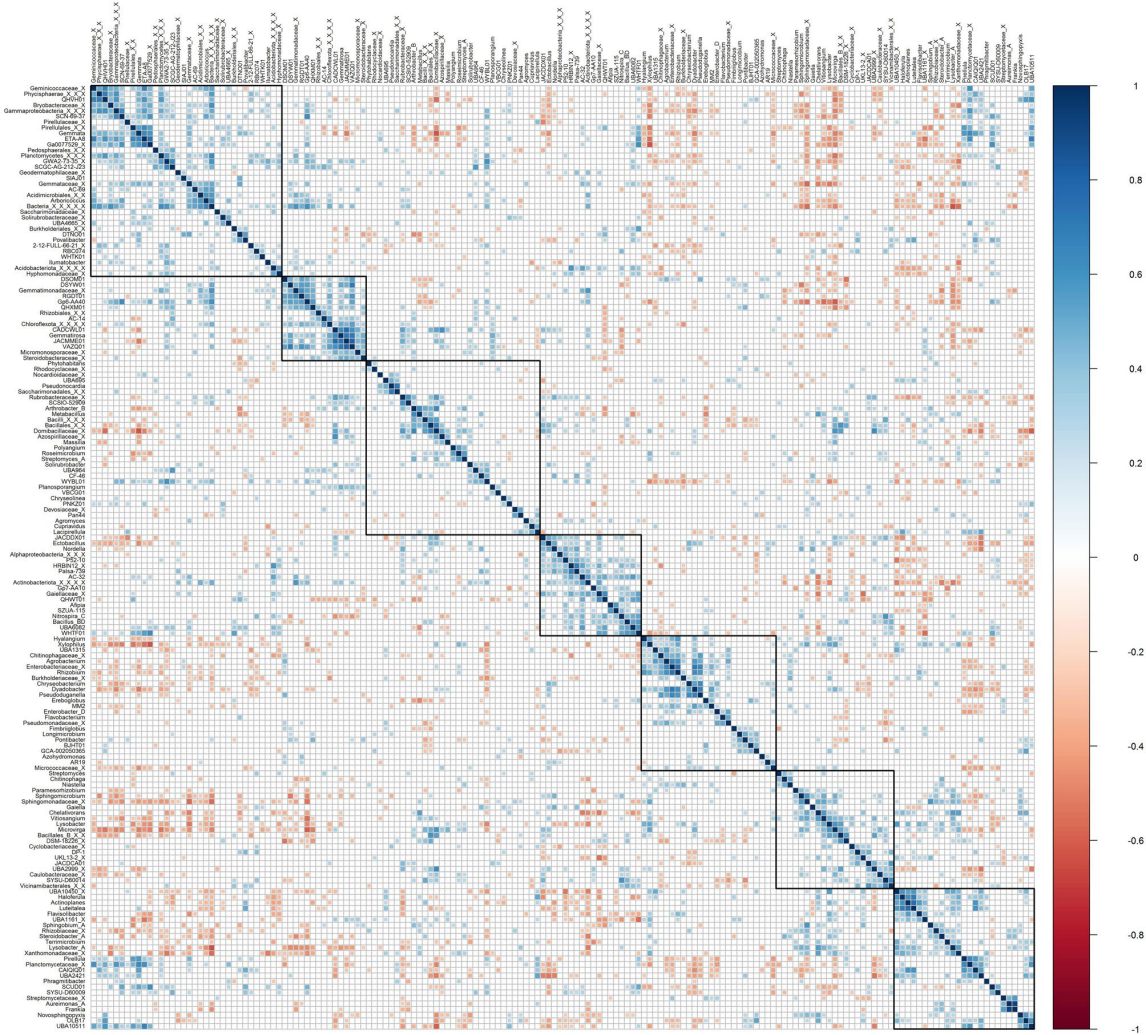
Correlation plot among genera from core microbiome. Only the significant (p-value < 0.05) correlations are plotted.

Total 168 genera were identified as part of the core microbiome of rhizosphere samples with a minimum abundance of 0.1% across 40% of samples (Fig. 4). To observe the pattern of co-occurrence, these taxa were further correlated with each other. Seven different clusters of genera could be made out from the significantly (p-value < 0.05) correlating genera (Fig. 5). It was discovered that three clusters were negatively correlated with all the other genera. These contained genera like *Sphingomicrobium, Chelativorans, Vitiosangium, Lysobacter, Microvirga*, *Dyadobacter*, unknown members of *Sphingomonadaceae*, *Rhizobiaceae*, *Xanthomonadaceae* and *Enterobacteriaceae* family. A separate cluster could also be made out containing mostly Bacilli class members like *Bacillus_BD*, *Ectobacillus*, *Domibacillus*, *Metabacillus* and unknown members of *Domibacillaceae* family, Bacillales_B, Bacillales order and Bacilli class. Many of these genera, particularly the more prevalent ones, exhibited a negative association with all other genera, which might explain their growth during the nodulation phases.

### Comparative analysis using both approaches

#### Comparative approach of CD and CI

In addition to above mentioned data, around 1,282,054 paired-end reads were generated for samples of CD approach run. DADA2-based pipeline generated 18,765 ASVs using sequences of both approaches, of which 6970 ASVs having more than thirty supporting reads were considered for further comparative analysis of CD and CI approaches (Detailed information in supplementary method).

#### Alpha diversity and taxonomic profile

**Figure 6:**
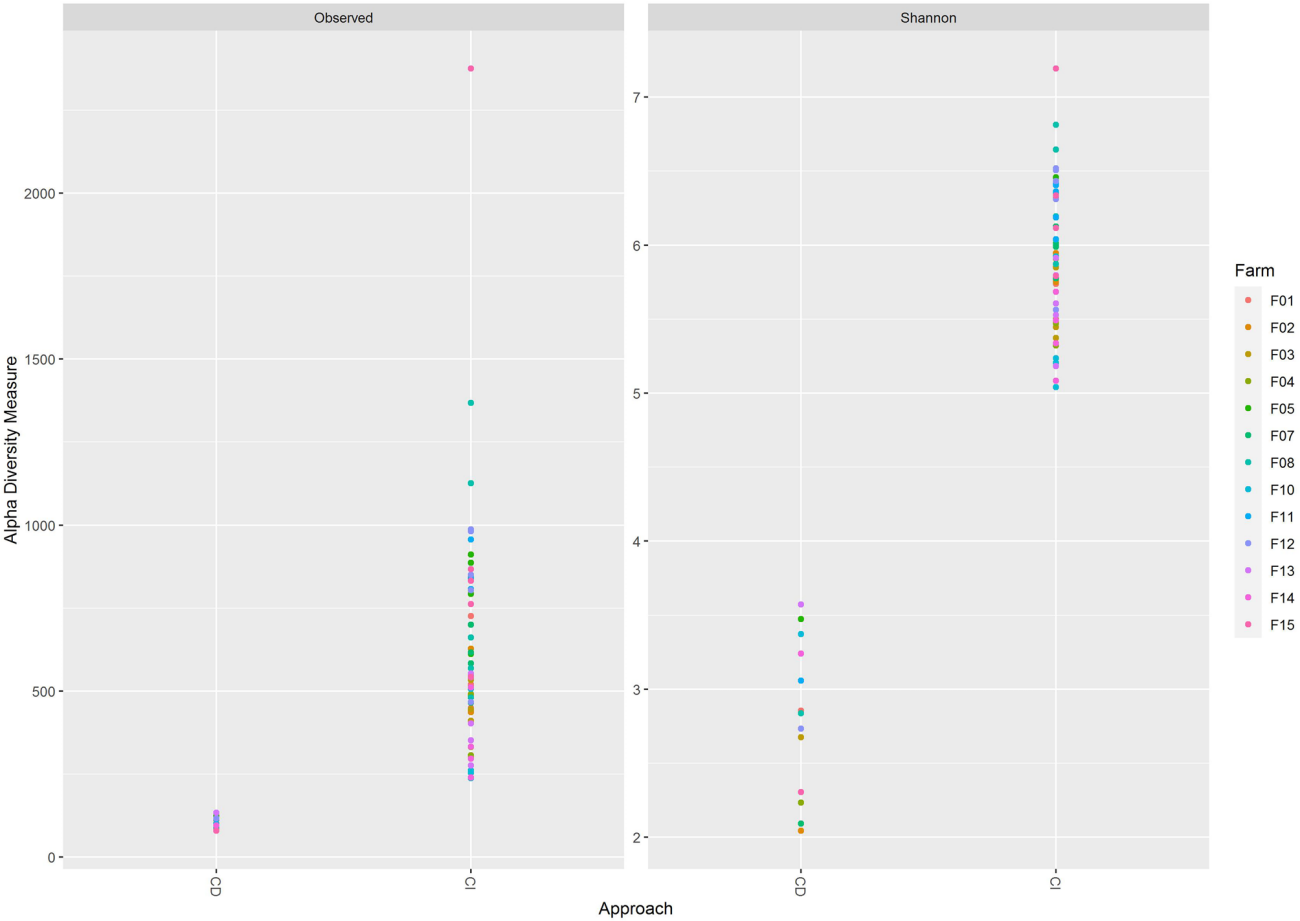
Observed ASV count and Shannon diversity distribution plot. The farms are coloured differentially. X-axis represents either CI approach or CD approach.

As expected, the culture-independent approach showed a higher number of ASVs (Wilcoxon test, BH adjusted p-value = 0.000000019) as well as higher Shannon Index (Wilcoxon test, BH adjusted p-value = 0.00000019) compared to cultured samples (Fig. 6).

**Figure 7:**
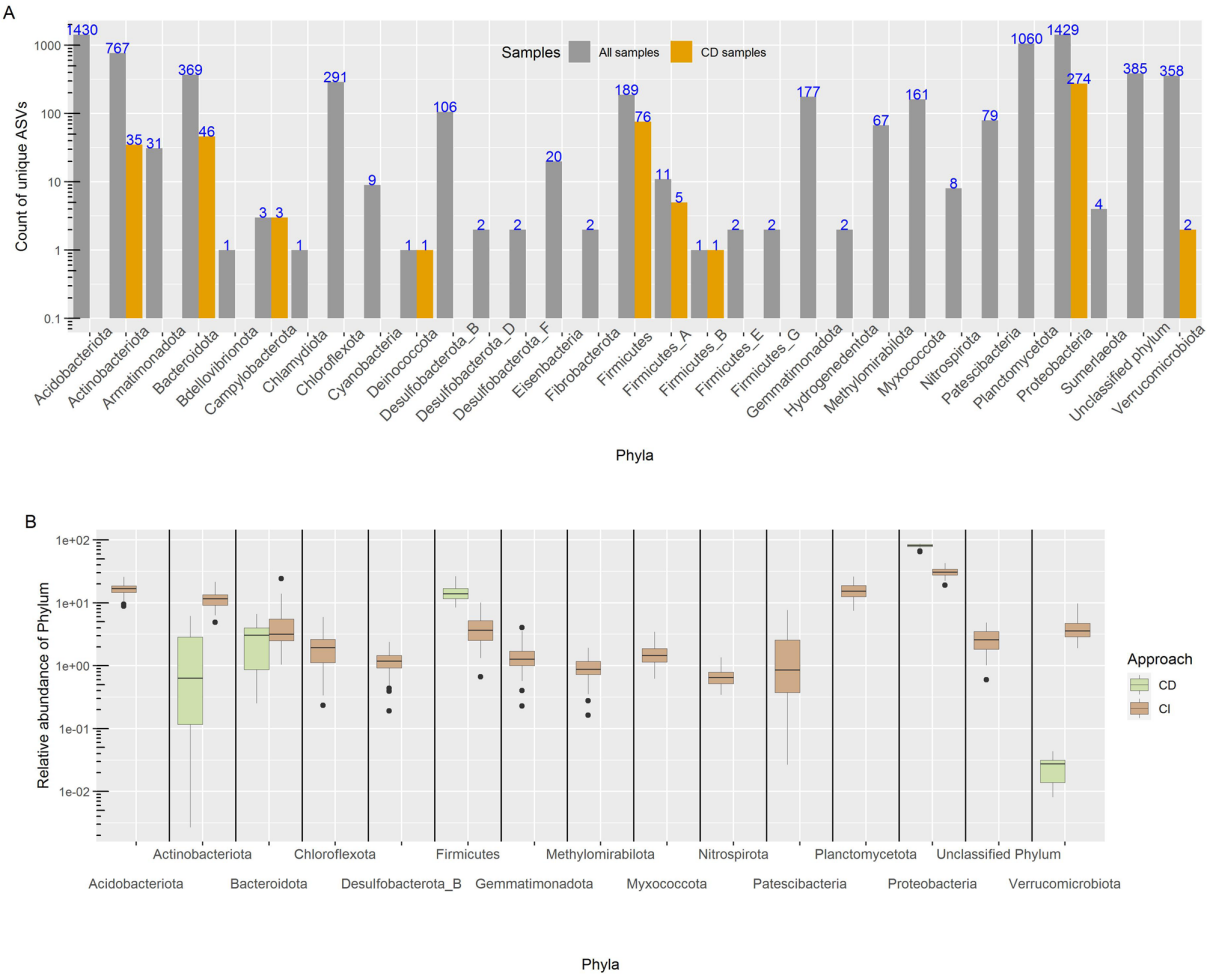
Plots representing count of unique ASVs and relative abundance of phyla. (**A**) ASVs detected across all phyla in all samples (including CD and CI samples, grey colour) and exclusively in culture-dependent samples (orange colour). (**B**) Relative abundance of top 15 phyla.

All ASVs were classified as Bacteria. However, 385 ASVs remained unassigned at the phylum level. From 31 detected phyla in CI samples, ASVs from only 9 phyla namely, Proteobacteria (274), Firmicutes (76), Bacteroidetes (46), Actinobacteria (35), Firmicutes_A (5), Campylobacterota (3), Verrucomicrobia (2), Deinococcus and Firmicutes_B (1) were detected in CD samples (Fig. 7A). Further, in the CI approach, the greatest number of ASVs belonged to Acidobacteria (1430) followed by Proteobacteria (1429) and Planctomycetes (1060) phyla. Proteobacteria was the dominating phyla in both approaches, with an average proportion in the CI approach of 34.50%, while the CD approach had abundance in the range of 64% to 87% (average value ~ 80%) (Fig. 7). The Proteobacteria phylum alone accounted for the majority of the proportion in CD approach samples.

**Figure 8:**
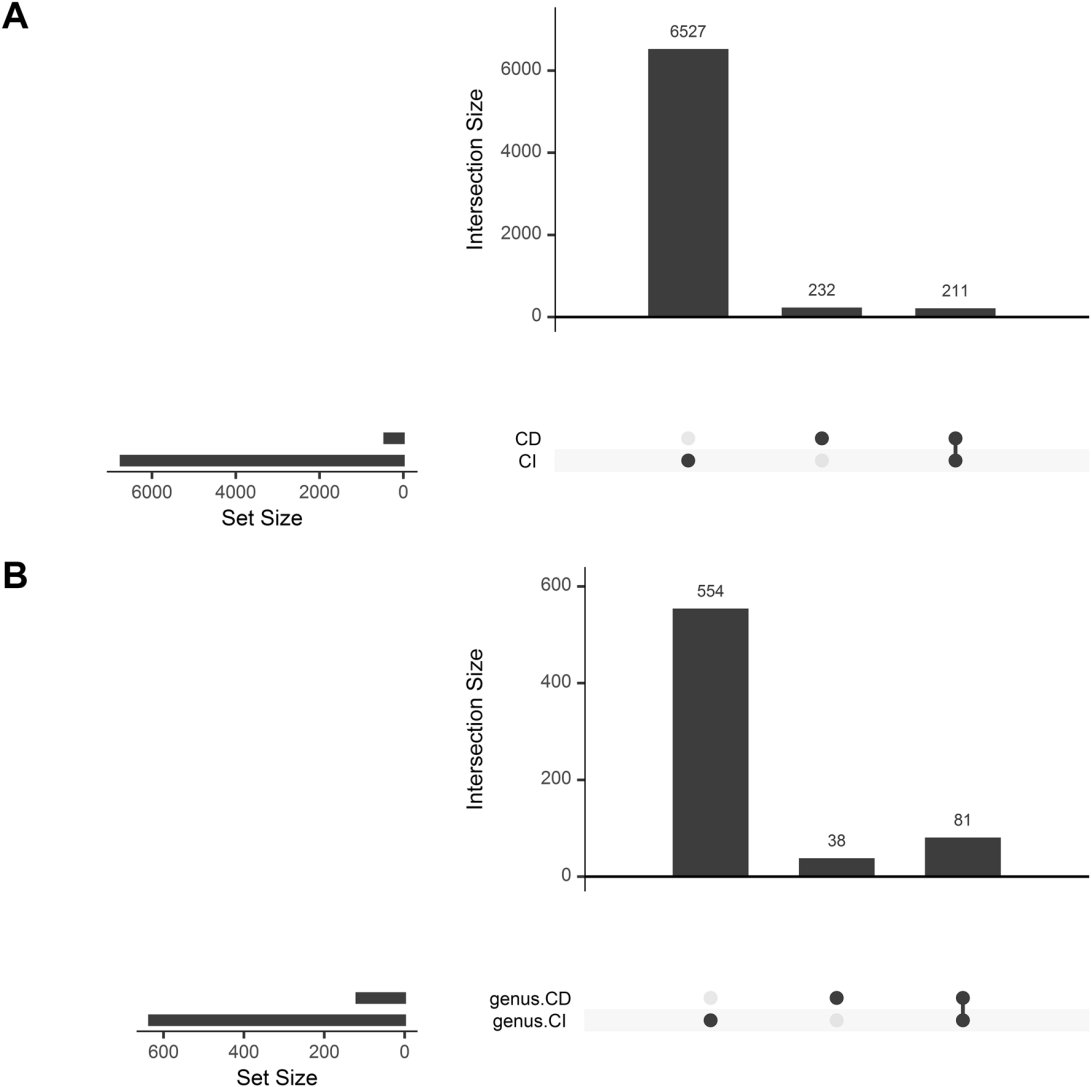
Upset plots representing shared and unique taxa. Upset plot displaying the distributions of (**A**) All detected ASVs and (**B**) Genera-level taxonomy, among CD and CI sample-groups.

A total of 6,738 and 443 ASVs were detected in CI and CD approaches, respectively (Figs. 8A, S8). Overall, 232 ASVs (3.32%) were detected exclusively in the culture-dependant approach compared to 6,527 ASVs (93.64%) detected exclusively in the culture-independent approach, while only 211 ASVs (3.04%) were shared among both approaches. PERMANOVA test on the Bray–Curtis distance of presence-absence matrix showed that CD and CI group of samples differ significantly (R^2^ = 0.21159, P = 0.000999) as well as based on different farms (R^2^ = 0.33404, P = 0.00099). When plotted through PCoA, the same result also highlighted these differences (Fig. S9).

Further, the taxonomy of these 232 ASVs present exclusively in the culture-dependent method was checked. CD-exclusive ASVs belonged to Proteobacteria (144), Firmicutes (42), Bacteroidetes (19), Actinobacteria (16), Firmicutes_A (4), Campylobacterota (3), Verrucomicrobia (2), Deinococcota and Firmicutes_B (1). At genus level, the greatest number of ASVs were of *Staphylococcus* (9%), unclassified member of *Pseudomonadaceace* (9%), *Enterobacteriacece* (7%) and *Burkholderiaceace* (4%) family followed by *Sterptococcus* (4%), *Enterbacter_D* (3%), *Acinetobacter* (3%) *Prevotella* (3%), *Sphingobacterium* (2%) (Fig. S10). Further, many of these ASVs were also assigned to the species level, such as *Pseudomonas furukawaii*, *Rhizobium pseudoryzae*, *Pseudomonas stutzeri* and many others. All these genera are also observed in notable amounts in the CI approach. However, many of the species were not observed in the CI approach.

To be more lenient, samples were further analyzed at the genus level to see if certain genera were only discovered in CD samples. A total of 635 and 119 genera were detected in CI and CD approaches, respectively, from 673 unique genera (Fig. 8B). Out of which 81 genera were common in both approaches, while 38 and 554 genera were exclusively present in CD and CI groups, respectively. These 38 genera were *Bacillus_AB, Bacteroides, Brachybacterium, Caminibacter, Cereibacter_A, Citrobacter, Corynebacterium, Deinococcus, Faecalibacterium, Helicobacter, Helicobacter_D, Heliorestis, Herbaspirillum, Lactobacillus, Lawsonibacter, Listeria, Micrococcus, Mixta, Moraxella_A, Neisseria, Paenibacillus, Paenibacillus_A, Paramesorhizobium, Pararheinheimera, Phocaeicola, Planobacterium, Prevotella, Pseudomonas_A, Pseudomonas_E, Rhizobium_A, SFEL01, Staphylococcus, Streptococcus, Tumebacillus, UBA1067, UBA3207,* and unknown member of *Enterococcaceae* and *UBA1067* family.

## Discussion

In this study, the peanut rhizospheric microbiota was analyzed using Illumina-based 16S rRNA gene sequencing and characterized the culturable bacterial diversity through traditional microbiological cultivation approach. The 16S rRNA gene sequencing technique is extensively used to characterize diverse microbiomes, including rhizosphere. The extensive use of new molecular methods is due to the limitation of traditional microbiological cultivation approaches which are unable to provide a complete picture of bacterial diversity due to the inability to cultivate all the microbes under laboratory conditions, probably because of their specific growth requirement. To provide more resolution to this analysis, the DADA2 denoising algorithm pipeline was used for data analysis^35^. DADA2 is a denoising algorithm designed particularly for Illumina data that infers ASVs based on single nucleotide changes, thus upholding strain-level information. In the present study, several ASVs were distinguished to species level (Fig. S6), including some ASVs with higher abundances. However, the analytical capacity is also affected by the database used for taxonomy assignment. For that, GTDB version 202 was used for the taxonomy assignment. Our choice of GTDB was influenced by the existing results that suggest more number of sequences annotating at the genus level in GTDB, and also because of the taxonomy lineage assignment approach used in GTDB^29,36,37^. GTDB is a curated database with comprehensive genome-based taxonomy based on monophyly and relative evolutionary divergence of taxa, which is an added advantage while annotating ASVs. The reclassifications by GTDB works well by distributing/reclassifying popular genera into several novel ones^36^. This gives a higher resolution to the observed organisms in this study. For example, the abundance of *Pseudomonas_F*, *Pseudomonas_M*, *Pseudomonas_R*, and *Pseudomonas_S* genera were observed among all *Pseudomonas* genera. Similar observations were also made with *Bacillus* genus where ASVs classified as *Bacillus_BD*, *Bacillus_AG*, *Bacillus_BN* and *Bacillu_BU* among all *Bacillus* genera.

In the present metagenomic study through the CI approach, the rhizospheric soils of F-08 farm showed higher Shannon diversity (6.92) than other farms, which may be due to influences of individual physico-chemical and abiotic parameters of respected rhizospheric soil. Previous studies showed that the electric conductivity and concentration of different nutrients including N, P and K may alter the diversity of microbial community present in rhizospheric soil^38–40^. Additionally, changes in soil pH and OC are typically linked to modification of rhizospheric microorganisms^20,41^. Our study found a strong significant link between OC, concentrations of P_2_O_5_ and Mn of rhizospheric soil with rhizospheric microbiota, different from previous studies where a significant link was found between pH, EC and concentration of K_2_O with rhizospheric microbiota^16,19,42^. In all CI samples, greater abundances (relative abundance ≥ 2%) of Proteobacteria, Acidobacteria (called Acidobacteriota in GTDB), Actinobacteria (named Actinobacteriota in GTDB), Planctomycetes (named Planctomycetota in GTDB), Firmicutes, Bacteroidetes (called Bacteroidota in GTDB), and Verrucomicrobia (named Verrucomicrobiota in GTDB) were found as compared to other phyla. Several previous investigations have found a greater abundance of Firmicutes (approximately 3 to 7%) in the rhizosphere^14,18,25,43^ including a study on peanut microbiome^28^. However, this was not observed in other studies on the peanut rhizosphere^27,44^. In the present study, the most abundant genera are *Sphingomicrobium*, *UBA2421, Aureimonas _A,* unknown member from *Sphingomonadaceae*, *UBA1161* family and Bacilli class, and genus of unknown bacterium. The genera *UBA2421* and *UBA1161*, observed with > 2% abundance in all samples belong to Planctomycetota phylum and are yet uncharacterized organisms. These representative genera are still not reported by cultivable approach and have only been described in the Metagenome assembled genome (MAG) database^45^. Databases like GTDB, which contains many MAGs can be an added advantage of observing an accurate depiction of diversity and illustrates the fact that there are numerous more abundant microorganisms whose roles in the ecosystem have yet to be determined.

One of the objectives of the present study is to observe the rhizospheric community among all samples by considering natural abiotic stress conditions and to find out changes in microbial community and how it represents the core microbiome of peanut plants by using CI approach. While in previous studies, the rhizosphere community of peanut plants was evaluated under controlled environment (in greenhouse), which gives a limited idea of the rhizospheric community^27,28,44^. In this study, the topmost abundant genera were common in each rhizospheric soil sample (Fig. 2B, Table S3). Moreover, the core microbiome was further studied to investigate the potential plant growth-promoting genera among rhizospheric soil samples. Previous studies shows that, as per different plant growth stages, plants release various exudates, which modify surrounding rhizobacterial populations by selecting the finest organisms that can aid in promotion of plant development in various ways, those are commonly called as Plant growth promoting bacteria (PGPB). PGPB can colonize the rhizosphere and form close relationships with roots of host plant^46,47^. The beneficial effects of PGPB on plant growth are achieved through direct mechanisms such as facilitating nutrient uptake, like primarily nitrogen and phosphorus, and by producing phytohormones. Genera like *Sphingomicrobium, Actinoplanes, Aureimonas _A, Chryseobacterium,* members from *Sphingomonadaceae*, *Burkholderiaceae*, *Pseudomonadaceae* family and Bacilli class were observed in almost all samples. All of these are reported to show PGP activities. For example, *Sphingomonadaceae* and *Burkholderiaceae* family members are well studied for their antifungal activity against *Rhizoctonia solani*, which is the primary plant pathogen in peanuts^48–50^, *Actinoplanes* reported to possess IAA production, siderophore production, and ACC deaminase activity^51,52^ while *Bacillus* and *Pseudomonas* are reported to possess several beneficial activities including solubilization of phosphate, nitrogen fixation and siderophore production^53,54^. *Aureimonas _A* is member of the *Rhizobiaceae* family known for important nitrogen-fixing symbionts of plants. Based on rhizospheric bacterial diversity of the core microbiome, a potential biofertilizer was formulated to check the effect of biofertilizer to promote the growth of different variety of peanut plants (unpublished data). Some species-level assignments of those genera’s representatives were also observed in the CI approach, like *Sphingomicrobium sp003097155, Pseudoduganella eburnean, Ectobacillus funiculus, Metabacillus sp002871465*, *Pseudomonas_M indica* and *Bacillus_BD endozanthoxylicus* (Fig. S6).

Further bacterial diversity was also characterized by comparing the CD approach with the CI approach, by doing so expecting to get a complete idea about the peanut bacterial diversity as although metagenomics is extremely popular, it also fails to reflect the true diversity present in the sample due to some of its limitations. For example, if microorganisms are present in very low abundance it may be left out during DNA extraction or possible bias to amplify the target DNA, data analysis pipeline, and database, all of which affect the final interpretation of the results^55^. As noted in method, in this work, all the colonies were taken away from the medium and relied on NGS-based metagenomic platform for identification and analysis. This should reflect almost the entire cultivable diversity, including several microcolonies. By doing so, we have incorporated sequences from all the organisms grown on plates rather than imposing selection biases based on colony observation/morphology.

Further, using various media, generating a high amount of data, and considering enough reads for the analysis can help to provide a complete picture of bacterial diversity. Furthermore, for comparative study, partial 16S rRNA gene was sequenced, similar to metagenomics, rather than sequencing the entire 16S rRNA gene, through Sanger sequencing as done in all previous researches^32–34^. As per our knowledge, a similar approach has also been successfully applied by Zehavi et al. for the study of ruminal microbiota and in our previous study on rhizosphere microbiome^56,57^. This approach could be helpful to analyze the presence-absence based study of microbial diversity. However, it is not appropriate when attempting to analyze the abundance of cultivable organisms. Also, it would not be possible to separate and purify the colony on the media if needed for further experiments. According to the majority of research, the CI strategy has greater diversity than the CD technique^33,34^. Similar conclusions can also be drawn from this study as well. Total of 232 ASVs exclusively present in CD samples. This might be due to the very low abundance of those organisms, which is a limitation of CI approach. Comparing all the genera of both the approaches, revealed that total 38 genera were exclusively found in CD samples, that is similar to the studied by Hinsu et al.^57^. Most of these genera are commonly found in soil, forest and water sources including marine water. A few of the genera, like *Helicobacter*, *Heliorestis* and *Herbaspirillum* are also linked to nitrogen fixation ability as per some recent studies^58,59^. Surprisingly, the *UBA1067* family and *UBA3207* genus from the Kiritimatiellae and Bacilli class respectively were also observed, which are candidate taxa with no cultivated representative as of yet.

## Methods and materials

### Experimental design and sample collection

The rhizospheric soil samples were collected from 13 different farms covering 5 districts of Saurashtra region of Gujarat, INDIA, in 2019 (detailed information in Table 1). All the farms have history of continuously sowing of G-20 variety of Groundnut (*Arachis hypogaea* L.) during the cropping season. All the rhizospheric soil samples were collected within 10 days to avoid differences in crop stages at the nodulation phase of crops. All the farms had sowed the seed almost at the same time, to take advantage of rains by hurricane, hence being a benefit for our study. From each farm, 5 plant samples were randomly selected as replicates and uprooted gently by removing nearby soil. The plants were vigorously shaken to remove loosely attached soil. Afterward, the tightly adhering soil on the root surface was collected in sterile container for rhizospheric soil property determination. The roots with tightly adhering rhizospheric soil were washed in sterile normal saline (1% NaCl) in a flask, and the washed soil was then collected in sterile 50 ml falcon tubes for microbiome analysis^24,33^. Total 5 g rhizospheric soil from all 5 replicates of the same farm was pooled separately in new sterile tube to study bacterial diversity by culture-dependent approach. Same practice was done for all farms. The rhizospheric soil samples for property estimation were transported at room temperature, and samples for microbiome work were transported to lab at 4 °C and then stored at − 20 °C till further processing. Overall, 65 samples of 13 farms were analyzed for metagenomics study, and comparative analysis of CI and CD approach (Fig. S11).

### Sample processing

The rhizospheric soil samples were sent for physicochemical examination to a government-approved soil testing laboratory (Gujarat State Fertiliser Company, GSFC, Vadodara, INDIA). The samples were tested for physical properties (pH and electrical conductivity), macronutrients (% organic carbon, concentrations of phosphate and potassium) and micronutrients (concentrations of iron, sulfur, manganese, zinc, and copper).

For the rhizosphere microbiome (CI approach), the rhizospheric samples were thawed and homogenized. The tubes were then centrifuged at 12,000 rpm for 10 min. At this speed, all microbial cells, along with soil particles, will settle down, leaving behind buffer in supernatant which was discarded. After carefully mixing the soil, it was immediately used for DNA extraction^24^. DNA was extracted from 1 g of soil using Qiagen PowerSoil DNA Extraction kit (Qiagen, Germany) following the manufacturer’s instructions.

For the CD approach, samples were serially diluted and plated on eight different media supplemented with cycloheximide (50µg/ml) on the next day of collection (Table S5). 10^–4^ and 10^–5^ dilutions were used for spreading, and plates were incubated at 27 ± 2 °C and 37 ± 2 °C in triplicates. After incubation for 15 days, all the colonies of the same samples were scrapped from each media, collected in phosphate buffer, and mixed. DNA was extracted from this pool of colonies using QIAGEN QIAamp DNA Mini Kit (Qiagen, Germany) following manufacturer’s protocol of bacterial genomic DNA extraction (Fig. S12). Extracted DNA was checked on agarose gel for good quality and quantified using Qubit 3.0 (Invitrogen, CA).

### Library preparation and sequencing

The 16S rRNA gene amplicon sequencing libraries were prepared separately for CI and CD from 12.5 ng DNA as starting material following double-pass PCR protocol as given in Illumina 16S library preparation guide (Illumina, USA). The primers 341F and 785R coupled with Illumina adapters were used to target the V3-V4 region of the 16S rRNA gene^60^. Agilent Bioanalyser (Agilent, USA) was used to validate the libraries, and Qubit v3 was used to quantify them (ThermoFisher Scientific, USA). The libraries were sequenced separately for CI (run1) and CD (run2) approaches on Illumina MiSeq using 250 × 2 v2 chemistry.

### Data analysis

The raw fastq data was analyzed using the Divisive Amplicon Denoising Algorithm 2 (DADA2) pipeline (“dada2” package version 1.22) in R v4.1.3 following the steps given at https://benjjneb.github.io/dada2/tutorial.html for rhizosphere microbiome^35,61,62^. Further, CI and CD samples were sequenced in different runs. Runs of CI and CD were processed independently until sequence table generation and then merged for further steps as indicated in the "big data" tutorial (https://benjjneb.github.io/dada2/bigdata.html) for comparative analysis of bacterial diversity. Taxonomy of ASVs was assigned using GTDB v202 databases using the files hosted at zenodo^63^.

The downstream analysis was done using Phyloseq package v1.38.0 in R v4.1.3 along with other packages like Microbiome v1.16, ggpubr v0.4, vegan v2.6–2^62,64–67^. In a sense, all of the data was loaded into a Phyloseq object. The "alpha" function from the Microbiome package was used to compute alpha diversity. Non-Metric Dimensional Scaling (NMDS) on Bray–Curtis distance was computed and plotted using the methods in the Phyloseq package to examine beta diversity. Adonis() function from vegan package and pairwise.adonis() function from pairwiseAdonis package v0.4 was used to compare Bray–Curtis distances among groups^68^. The Phyloseq package was used to agglomerate taxonomy at the phylum and genus levels. ggpubr package was used for comparing statistical differences among different groups. Core-microbiome was determined using the functions in Microbiome package. All the visualisations were prepared in R using ggplot2 package v3.3.6 along with other packages ggpubr v0.4, ggConvexHull v0.1.0, ggnewscale v0.4.7 and ggrepel v0.9.1^69–72^. Other R packages data.table v1.14.2, randomcoloR v1.1.0.1, tidyr v1.2.0, scales v1.2.0, rstatix v0.7.0 and RColorBrewer v1.1–3 were also used in the analysis^73–78^.

### Ethics declaration

The study included the use of soil associated with plants. No ethical approval was required for the investigation because no plant components were used. Additionally, the owner or farmer was made aware of the research and the kinds of samples that would be taken. Verbal consent and permission were obtained to collect the soil from his farm for the work.

## Conclusion

The findings of the current study indicated that a large number of uncultured and unidentified core bacterial genera representative were present in the peanut rhizospheric, many of which may have interacted with the host plant and other microorganisms. Additionally, key core genera that were known to support plant growth were identified from the peanut rhizosphere; this knowledge helped us develop efficient bio-strategies, such bio-fertilizer. We were able to obtain a comprehensive understanding of the bacterial diversity of the peanut rhizosphere in its native environmental circumstances by comparing the two methods (CI and CD approaches) in detail. However, functional metagenomics provide a bigger picture, but the current study could not examine it due to a lack of resources.

### Supplementary Information

Supplementary Information.

## Data Availability

The R script used for analysis is available from github.com/krunal1704/peanut-rhizosphere (10.5281/zenodo.8307544) to reproduce the entire work. The raw data files can be downloaded from the NCBI SRA (Accessions SRR19850516 to SRR19850603) under Bioproject PRJNA851912.

## References

[1] Edwards J (2015). Structure, variation, and assembly of the root-associated microbiomes of rice. Proc. Natl. Acad. Sci. USA.

[2] Liu F (2019). Soil indigenous microbiome and plant genotypes cooperatively modify soybean rhizosphere microbiome assembly. BMC Microbiol..

[3] Vives-Peris V, de Ollas C, Gomez-Cadenas A, Perez-Clemente RM (2020). Root exudates: From plant to rhizosphere and beyond. Plant Cell Rep..

[4] Mendes R, Garbeva P, Raaijmakers JM (2013). The rhizosphere microbiome: Significance of plant beneficial, plant pathogenic, and human pathogenic microorganisms. FEMS Microbiol. Rev..

[5] Qu Q (2020). Rhizosphere microbiome assembly and its impact on plant growth. J. Agric. Food Chem..

[6] Bulgarelli D (2015). Structure and function of the bacterial root microbiota in wild and domesticated barley. Cell Host Microbe.

[7] Cordero J, de Freitas JR, Germida JJ (2020). Bacterial microbiome associated with the rhizosphere and root interior of crops in Saskatchewan, Canada. Can. J. Microbiol..

[8] Leoni C (2020). Plant health and rhizosphere microbiome: Effects of the bionematicide aphanocladium album in tomato plants infested by *Meloidogyne javanica*. Microorganisms.

[9] Lundberg DS (2012). Defining the core *Arabidopsis thaliana* root microbiome. Nature.

[10] Schmidt JE, Kent AD, Brisson VL, Gaudin ACM (2019). Agricultural management and plant selection interactively affect rhizosphere microbial community structure and nitrogen cycling. Microbiome.

[11] Vitulo N (2018). Bark and grape microbiome of *Vitis vinifera*: Influence of geographic patterns and agronomic management on bacterial diversity. Front. Microbiol..

[12] Baudoin E, Benizri E, Guckert A (2002). Impact of growth stage on the bacterial community structure along maize roots, as determined by metabolic and genetic fingerprinting. Appl. Soil Ecol..

[13] Bhattarai A, Bhattarai B, Pandey S (2015). Variation of soil microbial population in different soil horizons. J. Microbiol. Exp..

[14] DeAngelis KM (2009). Selective progressive response of soil microbial community to wild oat roots. ISME J..

[15] Ding LJ (2019). Microbiomes inhabiting rice roots and rhizosphere. FEMS Microbiol. Ecol..

[16] Fan K (2017). Rhizosphere-associated bacterial network structure and spatial distribution differ significantly from bulk soil in wheat crop fields. Soil Biol. Biochem..

[17] Hu J (2020). Rhizosphere microbiome functional diversity and pathogen invasion resistance build up during plant development. Environ. Microbiol..

[18] Jaiswal SK, Mohammed M, Dakora FD (2019). Microbial community structure in the rhizosphere of the orphan legume Kersting's groundnut [*Macrotyloma geocarpum* (Harms) Marechal & Baudet]. Mol. Biol. Rep..

[19] Kuramae EE (2012). Soil characteristics more strongly influence soil bacterial communities than land-use type. FEMS Microbiol. Ecol..

[20] Lauber CL, Hamady M, Knight R, Fierer N (2009). Pyrosequencing-based assessment of soil pH as a predictor of soil bacterial community structure at the continental scale. Appl. Environ. Microbiol..

[21] Mendes LW, Kuramae EE, Navarrete AA, van Veen JA, Tsai SM (2014). Taxonomical and functional microbial community selection in soybean rhizosphere. ISME J..

[22] Peiffer JA (2013). Diversity and heritability of the maize rhizosphere microbiome under field conditions. Proc. Natl. Acad. Sci. USA.

[23] Perez-Jaramillo JE (2019). Deciphering rhizosphere microbiome assembly of wild and modern common bean (*Phaseolus vulgaris*) in native and agricultural soils from Colombia. Microbiome.

[24] Qiao Q (2017). The variation in the rhizosphere microbiome of cotton with soil type, genotype and developmental stage. Sci. Rep..

[25] Sugiyama A, Ueda Y, Zushi T, Takase H, Yazaki K (2014). Changes in the bacterial community of soybean rhizospheres during growth in the field. PLoS ONE.

[26] Xu J (2018). The structure and function of the global citrus rhizosphere microbiome. Nat. Commun..

[27] Dai L (2019). Effect of drought stress and developmental stages on microbial community structure and diversity in peanut rhizosphere soil. Int. J. Mol. Sci..

[28] Haldar S, Sengupta S (2015). Impact of plant development on the rhizobacterial population of *Arachis hypogaea*: A multifactorial analysis. J. Basic Microbiol..

[29] Hinsu AT, Panchal KJ, Pandit RJ, Koringa PG, Kothari RK (2021). Characterizing rhizosphere microbiota of peanut (*Arachis hypogaea* L.) from pre-sowing to post-harvest of crop under field conditions. Sci. Rep..

[30] Donachie SP, Foster JS, Brown MV (2007). Culture clash: Challenging the dogma of microbial diversity. ISME J..

[31] Shade A (2012). Culturing captures members of the soil rare biosphere. Environ. Microbiol..

[32] Hiergeist A, Gläsner J, Reischl U, Gessner A (2015). Analyses of intestinal microbiota: Culture versus sequencing. ILAR J..

[33] Lee S (2016). Comparative analysis of bacterial diversity in the rhizosphere of tomato by culture-dependent and-independent approaches. J. Microbiol..

[34] Youseif SH (2021). Comparative analysis of the cultured and total bacterial community in the wheat rhizosphere microbiome using culture-dependent and culture-independent approaches. Microbiol. Spectrum.

[35] Callahan BJ (2016). DADA2: High-resolution sample inference from Illumina amplicon data. Nat. Methods.

[36] Parks DH (2020). A complete domain-to-species taxonomy for Bacteria and Archaea. Nat. Biotechnol..

[37] Parks DH (2018). A standardized bacterial taxonomy based on genome phylogeny substantially revises the tree of life. Nat. Biotechnol..

[38] Wu N, Li Z, Meng S, Wu F (2021). Soil properties and microbial community in the rhizosphere of *Populus alba* var. pyramidalis along a chronosequence. Microbiol. Res..

[39] Wang L (2023). Rhizosphere soil nutrients and bacterial community diversity of four broad-leaved trees planted under Chinese fir stands with different stocking density levels. Front. For. Glob. Change.

[40] Karak T, Bhattacharyya P, Paul RK, Das D (2013). Metal accumulation, biochemical response and yield of Indian mustard grown in soil amended with rural roadside pond sediment. Ecotoxicol. Environ. Saf..

[41] Lareen A, Burton F, Schafer P (2016). Plant root-microbe communication in shaping root microbiomes. Plant Mol. Biol..

[42] Enagbonma BJ, Ajilogba CF, Babalola OO (2020). Metagenomic profiling of bacterial diversity and community structure in termite mounds and surrounding soils. Arch. Microbiol..

[43] Correa-Galeote D, Bedmar EJ, Fernandez-Gonzalez AJ, Fernandez-Lopez M, Arone GJ (2016). Bacterial communities in the rhizosphere of amilaceous maize (*Zea mays* L.) as assessed by pyrosequencing. Front. Plant Sci..

[44] Xu Y (2020). Influence of salt stress on the rhizosphere soil bacterial community structure and growth performance of groundnut (*Arachis hypogaea* L.). Int. Microbiol..

[45] Parks DH (2017). Recovery of nearly 8,000 metagenome-assembled genomes substantially expands the tree of life. Nat. Microbiol..

[46] Berendsen R, Pieterse CJ, Bakker PAHM (2012). The rhizosphere microbiome and plant health. Trends Plant Sci..

[47] Revolti LTM, Caprio CH, Mingotte FLC, Môro GV (2018). *Azospirillum* spp. potential for maize growth and yield. Afr. J. Biotechnol..

[48] Chapelle E, Mendes R, Bakker PA, Raaijmakers JM (2016). Fungal invasion of the rhizosphere microbiome. ISME J..

[49] Glaeser, S. P. & Kämpfer, P. The family sphingomonadaceae. In *The Prokaryotes: Alphaproteobacteria and Betaproteobacteria*, 641–707 (2014).

[50] Gómez Expósito R, De Bruijn I, Postma J, Raaijmakers JM (2017). Current insights into the role of rhizosphere bacteria in disease suppressive soils. Front. Microbiol..

[51] Hamedi J, Mohammadipanah F (2015). Biotechnological application and taxonomical distribution of plant growth promoting actinobacteria. J. Ind. Microbiol. Biotechnol..

[52] Conn VM, Franco CM (2004). Analysis of the endophytic actinobacterial population in the roots of wheat (*Triticum aestivum* L.) by terminal restriction fragment length polymorphism and sequencing of 16S rRNA clones. Appl. Environ. Microbiol..

[53] Hashem A, Tabassum B, Fathi Abd Allah E (2019). *Bacillus subtilis*: A plant-growth promoting rhizobacterium that also impacts biotic stress. Saudi J. Biol. Sci..

[54] Etesami H, Maheshwari DK (2018). Use of plant growth promoting rhizobacteria (PGPRs) with multiple plant growth promoting traits in stress agriculture: Action mechanisms and future prospects. Ecotoxicol. Environ. Saf..

[55] Laudadio I, Fulci V, Stronati L, Carissimi C (2019). Next-generation metagenomics: Methodological challenges and opportunities. Omics.

[56] Zehavi T, Probst M, Mizrahi I (2018). Insights into culturomics of the rumen microbiome. Front. Microbiol..

[57] Hinsu A (2021). To culture or not to culture: A snapshot of culture-dependent and culture-independent bacterial diversity from peanut rhizosphere. PeerJ.

[58] Dewey ED (2020). Analysis of the complete genome of the alkaliphilic and phototrophic firmicute *Heliorestis convoluta* strain HHT. Microorganisms.

[59] Monteiro RA (2012). Herbaspirillum-plant interactions: Microscopical, histological and molecular aspects. Plant Soil.

[60] Klindworth A (2013). Evaluation of general 16S ribosomal RNA gene PCR primers for classical and next-generation sequencing-based diversity studies. Nucleic Acids Res..

[61] Callahan BJ, Sankaran K, Fukuyama JA, McMurdie PJ, Holmes SP (2016). Bioconductor workflow for microbiome data analysis: From raw reads to community analyses. Research.

[62] Team, R. C. R Core Team R: a language and environment for statistical computing. *Foundation for Statistical Computing* (2020).

[63] Alishum, A. DADA2 formatted 16S rRNA gene sequences for both bacteria & archaea. *Res. Data* (2019).

[64] McMurdie PJ, Holmes S (2013). phyloseq: An R package for reproducible interactive analysis and graphics of microbiome census data. PLoS ONE.

[65] Kassambara, A. ggpubr:‘ggplot2’based publication ready plots. R package version 0.4. 0. *.* (2020).

[66] Jari Oksanen, F. *et al.* Vegan: community ecology package. *R package version* **2** (2018).

[67] Lahti, L. & Shetty, S. Tools for microbiome analysis in R. 2017. http://microbiome.github.com/microbiome (2017).

[68] Martinez Arbizu, P. pairwiseAdonis: Pairwise multilevel comparison using adonis. *R package version 0.4* **1** (2020).

[69] Wickham, H. *ggplot2: elegant graphics for data analysis*. (springer, 2016).

[70] Martin, C. ggConvexHull: Add a convex hull geom to ggplot2. *R package version 0.1. 0* (2017).

[71] Campitelli, E. ggnewscale: Multiple Fill and Colour Scales in “ggplot2”. *R package version 0.4* **1** (2020).

[72] Slowikowski, K. *et al.* Automatically position non-overlapping text labels with ‘ggplot2’. *R Package Version 0.9* **1** (2021).

[73] Dowle, M., Srinivasan, A. & Gorecki, J. data. table: Extension of ‘data. frame’. R Package Version 1.12. 8. *Manual* (2019).

[74] Ammar R (2019). randomcoloR: Generate attractive random colors. R package version.

[75] Wickham, H. Tidyr: Tidy Messy Data: R Package Version 1.1. 3. 2021. https://CRAN.R-project.org/package=tidyr (2021).

[76] Wickham H, Seidel D (2020). scales: Scale functions for visualization. R package version.

[77] Kassambara, A. rstatix: pipe-friendly framework for basic statistical tests. R package version 0.7. 0. *Computer software]. *https://CRAN.R-project.org/package=rstatix (2021).

[78] Neuwirth, E. RColorBrewer: ColorBrewer Palettes. R package version 1.1-3. https://CRAN.R-project.org/package=RColorBrewer (2022).

