# Supplementary material for: Metagenomic evaluation of peanut rhizosphere microbiome from the farms of Saurashtra regions of Gujarat, India
Source: Sci Rep. 2024 May 8;14:10525. doi: 10.1038/s41598-024-61343-5 (PMC11079051; doi:10.1038/s41598-024-61343-5)
Supplement: Supplementary file 1 — Supplementary Information. [file 41598_2024_61343_MOESM1_ESM.docx]

**Metagenomic evaluation of peanut rhizosphere microbiome from the farms of Saurashtra regions of Gujarat, India**

Krunal R. Hirpara^1^, Ankit T. Hinsu^1#^ and Ramesh K. Kothari^1*^

^1^ Department of Biosciences, Saurashtra University, Rajkot, Gujarat, India

^#^ Current address: Royal Veterinary College, London, UK AL9 7TA

E-mail addresses of authors:

Krunal R. Hirpara: krunalhirrpara17@gmail.com

Ankit T. Hinsu: ankit4035hinsu@gmail.com

Ramesh K. Kothari: kothari1971@gmail.com

* Correspondence address:

Prof. Ramesh K. Kothari,

Department of Biosciences,
Saurashtra University,
Rajkot, Gujarat, India – 360005
[kothari1971@gmail.com](mailto:kothari1971@gmail.com), [rkkothari@sauuni.ac.in](mailto:rkkothari@sauuni.ac.in)

**Supplementary figures**


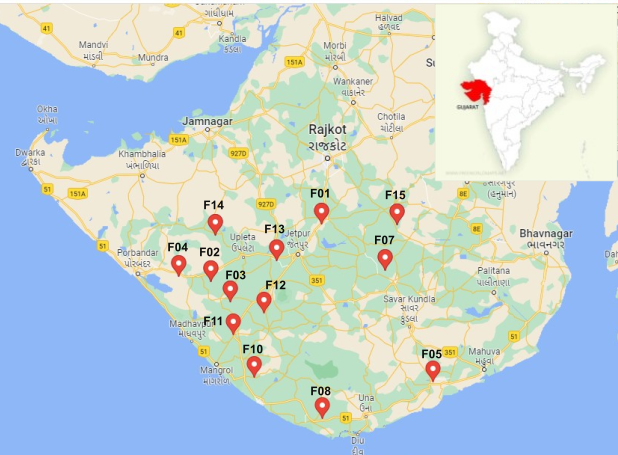


**Figure S1**: Location of all the farm sites sampled in the study. (Source: https://www.google.com/maps)


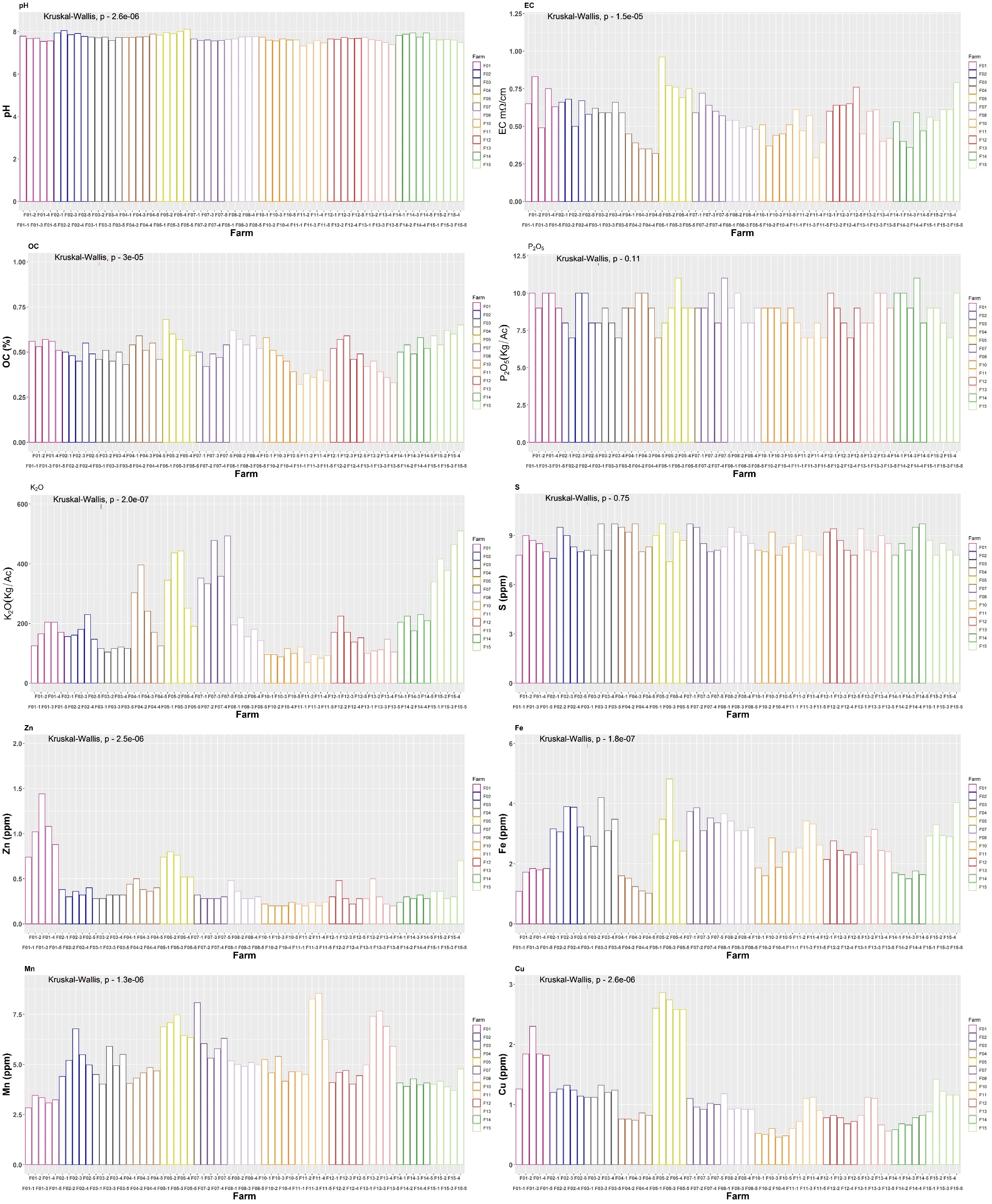


**Figure S2**: Comparisons of environmental parameters and nutrient concentration of all farm’s samples. p-value from Kruskal–Wallis test comparing all farms is mentioned on the top.


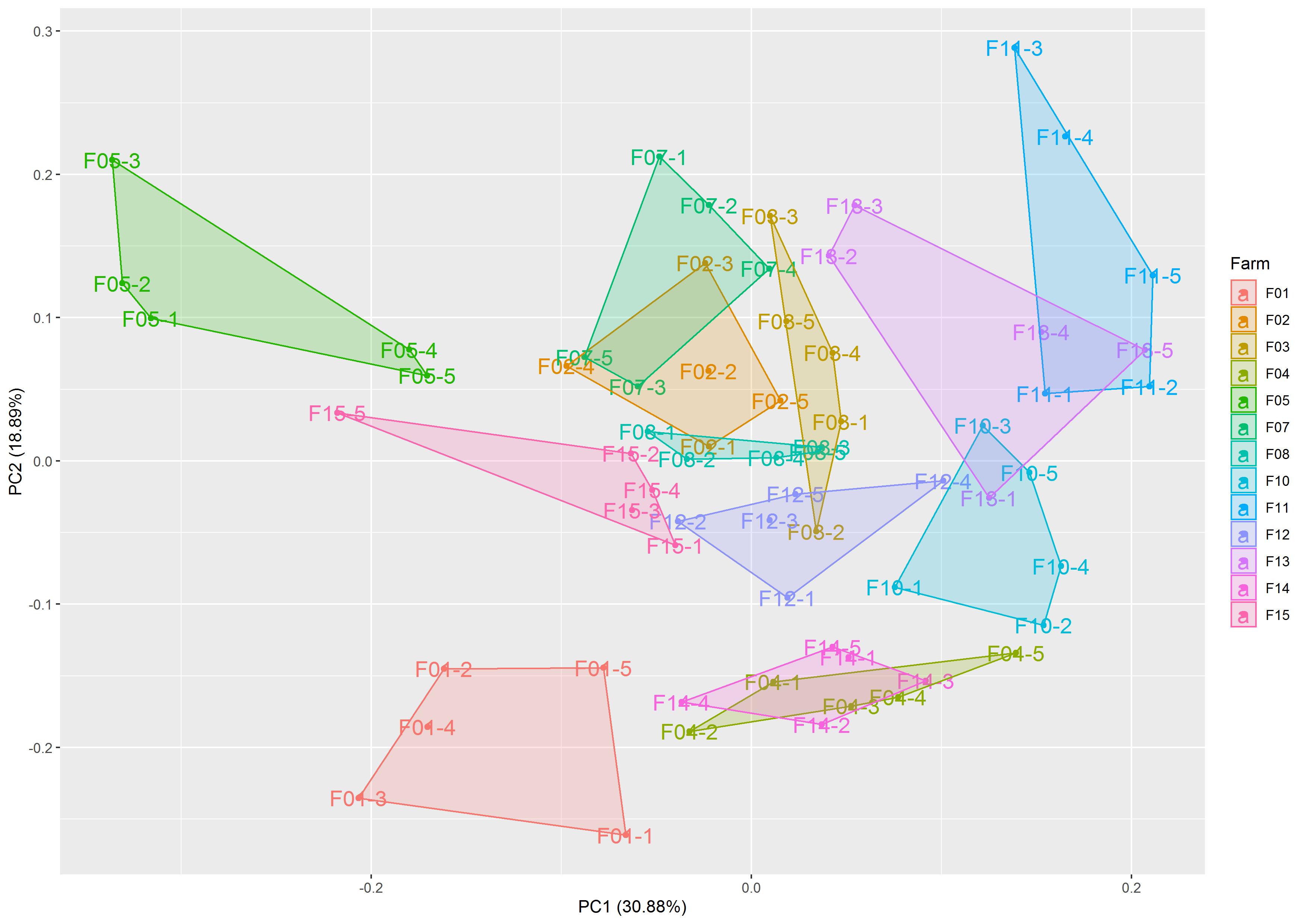


**Figure S3**: PCA plot showing distribution of samples according to the farms.


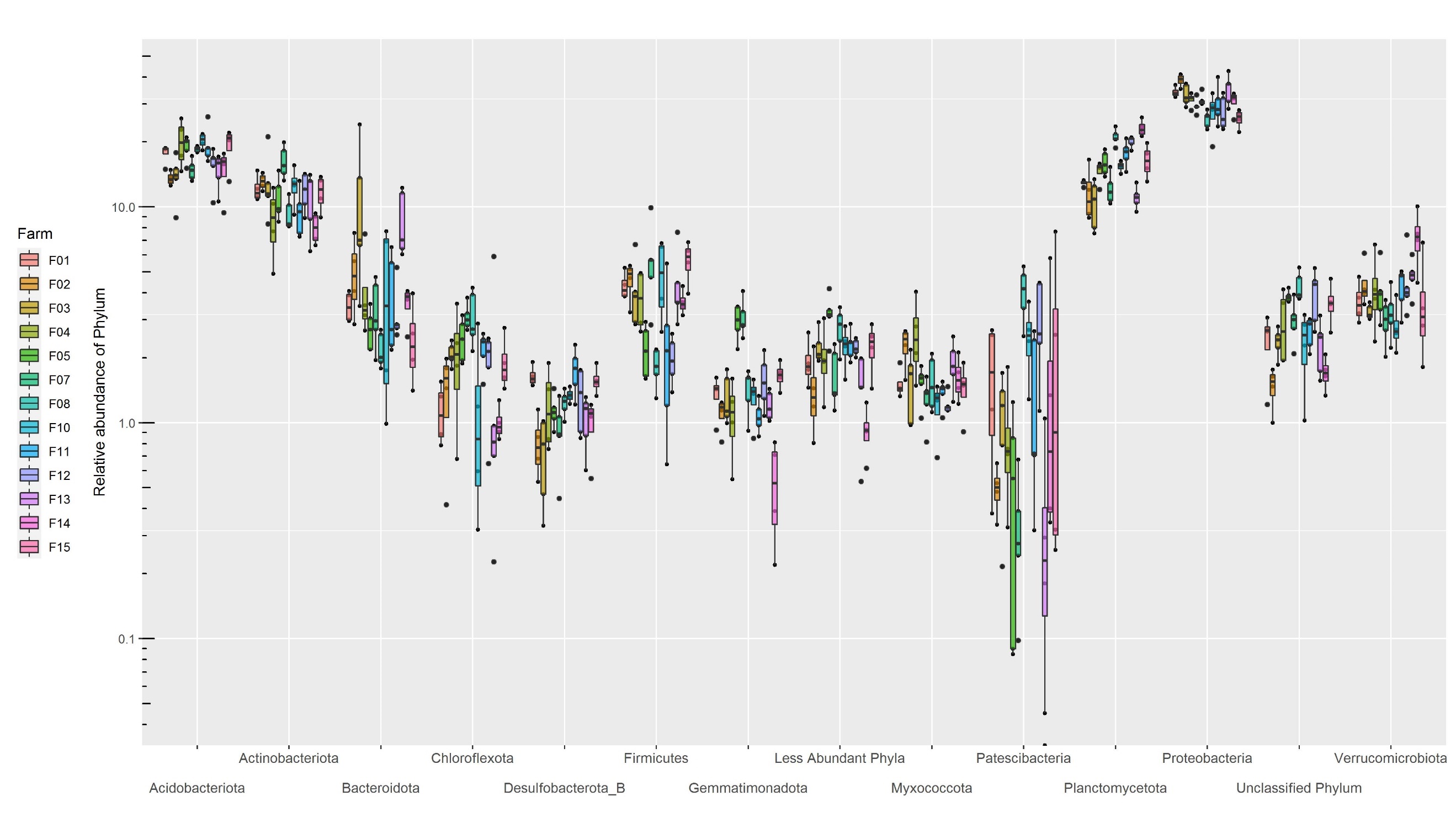


**Figure S4**: Distribution of all rhizosphere samples at the phylum level. Phyla with an abundance greater than 1% in at least one sample are plotted.


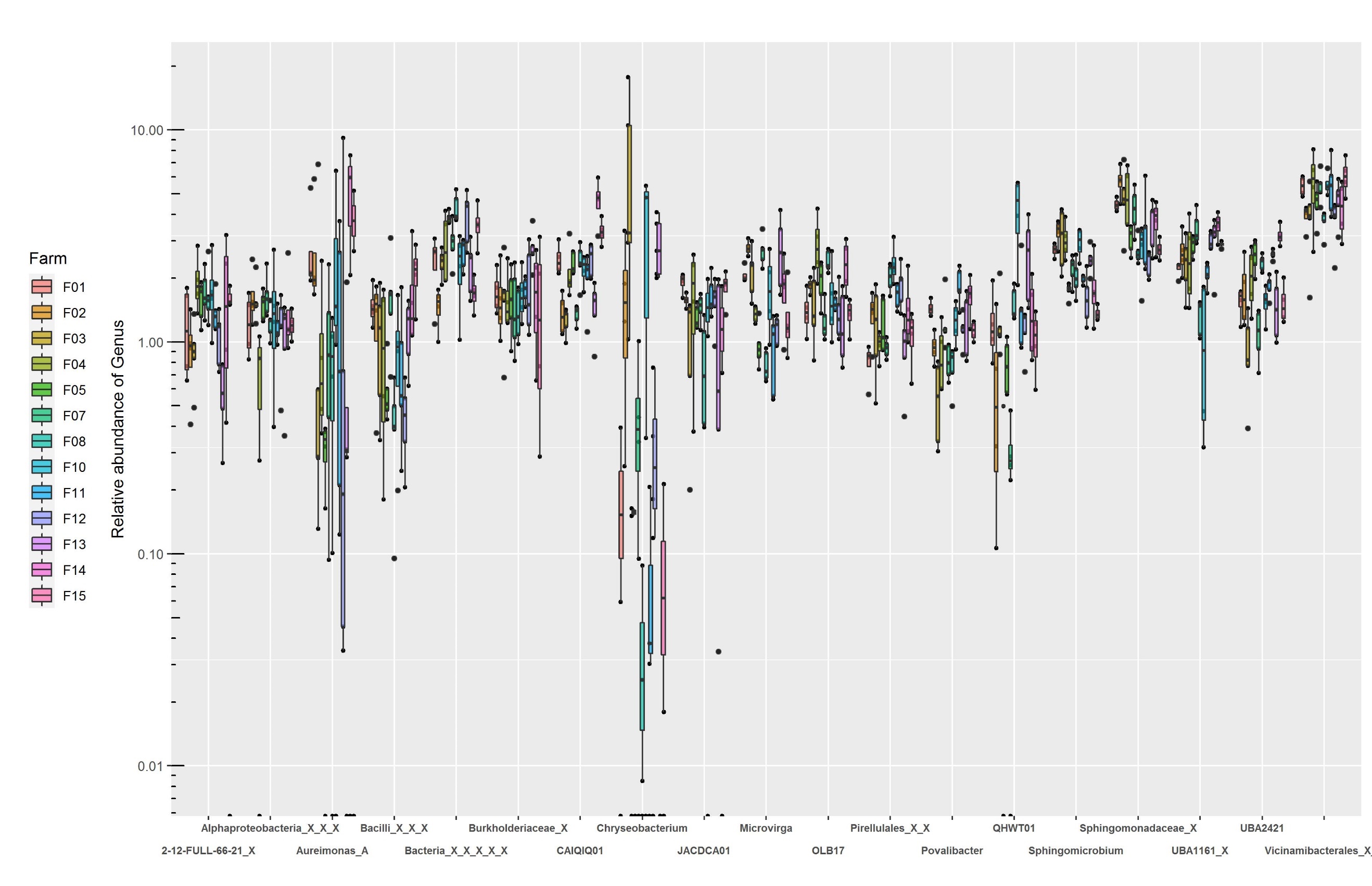


**Figure S5**: Distribution of all rhizosphere samples at the Genus level. Genus with an abundance greater than 1% in at least one sample are plotted.


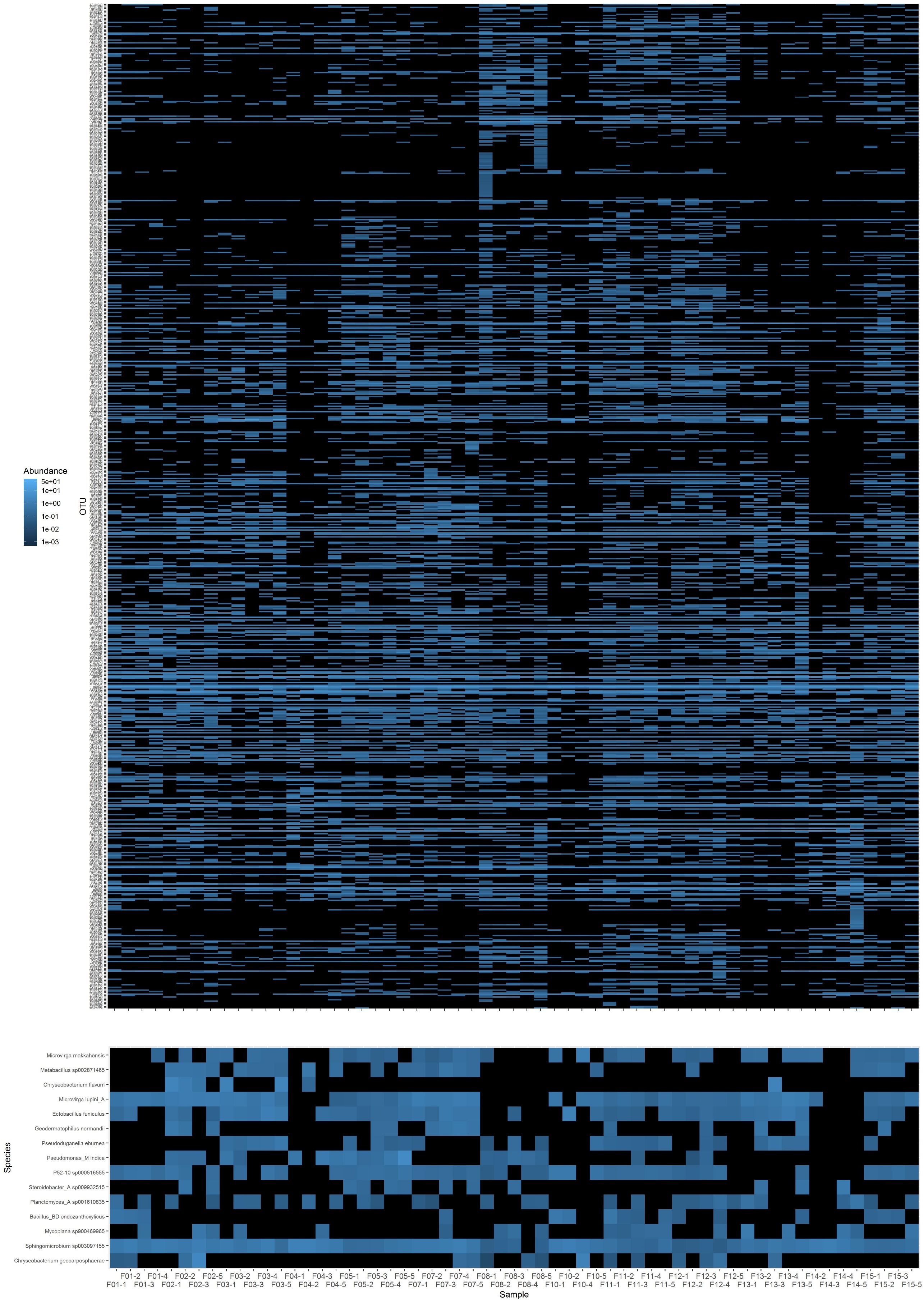


**Figure S6**: Heatmap representing abundance distribution for all ASV (top) and top ASVs annotated as species (bottom).


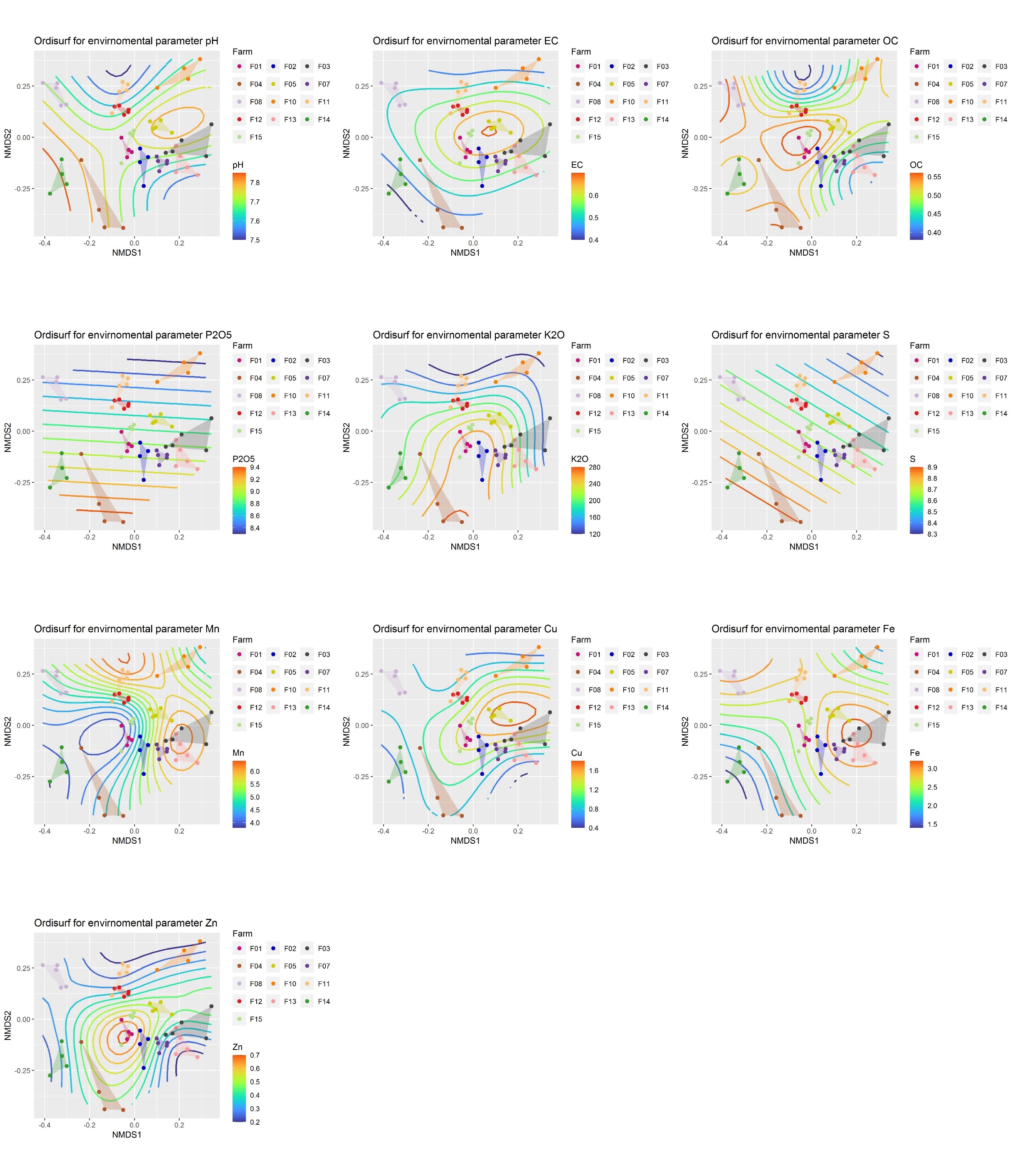


**Figure S7**: Ordisurf plots for all environmental variable and alpha diversity metrics overlapped on NMDS plot of all samples.


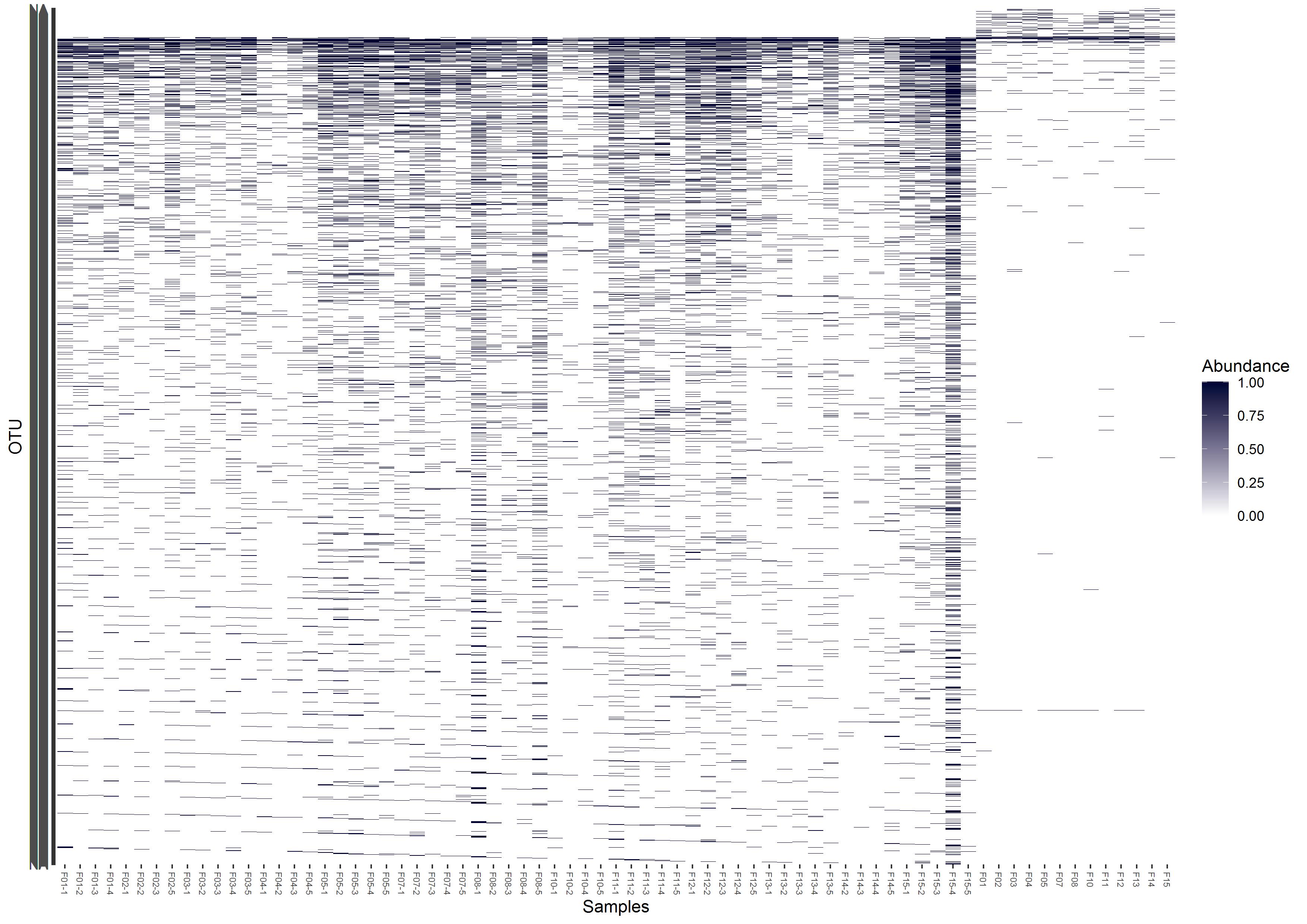


**Figure S8**: Heatmap showing presence-absence of ASVs. Colour represents presence while no colour (white colour) represents absence of ASVs. Top section of ASVs is observed exclusively in culture-dependent data.


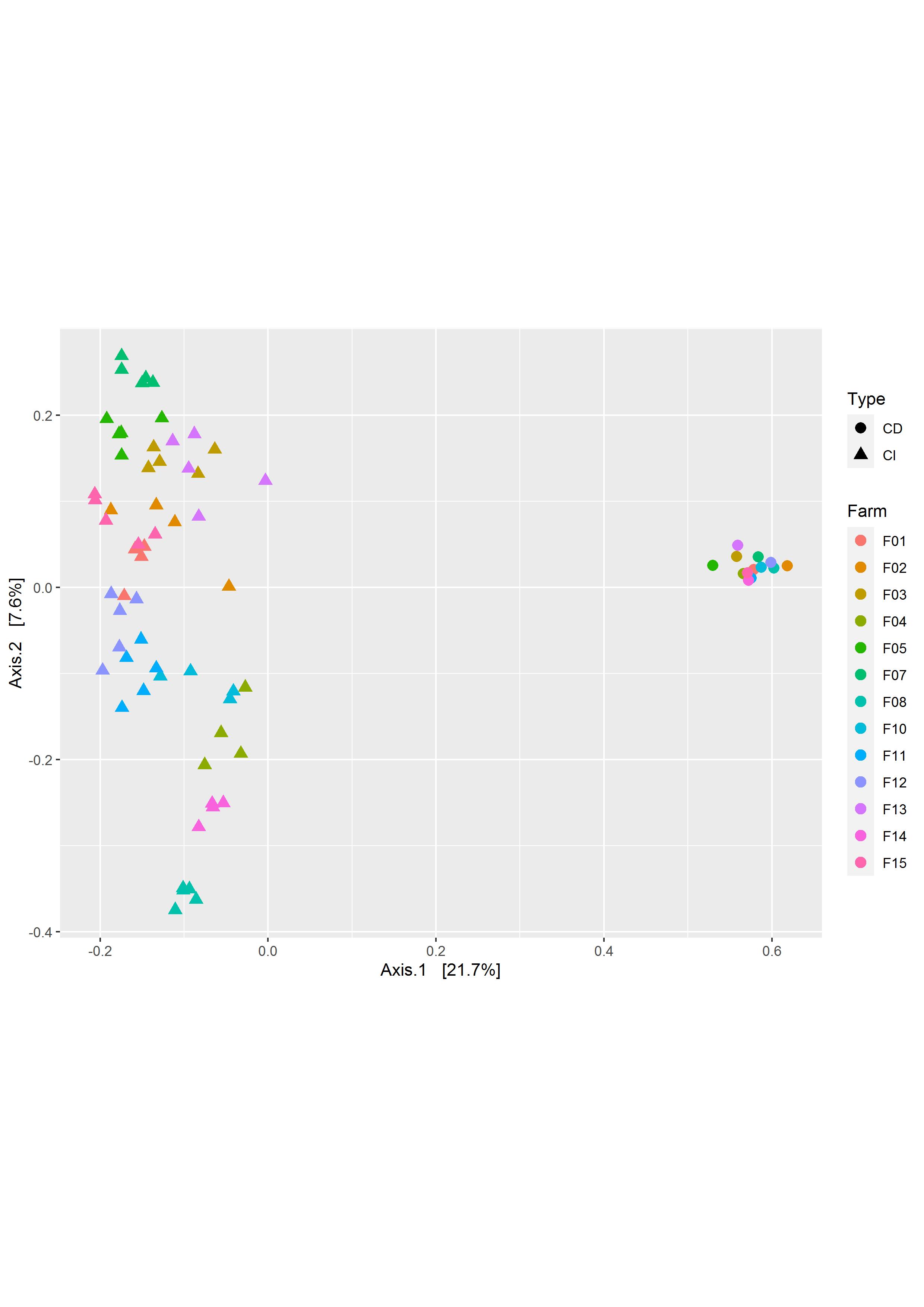


**Figure S9**: PCoA plot based on Bray-Curtis distance on presence-absence matrix of ASVs.

**
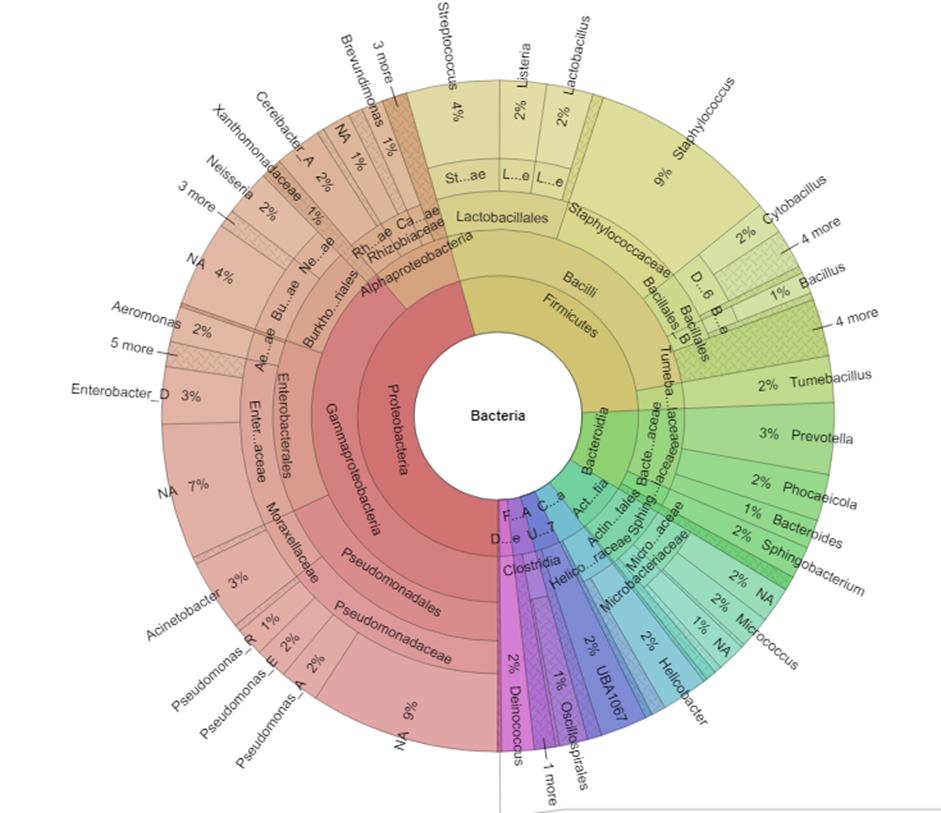
**

**Figure S10**: Snapshot of Krona plot representing Genus level diversity of ASVs exclusively detected in culture-dependent data.

**
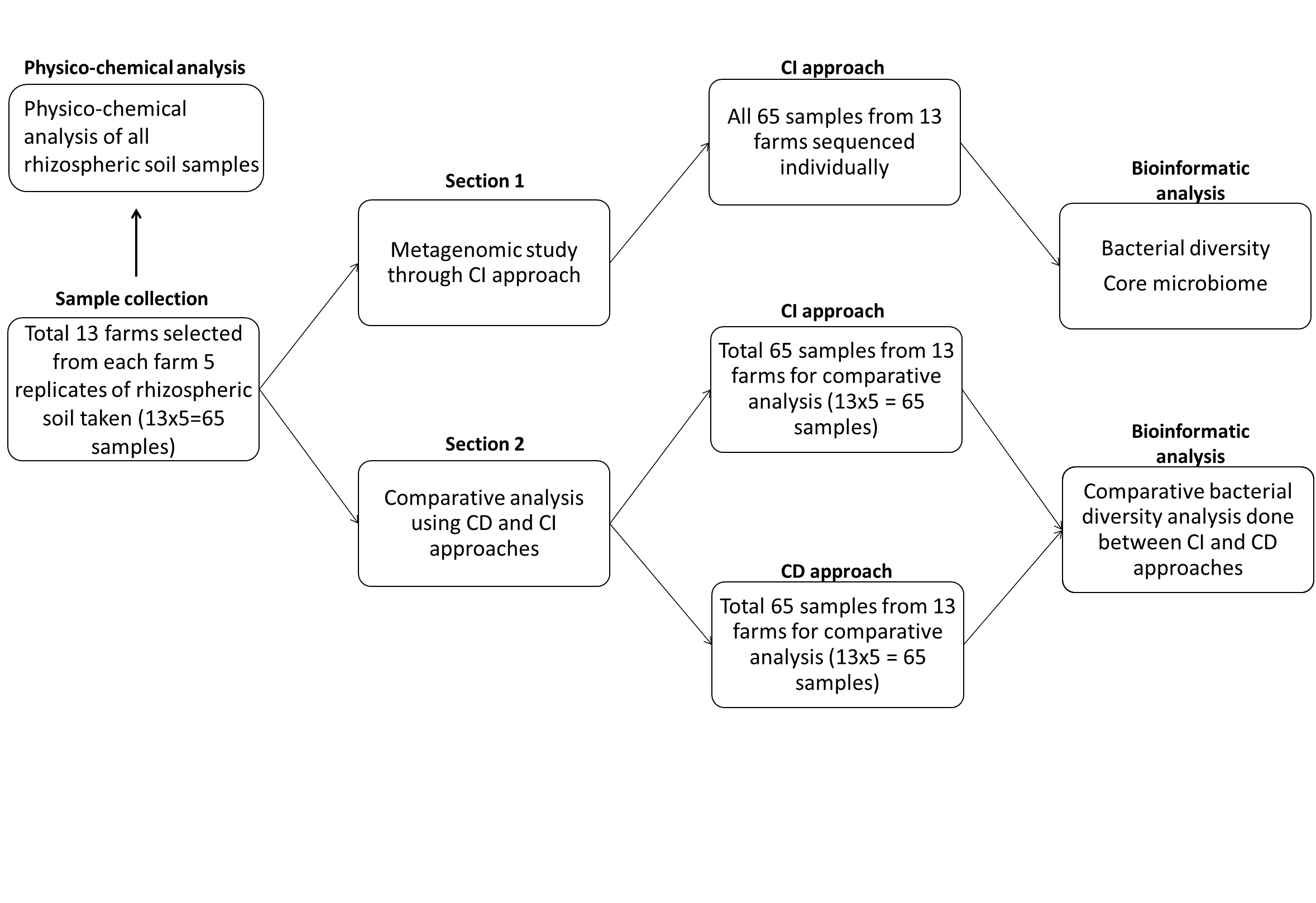
**

**Figure S11**: Schematic representation of samples used in different approaches.

**
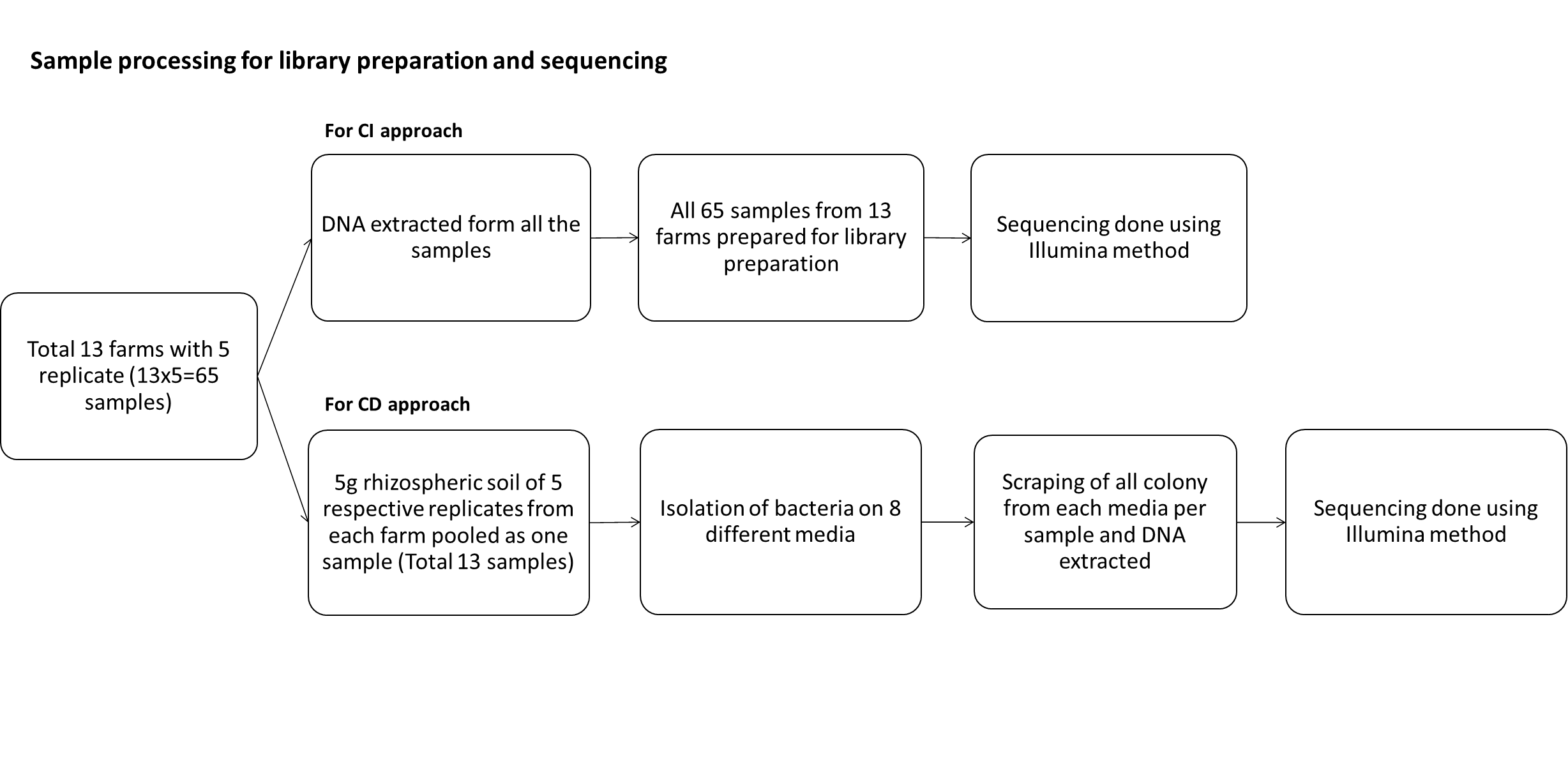
**

**Figure S12:** Schematic representation of library preparation and sequencing for both approaches. Using CD approach, bacterial isolation from 65 samples belonging to 13 farms (5 replicates from each farm) was done by pooling equal amount of rhizospheric soil from all 5 replicates of same farm as one sample, which empirically represents cultivable bacterial diversity of all replicates for that particular farm. As we did bacterial isolation on 8 different media using two different dilutions, and plates were incubated at two different temperatures in triplicates so, for each farm bacterial isolation was done on 96 plates (8 media x 2 dilutions x 2 temperatures x 3 replicates).

**Supplementary tables**

**Table S1**: Reads distribution across taxonomic levels for GTDB v202 database.

|  | GTDB v202 |
| --- | --- |
| Kingdom | 17717 |
| Phylum | 16153 |
| Class | 15733 |
| Order | 14819 |
| Family | 12688 |
| Genus | 8541 |
| Species | 126 |

**Table S2:** Kruskal-Wallis test comparison p-values of Phylum abundance of all farm samples. Phyla abundance values shown as mean & standard deviation of each farm. P-values < 0.05 are coloured as blue.

| Phyla | p-value | F01 | F02 | F03 | F04 | F05 | F07 | F08 | F10 | F11 | F12 | F13 | F14 | F15 |
| --- | --- | --- | --- | --- | --- | --- | --- | --- | --- | --- | --- | --- | --- | --- |
| Acidobacteriota | 0.002007 | 0.177362 ± 0.018569 | 0.135471 ± 0.010051 | 0.138443 ± 0.032389 | 0.200543 ± 0.050248 | 0.189374 ± 0.024045 | 0.148737 ± 0.01606 | 0.185046 ± 0.005471 | 0.202835 ± 0.016171 | 0.194277 ± 0.038881 | 0.156233 ± 0.030859 | 0.147982 ± 0.027008 | 0.148519 ± 0.037191 | 0.191999 ± 0.041182 |
| Actinobacteriota | 0.010894 | 0.121963 ± 0.017762 | 0.131179 ± 0.011517 | 0.1299 ± 0.048186 | 0.087896 ± 0.032039 | 0.109951 ± 0.02531 | 0.162386 ± 0.027742 | 0.092463 ± 0.014958 | 0.125789 ± 0.026256 | 0.095584 ± 0.023977 | 0.119112 ± 0.023174 | 0.110877 ± 0.034191 | 0.080166 ± 0.01340 | 0.117149 ± 0.022237 |
| Armatimonadota | 0.000484 | 0.00044 ± 0.000651 | 0.001506 ± 0.001207 | 0.002796 ± 0.001553 | 0.001488 ± 0.001163 | 0.005701 ± 0.002006 | 0.001365 ± 0.001477 | 0.002095 ± 0.001318 | 0.000158 ± 0.000316 | 0.003556 ± 0.00106 | 0.003574 ± 0.000773 | 0.004146 ± 0.00227 | 0.000943 ± 0.001636 | 0.001462 ± 0.000709 |
| Bacteroidota | 0.005639 | 0.034803 ± 0.005827 | 0.050234 ± 0.020363 | 0.109526 ± 0.082015 | 0.042015 ± 0.022128 | 0.027327 ± 0.005826 | 0.033338 ± 0.01161 | 0.021903 ± 0.004082 | 0.043373 ± 0.034589 | 0.038333 ± 0.020185 | 0.03245 ± 0.011159 | 0.086559 ± 0.030059 | 0.035595 ± 0.007241 | 0.024812 ± 0.011078 |
| Chloroflexota | 0.003779 | 0.011347 ± 0.003599 | 0.014042 ± 0.006944 | 0.020776 ± 0.00246 | 0.021002 ± 0.011932 | 0.02456 ± 0.005538 | 0.031025 ± 0.004342 | 0.031101 ± 0.009011 | 0.012445 ± 0.011465 | 0.021862 ± 0.004275 | 0.018799 ± 0.007336 | 0.017203 ± 0.023479 | 0.010053 ± 0.001892 | 0.019239 ± 0.005795 |
| Cyanobacteria | 0.620868 | 0 | 0 | 0.000778 ± 0.001403 | 0 | 0 | 0 | 0.000375 ± 0.000838 | 0.000115 ± 0.00023 | 0.000062 ± 0.000139 | 0.000133 ± 0.000297 | 0.000061 ± 0.000137 | 0 | 0 |
| Desulfobacterota_B | 0.000772 | 0.016465 ± 0.00184 | 0.008053 ± 0.00266 | 0.007223 ± 0.003103 | 0.012276 ± 0.00533 | 0.011359 ± 0.001978 | 0.009187 ± 0.003236 | 0.012362 ± 0.00164 | 0.013424 ± 0.001044 | 0.017557 ± 0.004189 | 0.013251 ± 0.004351 | 0.010362 ± 0.002947 | 0.009918 ± 0.002996 | 0.015762 ± 0.002308 |
| Eisenbacteria | 0.000041 | 0.000609 ± 0.000594 | 0.000902 ± 0.000968 | 0.000638 ± 0.000652 | 0.000452 ± 0.000904 | 0.004607 ± 0.002004 | 0.000452 ± 0.000705 | 0.003822 ± 0.001914 | 0.001565 ± 0.000698 | 0.004104 ± 0.001149 | 0.003182 ± 0.000382 | 0.000471 ± 0.00067 | 0 | 0.00259 ± 0.002048 |
| Firmicutes | 0.000347 | 0.043231 ± 0.006428 | 0.04591 ± 0.009367 | 0.040737 ± 0.015544 | 0.038349 ± 0.012284 | 0.021958 ± 0.005964 | 0.057552 ± 0.025815 | 0.018273 ± 0.003671 | 0.049267 ± 0.020565 | 0.024548 ± 0.018788 | 0.019814 ± 0.004854 | 0.044238 ± 0.018707 | 0.036266 ± 0.004832 | 0.056469 ± 0.012468 |
| Firmicutes_A | 0.123503 | 0 | 0 | 0.000141 ± 0.000315 | 0.001459 ± 0.002919 | 0.000464 ± 0.000606 | 0.000894 ± 0.001254 | 0.000621 ± 0.000966 | 0.000187 ± 0.000373 | 0.000166 ± 0.000235 | 0.000313 ± 0.000202 | 0 | 0 | 0.000046 ± 0.000091 |
| Gemmatimonadota | 0.000073 | 0.013541 ± 0.002979 | 0.011029 ± 0.001975 | 0.01311 ± 0.003476 | 0.01099 ± 0.004416 | 0.02953 ± 0.005336 | 0.030881 ± 0.006197 | 0.014287 ± 0.003347 | 0.013032 ± 0.003192 | 0.010723 ± 0.001782 | 0.015824 ± 0.004343 | 0.01205 ± 0.001863 | 0.005318 ± 0.002746 | 0.016618 ± 0.002389 |
| Hydrogenedentota | 0.278339 | 0 | 0 | 0.000141 ± 0.000315 | 0 | 0 | 0 | 0.000096 ± 0.000132 | 0 | 0.000098 ± 0.000167 | 0.001011 ± 0.00226 | 0 | 0 | 0 |
| Methylomirabilota | 0.003625 | 0.009414 ± 0.002499 | 0.004222 ± 0.003104 | 0.007884 ± 0.000705 | 0.00982 ± 0.007807 | 0.014971 ± 0.003036 | 0.008344 ± 0.003795 | 0.011569 ± 0.002002 | 0.009392 ± 0.006622 | 0.009498 ± 0.001865 | 0.008679 ± 0.002438 | 0.00424 ± 0.004137 | 0.002955 ± 0.002083 | 0.010201 ± 0.003176 |
| Myxococcota | 0.029450 | 0.015241 ± 0.002538 | 0.022787 ± 0.004955 | 0.01533 ± 0.005341 | 0.026038 ± 0.010989 | 0.015319 ± 0.002925 | 0.012856 ± 0.003035 | 0.015615 ± 0.004395 | 0.011906 ± 0.003448 | 0.013725 ± 0.001917 | 0.012238 ± 0.001404 | 0.018793 ± 0.004753 | 0.016227 ± 0.003835 | 0.014531 ± 0.004075 |
| Nitrospirota | 0.002659 | 0.008815 ± 0.00204 | 0.006825 ± 0.00116 | 0.010143 ± 0.003333 | 0.006833 ± 0.002119 | 0.005881 ± 0.001617 | 0.005224 ± 0.001067 | 0.006426 ± 0.001503 | 0.010858 ± 0.001547 | 0.005208 ± 0.001783 | 0.004865 ± 0.001654 | 0.005875 ± 0.001608 | 0.004818 ± 0.001272 | 0.007437 ± 0.000465 |
| Patescibacteria | 0.001659 | 0.016902 ± 0.011152 | 0.004972 ± 0.001291 | 0.010601 ± 0.005772 | 0.009029 ± 0.006367 | 0.005636 ± 0.004999 | 0.003358 ± 0.002161 | 0.040383 ± 0.01112 | 0.025002 ± 0.009692 | 0.013617 ± 0.010835 | 0.029798 ± 0.014278 | 0.003129 ± 0.004258 | 0.019653 ± 0.025795 | 0.026979 ± 0.034774 |
| Planctomycetota | 0.000004 | 0.129016 ± 0.004355 | 0.116797 ± 0.035117 | 0.104655 ± 0.026186 | 0.146169 ± 0.017648 | 0.160093 ± 0.020117 | 0.121751 ± 0.019965 | 0.210371 ± 0.017622 | 0.153842 ± 0.008775 | 0.177542 ± 0.023269 | 0.200437 ± 0.011607 | 0.110763 ± 0.012786 | 0.231414 ± 0.020716 | 0.163928 ± 0.029096 |
| Proteobacteria | 0.002455 | 0.340148 ± 0.019622 | 0.386256 ± 0.025895 | 0.330884 ± 0.036743 | 0.314054 ± 0.024212 | 0.293953 ± 0.023138 | 0.312907 ± 0.021454 | 0.255238 ± 0.022423 | 0.275052 ± 0.061406 | 0.300065 ± 0.062442 | 0.275  ± 0.049481 | 0.352317 ± 0.056039 | 0.308138 ± 0.037428 | 0.256826 ± 0.026068 |
| Unclassified Phylum | 0.000169 | 0.024057 ± 0.008171 | 0.014658 ± 0.003322 | 0.02367 ± 0.003493 | 0.029053 ± 0.011328 | 0.038447 ± 0.002356 | 0.029773 ± 0.00667 | 0.042837 ± 0.006625 | 0.023221 ± 0.009387 | 0.026463 ± 0.004292 | 0.039458 ± 0.010917 | 0.023005 ± 0.006461 | 0.017019 ± 0.003058 | 0.036011 ± 0.008285 |
| Verrucomicrobiota | 0.012363 | 0.036645 ± 0.008057 | 0.044394 ± 0.011413 | 0.032625 ± 0.00253 | 0.042384 ± 0.018029 | 0.040652 ± 0.012592 | 0.02972 ± 0.006584 | 0.032561 ± 0.00836 | 0.028273 ± 0.007639 | 0.042623 ± 0.009146 | 0.045229 ± 0.01658 | 0.047929 ± 0.008809 | 0.072486 ± 0.022958 | 0.037083 ± 0.021737 |

**Table S3:** Kruskal-Wallis test comparison p-values of Genus abundance of all farm samples. Genus abundance values shown as mean & standard deviation of each farm. “_X” are used to represent unannotated taxa at a level, multiple suffixes indicate that many level was the last annotation. P-values < 0.05 are coloured as blue.

| Genus | p-value | F01 | F02 | F03 | F04 | F05 | F07 | F08 | F10 | F11 | F12 | F13 | F14 | F15 |
| --- | --- | --- | --- | --- | --- | --- | --- | --- | --- | --- | --- | --- | --- | --- |
| Feb-22 | 0.484612 | 0 | 0.000313 ± 0.000626 | 0 | 0 | 0 | 0 | 0.000141 ± 0.000314 | 0 | 0 | 0 | 0 | 0 | 0 |
| 17J80-11 | 0.222123 | 0.001049 ± 0.000731 | 0.000303 ± 0.000607 | 0.001554 ± 0.002223 | 0.002099 ± 0.004199 | 0.001222 ± 0.000863 | 0.001200 ± 0.000744 | 0.001409 ± 0.000888 | 0 | 0.000536 ± 0.000744 | 0.000144 ± 0.000323 | 0.000315 ± 0.000705 | 0.000781 ± 0.001562 | 0.000397 ± 0.000503 |
| 2-01-FULL-44-21 | 0.037830 | 0 | 0 | 0 | 0 | 0 | 0 | 0 | 0 | 0.000200 ± 0.000276 | 0 | 0 | 0 | 0 |
| 2-12-FULL-64-23 | 0.009304 | 0.000447 ± 0.000894 | 0.000180 ± 0.000361 | 0 | 0 | 0.001873 ± 0.001444 | 0.000105 ± 0.000235 | 0.000508 ± 0.000718 | 0.002402 ± 0.002067 | 0.000844 ± 0.000624 | 0.000301 ± 0.000424 | 0.000156 ± 0.000348 | 0 | 0 |
| 2-12-FULL-66-21_X | 0.011364 | 0.012152 ± 0.005863 | 0.009355 ± 0.004148 | 0.009105 ± 0.003097 | 0.019624 ± 0.006326 | 0.015784 ± 0.003343 | 0.017310 ± 0.004152 | 0.017021 ± 0.005655 | 0.017921 ± 0.007801 | 0.013815 ± 0.002957 | 0.010649 ± 0.003016 | 0.005747 ± 0.002146 | 0.017148 ± 0.012761 | 0.012174 ± 0.008257 |
| 40CM-3-62-11 | 0.000533 | 0.000400 ± 0.000462 | 0.000078 ± 0.000157 | 0.001449 ± 0.000930 | 0 | 0.000883 ± 0.000503 | 0.000860 ± 0.000825 | 0 | 0 | 0.000411 ± 0.000566 | 0.000036 ± 0.000081 | 0 | 0 | 0.000763 ± 0.000215 |
| 40CM-68-15 | 0.002515 | 0.001116 ± 0.001138 | 0 | 0.000339 ± 0.000759 | 0 | 0.001534 ± 0.001204 | 0.000532 ± 0.000772 | 0.000962 ± 0.000946 | 0 | 0.000961 ± 0.000917 | 0.001654 ± 0.000572 | 0.000041 ± 0.000092 | 0 | 0.000034 ± 0.000068 |
| 4572-78_X_X | 0.546132 | 0 | 0 | 0 | 0 | 0 | 0 | 0.000095 ± 0.000211 | 0 | 0 | 0 | 0 | 0 | 0 |
| 55-13 | 0.091097 | 0.000344 ± 0.000689 | 0.000721 ± 0.000837 | 0.000307 ± 0.000424 | 0 | 0.000649 ± 0.000496 | 0.000223 ± 0.000499 | 0.000773 ± 0.000654 | 0 | 0.000551 ± 0.000170 | 0.000458 ± 0.000380 | 0.000654 ± 0.000604 | 0 | 0.000258 ± 0.000516 |
| A4b_X | 0.037830 | 0 | 0 | 0 | 0 | 0 | 0 | 0.000090 ± 0.000129 | 0 | 0 | 0 | 0 | 0 | 0 |
| Abditibacterium | 0.344682 | 0 | 0.000126 ± 0.000183 | 0 | 0.000215 ± 0.000430 | 0.000080 ± 0.000180 | 0 | 0 | 0 | 0 | 0.000024 ± 0.000053 | 0 | 0.000098 ± 0.000195 | 0 |
| AC-14 | 0.006759 | 0.004690 ± 0.001742 | 0.000724 ± 0.001448 | 0.001706 ± 0.001772 | 0.004685 ± 0.004308 | 0.004903 ± 0.002712 | 0.003998 ± 0.001180 | 0.001035 ± 0.001159 | 0.000373 ± 0.000746 | 0.002951 ± 0.000777 | 0.001314 ± 0.001539 | 0.002468 ± 0.002030 | 0.000652 ± 0.001305 | 0.002408 ± 0.001682 |
| AC-32 | 0.001783 | 0.000654 ± 0.000973 | 0.000088 ± 0.000176 | 0.001179 ± 0.000764 | 0 | 0.002413 ± 0.001355 | 0.000308 ± 0.000517 | 0.000954 ± 0.000697 | 0.006087 ± 0.004557 | 0.002200 ± 0.001153 | 0.002295 ± 0.000827 | 0.001697 ± 0.001583 | 0 | 0.002261 ± 0.001071 |
| AC-51 | 0.017678 | 0 | 0 | 0.000272 ± 0.000608 | 0.000502 ± 0.001005 | 0 | 0.000881 ± 0.000194 | 0.000213 ± 0.000291 | 0.002020 ± 0.002539 | 0.000197 ± 0.000281 | 0.000554 ± 0.000674 | 0 | 0 | 0.000434 ± 0.000534 |
| AC-67 | 0.045906 | 0 | 0 | 0.000170 ± 0.000379 | 0 | 0 | 0 | 0 | 0 | 0 | 0 | 0 | 0 | 0.000566 ± 0.000722 |
| AC-69 | 0.205006 | 0.002607 ± 0.002092 | 0.002710 ± 0.000594 | 0.000928 ± 0.001515 | 0.001833 ± 0.002174 | 0.002306 ± 0.001403 | 0.002990 ± 0.000780 | 0.002627 ± 0.001385 | 0.000861 ± 0.001722 | 0.001854 ± 0.001709 | 0.005413 ± 0.003242 | 0.001274 ± 0.001749 | 0.001042 ± 0.001254 | 0.002715 ± 0.001938 |
| Acetivibrio | 0.546132 | 0 | 0 | 0 | 0 | 0 | 0 | 0.000148 ± 0.000331 | 0 | 0 | 0 | 0 | 0 | 0 |
| Acetobacteraceae_X | 0.003992 | 0 | 0 | 0 | 0 | 0.000858 ± 0.000578 | 0 | 0.000237 ± 0.000325 | 0 | 0.000088 ± 0.000197 | 0.000443 ± 0.000803 | 0 | 0 | 0 |
| Acidimicrobiales_X_X | 0.011922 | 0.003143 ± 0.002505 | 0.002661 ± 0.002973 | 0.005881 ± 0.003390 | 0.001937 ± 0.001369 | 0.004868 ± 0.001387 | 0.001937 ± 0.001650 | 0.006303 ± 0.001293 | 0.003313 ± 0.002775 | 0.003856 ± 0.003164 | 0.008082 ± 0.002971 | 0.002648 ± 0.001948 | 0.001762 ± 0.002149 | 0.004885 ± 0.001661 |
| Acidimicrobiia_X_X_X | 0.000017 | 0 | 0 | 0 | 0.000624 ± 0.001248 | 0 | 0 | 0.002224 ± 0.000982 | 0.000445 ± 0.000890 | 0 | 0 | 0 | 0 | 0 |
| Acidisphaera | 0.789291 | 0 | 0.000342 ± 0.000685 | 0.000463 ± 0.001034 | 0 | 0 | 0.000240 ± 0.000536 | 0.000097 ± 0.000217 | 0 | 0.000303 ± 0.000678 | 0 | 0 | 0 | 0 |
| Acidobacteriales_X_X | 0.396957 | 0 | 0.000139 ± 0.000277 | 0.000157 ± 0.000351 | 0.000126 ± 0.000251 | 0.000227 ± 0.000324 | 0 | 0 | 0 | 0.000225 ± 0.000502 | 0.000288 ± 0.000266 | 0.000131 ± 0.000293 | 0 | 0 |
| Acidobacteriota_X_X_X_X | 0.008381 | 0.003830 ± 0.001531 | 0.004261 ± 0.003049 | 0.005288 ± 0.002359 | 0.005308 ± 0.004059 | 0.006896 ± 0.001414 | 0.004207 ± 0.001757 | 0.004866 ± 0.001865 | 0.010043 ± 0.002147 | 0.003559 ± 0.001488 | 0.007354 ± 0.002441 | 0.005129 ± 0.002371 | 0.000839 ± 0.001150 | 0.004659 ± 0.001871 |
| Acidobacterium_A | 0.005580 | 0 | 0 | 0 | 0.000607 ± 0.000741 | 0 | 0 | 0 | 0 | 0 | 0 | 0 | 0 | 0 |
| Acinetobacter | 0.003156 | 0.000213 ± 0.000427 | 0.002224 ± 0.001773 | 0.003736 ± 0.003340 | 0.003054 ± 0.005842 | 0.000325 ± 0.000393 | 0.005380 ± 0.004834 | 0.000025 ± 0.000057 | 0.003157 ± 0.006314 | 0.000197 ± 0.000316 | 0.000229 ± 0.000512 | 0.005262 ± 0.003822 | 0 | 0.000015 ± 0.000030 |
| Actinobacteriota_X_X_X_X | 0.000467 | 0.002749 ± 0.001864 | 0.001685 ± 0.001950 | 0.010885 ± 0.005546 | 0.000817 ± 0.001633 | 0.009249 ± 0.002353 | 0.011569 ± 0.003302 | 0.010034 ± 0.003792 | 0.015725 ± 0.011421 | 0.009752 ± 0.005156 | 0.012351 ± 0.004714 | 0.006084 ± 0.003946 | 0.000735 ± 0.001470 | 0.004399 ± 0.002868 |
| Actinocorallia | 0.618381 | 0 | 0 | 0 | 0 | 0 | 0 | 0.000107 ± 0.000238 | 0 | 0 | 0 | 0.000307 ± 0.000685 | 0 | 0 |
| Actinomycetales_X_X | 0.189236 | 0.000440 ± 0.000845 | 0.000492 ± 0.000912 | 0.000672 ± 0.001317 | 0.000682 ± 0.000872 | 0.000066 ± 0.000148 | 0.000424 ± 0.000490 | 0.000063 ± 0.000141 | 0 | 0.000302 ± 0.000676 | 0 | 0.001074 ± 0.001167 | 0.000285 ± 0.000569 | 0.000437 ± 0.000827 |
| Actinomycetia_X_X_X | 0.095425 | 0.000304 ± 0.000609 | 0.000305 ± 0.000610 | 0.002067 ± 0.001817 | 0.000264 ± 0.000528 | 0.000342 ± 0.000495 | 0 | 0.001896 ± 0.001581 | 0.000235 ± 0.000470 | 0.000464 ± 0.000445 | 0.000928 ± 0.000797 | 0.000874 ± 0.001953 | 0.000569 ± 0.001138 | 0.000330 ± 0.000660 |
| Actinophytocola | 0.000177 | 0 | 0 | 0 | 0 | 0 | 0.001602 ± 0.002174 | 0 | 0 | 0 | 0 | 0.000221 ± 0.000494 | 0 | 0 |
| Actinoplanes | 0.000382 | 0.004009 ± 0.002143 | 0.004968 ± 0.003541 | 0.001310 ± 0.001894 | 0.002001 ± 0.004001 | 0.003508 ± 0.001269 | 0.005209 ± 0.001989 | 0.001174 ± 0.001351 | 0.000548 ± 0.001096 | 0.003654 ± 0.001077 | 0.004019 ± 0.001086 | 0.011418 ± 0.007770 | 0.031610 ± 0.018887 | 0.003658 ± 0.004438 |
| Actinosynnema | 0.316957 | 0.000515 ± 0.001030 | 0 | 0 | 0 | 0 | 0 | 0 | 0 | 0 | 0 | 0 | 0 | 0 |
| Adhaeribacter | 0.003607 | 0.002585 ± 0.000909 | 0.000541 ± 0.001082 | 0 | 0 | 0.000253 ± 0.000566 | 0.000217 ± 0.000486 | 0 | 0 | 0 | 0.000098 ± 0.000219 | 0.000319 ± 0.000714 | 0 | 0.000305 ± 0.000352 |
| Aeromicrobium | 0.546132 | 0 | 0 | 0 | 0 | 0 | 0 | 0 | 0 | 0 | 0.000707 ± 0.001582 | 0 | 0 | 0 |
| Aeromonas | 0.306898 | 0 | 0.001298 ± 0.002596 | 0 | 0 | 0.000046 ± 0.000103 | 0 | 0 | 0.000569 ± 0.000753 | 0.000155 ± 0.000347 | 0.001389 ± 0.002232 | 0 | 0.000819 ± 0.001114 | 0.002175 ± 0.004350 |
| Afipia | 0.078179 | 0.004913 ± 0.001610 | 0.003274 ± 0.002441 | 0.003252 ± 0.002275 | 0.002439 ± 0.001779 | 0.003398 ± 0.000904 | 0.000965 ± 0.001321 | 0.003478 ± 0.001302 | 0.004760 ± 0.003462 | 0.003899 ± 0.000653 | 0.001959 ± 0.001157 | 0.002532 ± 0.002361 | 0.003112 ± 0.002980 | 0.001873 ± 0.001266 |
| Agrobacterium | 0.063117 | 0.000526 ± 0.000686 | 0.003351 ± 0.002532 | 0.001994 ± 0.002426 | 0.000723 ± 0.001031 | 0.002117 ± 0.001587 | 0.000926 ± 0.000554 | 0.001091 ± 0.001176 | 0 | 0.002102 ± 0.002398 | 0.002971 ± 0.003219 | 0.007464 ± 0.005525 | 0.000550 ± 0.000701 | 0.000228 ± 0.000457 |
| Agromyces | 0.140458 | 0.001397 ± 0.001201 | 0.000984 ± 0.001142 | 0 | 0 | 0.000414 ± 0.000927 | 0.000794 ± 0.000597 | 0.001202 ± 0.000491 | 0.001603 ± 0.001292 | 0.001293 ± 0.000752 | 0.000896 ± 0.000835 | 0.000715 ± 0.001010 | 0.001048 ± 0.002095 | 0.000867 ± 0.000755 |
| Alicyclobacillia_X_X_X | 0.316957 | 0 | 0 | 0 | 0 | 0 | 0 | 0 | 0 | 0 | 0 | 0 | 0.000358 ± 0.000717 | 0 |
| Alienimonas | 0.208587 | 0.001885 ± 0.001439 | 0.000711 ± 0.000686 | 0.000104 ± 0.000232 | 0.000565 ± 0.001131 | 0.000285 ± 0.000447 | 0.000230 ± 0.000515 | 0.000665 ± 0.000645 | 0 | 0.000373 ± 0.000347 | 0.000254 ± 0.000349 | 0.000414 ± 0.000925 | 0.000156 ± 0.000312 | 0.001081 ± 0.001034 |
| Allosphingosinicella | 0.054132 | 0.000312 ± 0.000624 | 0 | 0.000039 ± 0.000086 | 0 | 0 | 0.000792 ± 0.001306 | 0 | 0 | 0.000406 ± 0.000416 | 0 | 0 | 0 | 0 |
| AL-N1 | 0.020774 | 0.000296 ± 0.000591 | 0 | 0.000208 ± 0.000465 | 0 | 0.001124 ± 0.000680 | 0 | 0.000097 ± 0.000217 | 0 | 0.000159 ± 0.000355 | 0.000380 ± 0.000527 | 0 | 0 | 0.000618 ± 0.000714 |
| ALPHA2B | 0.000824 | 0 | 0 | 0 | 0 | 0.000741 ± 0.001066 | 0 | 0.000267 ± 0.000596 | 0.000588 ± 0.001177 | 0.002603 ± 0.002214 | 0.001148 ± 0.001206 | 0 | 0 | 0 |
| Alphaproteobacteria_X_X_X | 0.051742 | 0.012483 ± 0.004145 | 0.016688 ± 0.005419 | 0.015855 ± 0.003898 | 0.005417 ± 0.004885 | 0.014950 ± 0.002250 | 0.016715 ± 0.004016 | 0.012396 ± 0.002174 | 0.014599 ± 0.009614 | 0.011795 ± 0.002117 | 0.011874 ± 0.004458 | 0.010736 ± 0.004437 | 0.014597 ± 0.007841 | 0.012083 ± 0.001809 |
| Amycolatopsis | 0.546132 | 0 | 0 | 0 | 0 | 0 | 0 | 0.000395 ± 0.000884 | 0 | 0 | 0 | 0 | 0 | 0 |
| Anaerolineae_X_X_X | 0.035873 | 0.000394 ± 0.000788 | 0 | 0.000242 ± 0.000540 | 0.000962 ± 0.001327 | 0 | 0 | 0.001688 ± 0.001048 | 0.000301 ± 0.000603 | 0 | 0.000389 ± 0.000589 | 0 | 0.000684 ± 0.000884 | 0.000306 ± 0.000612 |
| Anaerolineales_X_X | 0.217684 | 0 | 0 | 0 | 0 | 0.000121 ± 0.000176 | 0 | 0 | 0 | 0.000028 ± 0.000062 | 0 | 0 | 0 | 0.000061 ± 0.000121 |
| Anaeromyxobacter | 0.037830 | 0 | 0 | 0 | 0 | 0 | 0 | 0.000223 ± 0.000306 | 0 | 0 | 0 | 0 | 0 | 0 |
| Anaeromyxobacteraceae_X | 0.037830 | 0 | 0 | 0 | 0 | 0 | 0 | 0.000145 ± 0.0002 | 0 | 0 | 0 | 0 | 0 | 0 |
| Anammoximicrobium | 0.000807 | 0 | 0 | 0 | 0.000226 ± 0.000452 | 0 | 0 | 0.00087 ± 0.000632 | 0 | 0 | 0.000076 ± 0.000171 | 0 | 0 | 0 |
| Aneurinibacillus_A | 0.000021 | 0 | 0.001052 ± 0.000737 | 0 | 0.003175 ± 0.000670 | 0 | 0 | 0 | 0 | 0 | 0 | 0 | 0.000314 ± 0.000629 | 0.000381 ± 0.000761 |
| Aquabacterium | 0.316957 | 0 | 0 | 0 | 0 | 0 | 0 | 0 | 0 | 0 | 0 | 0 | 0.000294 ± 0.000588 | 0 |
| Aquabacterium_A | 0.037830 | 0 | 0 | 0 | 0 | 0 | 0 | 0.000193 ± 0.000266 | 0 | 0 | 0 | 0 | 0 | 0 |
| Aquicella_A | 0.546132 | 0 | 0 | 0 | 0 | 0.000303 ± 0.000678 | 0 | 0 | 0 | 0 | 0 | 0 | 0 | 0 |
| Aquincola | 0.484612 | 0 | 0 | 0 | 0 | 0 | 0 | 0 | 0 | 0 | 0.000454 ± 0.001016 | 0 | 0.000570 ± 0.001140 | 0 |
| AR19 | 0.029462 | 0 | 0.002773 ± 0.002227 | 0.005049 ± 0.003277 | 0.001058 ± 0.001245 | 0.002628 ± 0.001520 | 0.000356 ± 0.000797 | 0.000261 ± 0.000583 | 0.000795 ± 0.001589 | 0.001719 ± 0.001097 | 0.001199 ± 0.000825 | 0.001094 ± 0.002446 | 0.000175 ± 0.000349 | 0.001227 ± 0.001614 |
| AR5 | 0.000277 | 0.001294 ± 0.000903 | 0 | 0.000242 ± 0.000540 | 0.001939 ± 0.001699 | 0.001018 ± 0.000300 | 0.000546 ± 0.000644 | 0.002353 ± 0.000689 | 0.001494 ± 0.0011 | 0.001281 ± 0.000715 | 0.000182 ± 0.000406 | 0 | 0 | 0.000306 ± 0.000364 |
| Arboricoccus | 0.000170 | 0.006273 ± 0.002875 | 0.000713 ± 0.000994 | 0.003109 ± 0.001481 | 0.001336 ± 0.001644 | 0.003400 ± 0.000822 | 0.002101 ± 0.001556 | 0.005457 ± 0.000953 | 0.001139 ± 0.001507 | 0.003019 ± 0.001996 | 0.006407 ± 0.002017 | 0.001385 ± 0.001419 | 0 | 0.005230 ± 0.003267 |
| Arenimonas | 0.091675 | 0.000367 ± 0.000429 | 0.001399 ± 0.000801 | 0.000171 ± 0.000383 | 0.00043 ± 0.00086 | 0.000516 ± 0.000472 | 0.000189 ± 0.000259 | 0.000076 ± 0.00017 | 0 | 0.000542 ± 0.000361 | 0.000681 ± 0.001038 | 0.000131 ± 0.000292 | 0.001259 ± 0.002518 | 0.000317 ± 0.000634 |
| Armatimonadaceae_X | 0.041110 | 0 | 0.000139 ± 0.000277 | 0.000243 ± 0.000334 | 0 | 0 | 0 | 0.000233 ± 0.000309 | 0 | 0.000289 ± 0.000275 | 0.000066 ± 0.000148 | 0 | 0 | 0 |
| Aromatoleum | 0.695527 | 0 | 0 | 0.000154 ± 0.000345 | 0 | 0.000221 ± 0.000494 | 0 | 0.000164 ± 0.000227 | 0 | 0.000220 ± 0.000493 | 0.000253 ± 0.000565 | 0 | 0 | 0.000406 ± 0.000812 |
| Arsenicitalea | 0.061985 | 0.000443 ± 0.000886 | 0 | 0 | 0 | 0.000517 ± 0.000713 | 0.001575 ± 0.001454 | 0.000279 ± 0.000411 | 0 | 0 | 0 | 0 | 0 | 0.000278 ± 0.000556 |
| Arthrobacter_B | 0.000057 | 0.004231 ± 0.001157 | 0.005669 ± 0.001890 | 0.000633 ± 0.001415 | 0.003031 ± 0.003499 | 0.003558 ± 0.001880 | 0.006519 ± 0.002838 | 0.000200 ± 0.000325 | 0 | 0 | 0.000380 ± 0.000360 | 0.000805 ± 0.001799 | 0.001892 ± 0.002387 | 0.006857 ± 0.002116 |
| Aureimonas_A | 0.324962 | 0.028709 ± 0.016423 | 0.028619 ± 0.020083 | 0.016362 ± 0.029381 | 0.010242 ± 0.009432 | 0.002497 ± 0.001658 | 0.010166 ± 0.008690 | 0.006284 ± 0.005721 | 0.022097 ± 0.028603 | 0.014807 ± 0.016071 | 0.020348 ± 0.040029 | 0.005626 ± 0.007646 | 0.039016 ± 0.034814 | 0.019604 ± 0.024824 |
| Azoarcus | 0.205613 | 0.001159 ± 0.001661 | 0.000513 ± 0.001026 | 0.000320 ± 0.000716 | 0 | 0.000136 ± 0.000304 | 0 | 0.000105 ± 0.000236 | 0 | 0.000045 ± 0.000100 | 0.000467 ± 0.000431 | 0 | 0 | 0 |
| Azoarcus_D | 0.130613 | 0 | 0 | 0 | 0 | 0 | 0 | 0.000123 ± 0.000183 | 0 | 0 | 0.000029 ± 0.000065 | 0 | 0 | 0 |
| Azohydromonas | 0.032219 | 0.001496 ± 0.002992 | 0.003403 ± 0.003955 | 0.006790 ± 0.004678 | 0.000703 ± 0.001407 | 0.003525 ± 0.001949 | 0.002684 ± 0.002498 | 0.004804 ± 0.004069 | 0 | 0.004144 ± 0.002376 | 0.003050 ± 0.001098 | 0.004724 ± 0.004528 | 0 | 0.001678 ± 0.002148 |
| Azospirillaceae_X | 0.005300 | 0.001015 ± 0.001178 | 0.001051 ± 0.001244 | 0.001800 ± 0.001102 | 0 | 0.000922 ± 0.000649 | 0.003076 ± 0.001028 | 0.001414 ± 0.000548 | 0 | 0.000696 ± 0.000733 | 0.001350 ± 0.000891 | 0.002211 ± 0.002123 | 0 | 0.001998 ± 0.000650 |
| Azospirillum | 0.054565 | 0.000677 ± 0.000858 | 0.000687 ± 0.000898 | 0.000285 ± 0.000638 | 0 | 0.001421 ± 0.000421 | 0.000813 ± 0.000836 | 0.000605 ± 0.000834 | 0.000648 ± 0.000809 | 0.000145 ± 0.000324 | 0.000973 ± 0.000575 | 0.000942 ± 0.000875 | 0 | 0.001683 ± 0.001175 |
| Azotobacter | 0.000705 | 0.000722 ± 0.001444 | 0.000757 ± 0.000900 | 0 | 0.002187 ± 0.002726 | 0.000922 ± 0.000616 | 0.002040 ± 0.002074 | 0.001768 ± 0.001467 | 0 | 0 | 0.003767 ± 0.000944 | 0 | 0.000648 ± 0.001297 | 0.004937 ± 0.003637 |
| Bacillaceae_H_X | 0.467767 | 0 | 0.000055 ± 0.000111 | 0.000191 ± 0.000269 | 0 | 0.000113 ± 0.000252 | 0.000168 ± 0.000249 | 0 | 0.000397 ± 0.000795 | 0.000175 ± 0.000373 | 0 | 0.000398 ± 0.000869 | 0 | 0 |
| Bacillales_B_X_X | 0.000757 | 0.009652 ± 0.000502 | 0.008822 ± 0.002532 | 0.006855 ± 0.002206 | 0.008878 ± 0.008535 | 0.004710 ± 0.001600 | 0.007585 ± 0.003206 | 0.003644 ± 0.001909 | 0.010759 ± 0.003794 | 0.003373 ± 0.002232 | 0.004105 ± 0.001105 | 0.012588 ± 0.006423 | 0.003724 ± 0.004301 | 0.010024 ± 0.002697 |
| Bacillales_X_X | 0.000567 | 0.003213 ± 0.000255 | 0.004949 ± 0.000464 | 0.003951 ± 0.000904 | 0.002763 ± 0.002554 | 0.001539 ± 0.000267 | 0.005434 ± 0.004111 | 0.001650 ± 0.001153 | 0.004247 ± 0.000687 | 0.001728 ± 0.001065 | 0.001504 ± 0.000940 | 0.003574 ± 0.002438 | 0.001416 ± 0.001637 | 0.004867 ± 0.001060 |
| Bacilli_X_X_X | 0.006234 | 0.014922 ± 0.003338 | 0.013054 ± 0.006457 | 0.010855 ± 0.006398 | 0.010169 ± 0.007695 | 0.005990 ± 0.002212 | 0.017066 ± 0.008806 | 0.004104 ± 0.002115 | 0.009370 ± 0.005974 | 0.008187 ± 0.006131 | 0.004426 ± 0.001817 | 0.011549 ± 0.003954 | 0.017524 ± 0.010726 | 0.021384 ± 0.006627 |
| Bacillus_AG | 0.625407 | 0 | 0.000310 ± 0.000620 | 0 | 0 | 0 | 0 | 0 | 0.000187 ± 0.000373 | 0.000136 ± 0.000304 | 0 | 0 | 0.000211 ± 0.000423 | 0.000011 ± 0.000023 |
| Bacillus_BD | 0.006349 | 0.004835 ± 0.002982 | 0.001793 ± 0.002000 | 0.000707 ± 0.000778 | 0.003893 ± 0.003061 | 0.001089 ± 0.001340 | 0.002790 ± 0.003208 | 0.000611 ± 0.000623 | 0.008426 ± 0.005603 | 0.004297 ± 0.004425 | 0.001580 ± 0.000624 | 0.002411 ± 0.001410 | 0.001705 ± 0.001348 | 0.004759 ± 0.001290 |
| Bacillus_BN | 0.748979 | 0.000299 ± 0.000409 | 0 | 0.000257 ± 0.000533 | 0 | 0.000211 ± 0.000471 | 0.000203 ± 0.000302 | 0.000012 ± 0.000027 | 0.000352 ± 0.000513 | 0.000079 ± 0.000176 | 0.000231 ± 0.000371 | 0.000469 ± 0.000657 | 0.000766 ± 0.001533 | 0 |
| Bacillus_BU | 0.692967 | 0 | 0 | 0.000322 ± 0.000720 | 0 | 0.000247 ± 0.000553 | 0 | 0 | 0 | 0.000297 ± 0.000665 | 0 | 0 | 0 | 0 |
| Bacteria_X_X_X_X_X | 0.000169 | 0.024057 ± 0.008171 | 0.014658 ± 0.003322 | 0.023670 ± 0.003493 | 0.029053 ± 0.011328 | 0.038447 ± 0.002356 | 0.029773 ± 0.006670 | 0.042837 ± 0.006625 | 0.023221 ± 0.009387 | 0.026463 ± 0.004292 | 0.039458 ± 0.010917 | 0.023005 ± 0.006461 | 0.017019 ± 0.003058 | 0.036011 ± 0.008285 |
| Bacteriovoracaceae_X | 0.316957 | 0 | 0 | 0 | 0 | 0 | 0 | 0 | 0 | 0 | 0 | 0 | 0.000358 ± 0.000717 | 0 |
| Bacteroidia_X_X_X | 0.000876 | 0 | 0.000244 ± 0.000284 | 0.000196 ± 0.000439 | 0 | 0.000285 ± 0.000397 | 0 | 0.000296 ± 0.000307 | 0.000665 ± 0.001329 | 0.001253 ± 0.000357 | 0.001483 ± 0.000418 | 0.000284 ± 0.000634 | 0.000074 ± 0.000147 | 0.000425 ± 0.000329 |
| Beijerinckiaceae_X | 0.060205 | 0.000394 ± 0.000787 | 0.000735 ± 0.000951 | 0.000108 ± 0.000241 | 0 | 0.000536 ± 0.000306 | 0.001683 ± 0.000708 | 0.000376 ± 0.000508 | 0.000372 ± 0.000744 | 0.000498 ± 0.000652 | 0.000434 ± 0.000558 | 0.001 ± 0.001412 | 0 | 0.000845 ± 0.000727 |
| Berkiella | 0.037830 | 0 | 0 | 0 | 0 | 0 | 0 | 0.000158 ± 0.000232 | 0 | 0 | 0 | 0 | 0 | 0 |
| Bin18 | 0.189806 | 0.000505 ± 0.000364 | 0.000263 ± 0.000527 | 0.000476 ± 0.000654 | 0 | 0.000640 ± 0.000587 | 0.000316 ± 0.000352 | 0.000672 ± 0.000441 | 0.000158 ± 0.000316 | 0.000788 ± 0.000450 | 0.000775 ± 0.000599 | 0.000574 ± 0.000559 | 0.000275 ± 0.000550 | 0.001303 ± 0.000734 |
| Bin18_X | 0.000140 | 0.000624 ± 0.000897 | 0.000194 ± 0.000388 | 0.000622 ± 0.000631 | 0 | 0.001554 ± 0.000139 | 0.001207 ± 0.000776 | 0.000783 ± 0.000540 | 0 | 0 | 0.001658 ± 0.000874 | 0.001482 ± 0.001403 | 0 | 0.001985 ± 0.000296 |
| BJ17 | 0.625502 | 0 | 0 | 0 | 0 | 0.000079 ± 0.000177 | 0 | 0 | 0.0001 ± 0.000201 | 0.000072 ± 0.00016 | 0 | 0 | 0 | 0.000152 ± 0.000304 |
| BJ22 | 0.546132 | 0 | 0 | 0 | 0 | 0 | 0 | 0 | 0 | 0.000213 ± 0.000477 | 0 | 0 | 0 | 0 |
| BJGV01 | 0.150299 | 0 | 0 | 0.000148 ± 0.000220 | 0 | 0 | 0 | 0 | 0 | 0 | 0 | 0.000098 ± 0.000220 | 0 | 0 |
| BJHL01 | 0.618381 | 0 | 0 | 0 | 0 | 0.000145 ± 0.000323 | 0 | 0.000055 ± 0.000124 | 0 | 0 | 0 | 0 | 0 | 0 |
| BJHM01 | 0.490977 | 0.000226 ± 0.000452 | 0 | 0 | 0 | 0.000139 ± 0.000190 | 0 | 0.000068 ± 0.000152 | 0.000691 ± 0.001383 | 0 | 0.000076 ± 0.000171 | 0 | 0 | 0 |
| BJHT01 | 0.175345 | 0.001346 ± 0.002691 | 0.001807 ± 0.002134 | 0.002515 ± 0.003147 | 0.003734 ± 0.005065 | 0.002826 ± 0.002491 | 0.001344 ± 0.001296 | 0.004650 ± 0.001407 | 0 | 0.002662 ± 0.001421 | 0.002630 ± 0.001832 | 0.002628 ± 0.003184 | 0.001561 ± 0.001827 | 0.002773 ± 0.001355 |
| Blastocatellia_X_X_X | 0.006994 | 0.000461 ± 0.000313 | 0.000370 ± 0.000429 | 0.000233 ± 0.000373 | 0.000628 ± 0.001256 | 0.000281 ± 0.000295 | 0.000226 ± 0.000414 | 0.002062 ± 0.000869 | 0.000474 ± 0.000947 | 0.001335 ± 0.000341 | 0.000367 ± 0.000453 | 0.000201 ± 0.000450 | 0.002966 ± 0.002640 | 0.001111 ± 0.000971 |
| Blastococcus | 0.003958 | 0.000365 ± 0.000731 | 0 | 0.000386 ± 0.000864 | 0 | 0.000450 ± 0.000629 | 0.003415 ± 0.001000 | 0 | 0 | 0.000486 ± 0.000673 | 0.000151 ± 0.000337 | 0.000966 ± 0.001376 | 0 | 0.000533 ± 0.001066 |
| BOG-1338 | 0.129225 | 0.000492 ± 0.000716 | 0 | 0.000141 ± 0.000315 | 0 | 0.000461 ± 0.000701 | 0.000116 ± 0.000260 | 0.000433 ± 0.000736 | 0 | 0.000226 ± 0.000506 | 0 | 0 | 0 | 0.000586 ± 0.000718 |
| Bog-159 | 0.789291 | 0 | 0 | 0 | 0 | 0.000224 ± 0.000501 | 0 | 0.000131 ± 0.000293 | 0 | 0.000142 ± 0.000318 | 0 | 0.000215 ± 0.000480 | 0 | 0.000205 ± 0.000410 |
| Bordetella_B | 0.510019 | 0.000731 ± 0.001461 | 0 | 0 | 0 | 0 | 0 | 0 | 0 | 0 | 0 | 0.000192 ± 0.000428 | 0.001691 ± 0.003381 | 0 |
| Bordetella_C | 0.316957 | 0 | 0 | 0 | 0 | 0 | 0 | 0 | 0 | 0 | 0 | 0 | 0.000239 ± 0.000478 | 0 |
| Bosea | 0.359367 | 0.000279 ± 0.000558 | 0.000842 ± 0.001014 | 0.000123 ± 0.000276 | 0 | 0.000586 ± 0.000842 | 0.000324 ± 0.000454 | 0.000163 ± 0.000274 | 0 | 0.000323 ± 0.000499 | 0.000957 ± 0.000606 | 0.000309 ± 0.000691 | 0.000211 ± 0.000423 | 0.000090 ± 0.000179 |
| Bradyrhizobium | 0.143235 | 0.001535 ± 0.001777 | 0 | 0.000214 ± 0.000479 | 0 | 0 | 0.001467 ± 0.001510 | 0 | 0.001090 ± 0.002180 | 0.003581 ± 0.006874 | 0.007470 ± 0.014101 | 0.001995 ± 0.003558 | 0 | 0.000759 ± 0.001518 |
| Brevibacillus_B | 0.188185 | 0 | 0 | 0 | 0.000151 ± 0.000301 | 0.000272 ± 0.000271 | 0.000293 ± 0.000520 | 0 | 0 | 0 | 0.000232 ± 0.000318 | 0.000172 ± 0.000384 | 0 | 0.000121 ± 0.000243 |
| Brevundimonas | 0.437952 | 0 | 0.000176 ± 0.000352 | 0 | 0.000535 ± 0.000773 | 0.000033 ± 0.000074 | 0.000217 ± 0.000298 | 0.000041 ± 0.000092 | 0 | 0 | 0.000109 ± 0.000243 | 0 | 0.001507 ± 0.003014 | 0.000156 ± 0.000182 |
| Bryobacteraceae_X | 0.004908 | 0.003913 ± 0.003210 | 0.002644 ± 0.003109 | 0.001607 ± 0.001050 | 0.004584 ± 0.003084 | 0.005487 ± 0.001185 | 0.002586 ± 0.001972 | 0.007834 ± 0.000857 | 0.002580 ± 0.003049 | 0.004937 ± 0.000532 | 0.007248 ± 0.002712 | 0.003699 ± 0.002620 | 0.002232 ± 0.002475 | 0.004875 ± 0.000654 |
| Burkholderia | 0.006225 | 0 | 0 | 0 | 0 | 0.000130 ± 0.000290 | 0.000371 ± 0.000563 | 0 | 0 | 0.000320 ± 0.000450 | 0.000872 ± 0.000778 | 0 | 0 | 0 |
| Burkholderiaceae_X | 0.487527 | 0.017353 ± 0.004181 | 0.016175 ± 0.006723 | 0.016962 ± 0.007478 | 0.016226 ± 0.005875 | 0.016124 ± 0.005509 | 0.014934 ± 0.006484 | 0.013800 ± 0.003717 | 0.017007 ± 0.005000 | 0.017687 ± 0.002334 | 0.018774 ± 0.008740 | 0.027674 ± 0.006048 | 0.017279 ± 0.009183 | 0.015692 ± 0.012898 |
| Burkholderiales_X_X | 0.014629 | 0.002367 ± 0.000938 | 0.000656 ± 0.001056 | 0.001091 ± 0.001032 | 0.003421 ± 0.001024 | 0.001609 ± 0.001428 | 0.001321 ± 0.001012 | 0.002675 ± 0.000410 | 0.003344 ± 0.001327 | 0.003143 ± 0.000937 | 0.001865 ± 0.001225 | 0.003320 ± 0.002068 | 0.001807 ± 0.001323 | 0.003196 ± 0.000933 |
| Byssovorax | 0.316957 | 0 | 0 | 0 | 0.000547 ± 0.001094 | 0 | 0 | 0 | 0 | 0 | 0 | 0 | 0 | 0 |
| CAADGA01 | 0.016657 | 0.000473 ± 0.000362 | 0 | 0 | 0.000063 ± 0.000126 | 0.000277 ± 0.000266 | 0.000051 ± 0.000113 | 0.001065 ± 0.001125 | 0 | 0.000516 ± 0.000493 | 0.000139 ± 0.000205 | 0 | 0.000752 ± 0.000970 | 0.000310 ± 0.000214 |
| CAADGG01 | 0.205588 | 0 | 0 | 0 | 0.000063 ± 0.000126 | 0 | 0.000388 ± 0.000867 | 0.000171 ± 0.000382 | 0 | 0 | 0 | 0 | 0.000764 ± 0.001112 | 0 |
| CAADGL01 | 0.000800 | 0 | 0 | 0 | 0 | 0 | 0 | 0.000355 ± 0.000377 | 0 | 0 | 0 | 0 | 0 | 0 |
| CABHOJ01 | 0.395571 | 0 | 0.000044 ± 0.000089 | 0.000369 ± 0.000551 | 0 | 0 | 0.000076 ± 0.000170 | 0 | 0 | 0.000188 ± 0.000302 | 0.000165 ± 0.000239 | 0.000981 ± 0.001353 | 0 | 0.000072 ± 0.000143 |
| CACIUL01 | 0.000800 | 0 | 0 | 0 | 0 | 0 | 0 | 0.000445 ± 0.000510 | 0 | 0 | 0 | 0 | 0 | 0 |
| CADCTF01 | 0.050517 | 0 | 0.000443 ± 0.000885 | 0 | 0.000201 ± 0.000402 | 0.000672 ± 0.000539 | 0.000243 ± 0.000333 | 0.000102 ± 0.000227 | 0 | 0.000338 ± 0.000204 | 0.000076 ± 0.000171 | 0 | 0.000393 ± 0.000786 | 0.000314 ± 0.000246 |
| CADCTO01 | 0.484612 | 0 | 0 | 0 | 0 | 0 | 0 | 0 | 0.000158 ± 0.000316 | 0.000071 ± 0.000159 | 0 | 0 | 0 | 0 |
| CADCWC01 | 0.150299 | 0 | 0 | 0.000160 ± 0.000358 | 0 | 0 | 0 | 0 | 0 | 0.000129 ± 0.000177 | 0 | 0 | 0 | 0 |
| CADCWI01 | 0.000888 | 0 | 0 | 0 | 0 | 0 | 0 | 0 | 0 | 0.000303 ± 0.000482 | 0.000990 ± 0.000737 | 0 | 0 | 0.000137 ± 0.000273 |
| CADCWK01 | 0.546132 | 0 | 0 | 0 | 0 | 0.000308 ± 0.000690 | 0 | 0 | 0 | 0 | 0 | 0 | 0 | 0 |
| CADCWL01 | 0.000032 | 0.001434 ± 0.000984 | 0.003232 ± 0.002345 | 0.004921 ± 0.002499 | 0.000038 ± 0.000075 | 0.004200 ± 0.001308 | 0.011604 ± 0.001647 | 0.000544 ± 0.000868 | 0.000703 ± 0.001407 | 0.002994 ± 0.000806 | 0.002934 ± 0.001169 | 0.002299 ± 0.002157 | 0 | 0.002935 ± 0.000734 |
| CADDYX01 | 0.060106 | 0 | 0.001482 ± 0.001872 | 0.000955 ± 0.000944 | 0 | 0.000574 ± 0.000874 | 0.000269 ± 0.000601 | 0 | 0 | 0 | 0 | 0.000278 ± 0.000622 | 0 | 0 |
| CADEDH01 | 0.510019 | 0 | 0.000139 ± 0.000277 | 0 | 0 | 0 | 0 | 0.000095 ± 0.000211 | 0 | 0 | 0 | 0 | 0 | 0.000203 ± 0.000406 |
| CAIJKY01 | 0.000535 | 0.003423 ± 0.002350 | 0.000302 ± 0.000445 | 0.000153 ± 0.000342 | 0 | 0.000106 ± 0.000236 | 0.000123 ± 0.000203 | 0.001349 ± 0.001160 | 0.002503 ± 0.000780 | 0.000503 ± 0.000616 | 0.004364 ± 0.001702 | 0 | 0.002460 ± 0.004919 | 0.004621 ± 0.007514 |
| CAILQE01 | 0.000145 | 0 | 0 | 0 | 0 | 0 | 0 | 0.001081 ± 0.000845 | 0 | 0 | 0.000237 ± 0.000531 | 0 | 0 | 0 |
| CAIPEB01 | 0.598062 | 0 | 0 | 0 | 0 | 0 | 0.000065 ± 0.000146 | 0.000140 ± 0.000312 | 0 | 0 | 0 | 0 | 0 | 0.000296 ± 0.000592 |
| CAIQIQ01 | 0.000018 | 0.024629 ± 0.004215 | 0.013658 ± 0.003152 | 0.012608 ± 0.001765 | 0.021755 ± 0.007177 | 0.023484 ± 0.002809 | 0.013491 ± 0.001329 | 0.023823 ± 0.004830 | 0.022174 ± 0.003702 | 0.020264 ± 0.005449 | 0.024680 ± 0.004239 | 0.014662 ± 0.003993 | 0.046605 ± 0.011492 | 0.033293 ± 0.004613 |
| CAISOI01 | 0.050060 | 0 | 0.000412 ± 0.000514 | 0 | 0.000495 ± 0.000990 | 0.000441 ± 0.000478 | 0.000111 ± 0.000248 | 0.000332 ± 0.000266 | 0 | 0 | 0 | 0.000222 ± 0.000497 | 0 | 0.000274 ± 0.000368 |
| CAISYW01 | 0.546132 | 0 | 0 | 0 | 0 | 0 | 0 | 0.000106 ± 0.000237 | 0 | 0 | 0 | 0 | 0 | 0 |
| CAITGV01 | 0.037830 | 0 | 0 | 0 | 0 | 0.000165 ± 0.000227 | 0 | 0 | 0 | 0 | 0 | 0 | 0 | 0 |
| CAITLU01 | 0.008521 | 0 | 0.000078 ± 0.000157 | 0 | 0 | 0.000202 ± 0.000290 | 0 | 0.000451 ± 0.000289 | 0 | 0.000360 ± 0.000335 | 0.000231 ± 0.000227 | 0 | 0.000300 ± 0.000347 | 0 |
| CAIUDO01 | 0.546132 | 0 | 0 | 0 | 0 | 0 | 0 | 0.000058 ± 0.000130 | 0 | 0 | 0 | 0 | 0 | 0 |
| CAIVZM01 | 0.731053 | 0 | 0.000108 ± 0.000215 | 0 | 0 | 0 | 0.000039 ± 0.000087 | 0 | 0 | 0 | 0.000087 ± 0.000194 | 0.000054 ± 0.00012 | 0.000156 ± 0.000312 | 0 |
| CAIXIX01 | 0.546132 | 0 | 0 | 0 | 0 | 0 | 0 | 0.000061 ± 0.000136 | 0 | 0 | 0 | 0 | 0 | 0 |
| CAIZRG01 | 0.037830 | 0 | 0 | 0 | 0 | 0 | 0 | 0.00014 ± 0.000203 | 0 | 0 | 0 | 0 | 0 | 0 |
| CAIZWU01 | 0.065880 | 0 | 0 | 0 | 0 | 0.000364 ± 0.000344 | 0 | 0 | 0 | 0 | 0.000574 ± 0.000787 | 0.000120 ± 0.000269 | 0.000965 ± 0.001930 | 0 |
| CAJBBX01_X | 0.098146 | 0 | 0 | 0 | 0 | 0.000533 ± 0.000493 | 0 | 0.000218 ± 0.000336 | 0 | 0.000508 ± 0.000703 | 0.000133 ± 0.000297 | 0.000098 ± 0.000220 | 0 | 0.000390 ± 0.000472 |
| Caldilinea | 0.001163 | 0 | 0 | 0 | 0 | 0 | 0.000023 ± 0.000052 | 0.000776 ± 0.000495 | 0 | 0.000225 ± 0.000299 | 0.000043 ± 0.000097 | 0 | 0 | 0 |
| Caldilineales_X_X | 0.316957 | 0 | 0 | 0 | 0 | 0 | 0 | 0 | 0 | 0 | 0 | 0 | 0.000854 ± 0.001709 | 0 |
| Casimicrobiaceae_X | 0.000139 | 0 | 0 | 0 | 0.000440 ± 0.000879 | 0 | 0 | 0.001461 ± 0.001221 | 0 | 0 | 0 | 0 | 0 | 0 |
| Caulobacter | 0.150299 | 0 | 0 | 0 | 0 | 0 | 0 | 0 | 0 | 0.000310 ± 0.000424 | 0 | 0.000222 ± 0.000497 | 0 | 0 |
| Caulobacteraceae_X | 0.012664 | 0.004338 ± 0.001038 | 0.002032 ± 0.001653 | 0.003053 ± 0.001787 | 0.003321 ± 0.003667 | 0.001303 ± 0.001200 | 0.004100 ± 0.001237 | 0.000286 ± 0.000424 | 0.003736 ± 0.002803 | 0.001530 ± 0.000785 | 0.001286 ± 0.000911 | 0.003638 ± 0.002161 | 0.000680 ± 0.001360 | 0.002049 ± 0.000522 |
| Cellulomonadaceae_X | 0.115463 | 0 | 0.001493 ± 0.001891 | 0.001779 ± 0.002555 | 0.001319 ± 0.001523 | 0.000933 ± 0.000915 | 0.000335 ± 0.000749 | 0.000396 ± 0.000558 | 0 | 0 | 0.000539 ± 0.000514 | 0 | 0 | 0 |
| Cellulosimicrobium | 0.510019 | 0.000426 ± 0.000852 | 0 | 0 | 0 | 0.000266 ± 0.000594 | 0 | 0 | 0 | 0 | 0 | 0 | 0.001176 ± 0.002352 | 0 |
| Cellvibrio | 0.017505 | 0.001017 ± 0.001199 | 0.000996 ± 0.001303 | 0 | 0.001145 ± 0.000821 | 0.000618 ± 0.001186 | 0 | 0.000115 ± 0.000158 | 0 | 0.005221 ± 0.005472 | 0.000738 ± 0.000497 | 0.000202 ± 0.000452 | 0.000640 ± 0.000759 | 0.000280 ± 0.000430 |
| CF-154 | 0.211127 | 0.000447 ± 0.000894 | 0 | 0 | 0 | 0 | 0 | 0.000365 ± 0.000591 | 0.000416 ± 0.000832 | 0 | 0.000331 ± 0.000465 | 0 | 0 | 0 |
| CF-163 | 0.598062 | 0 | 0 | 0 | 0 | 0.000125 ± 0.000280 | 0 | 0.000124 ± 0.000276 | 0.000761 ± 0.001521 | 0 | 0 | 0 | 0 | 0 |
| CF-167 | 0.060202 | 0.000771 ± 0.001182 | 0 | 0.000552 ± 0.000756 | 0.000958 ± 0.001109 | 0.000098 ± 0.00022 | 0.000457 ± 0.000636 | 0.002260 ± 0.001482 | 0 | 0.000711 ± 0.000995 | 0.000308 ± 0.000688 | 0 | 0.000606 ± 0.001213 | 0.000670 ± 0.000588 |
| CF-46 | 0.002102 | 0.002570 ± 0.001381 | 0.006618 ± 0.002468 | 0.008251 ± 0.002291 | 0.007016 ± 0.006300 | 0.003616 ± 0.002510 | 0.008843 ± 0.003196 | 0.006057 ± 0.000887 | 0.005469 ± 0.006209 | 0.007093 ± 0.002202 | 0.002633 ± 0.002188 | 0.001312 ± 0.001536 | 0.003290 ± 0.000952 | 0.005967 ± 0.001597 |
| Ch67 | 0.546132 | 0 | 0 | 0 | 0 | 0 | 0 | 0 | 0 | 0 | 0.000527 ± 0.001178 | 0 | 0 | 0 |
| Chelativorans | 0.000056 | 0.001382 ± 0.000444 | 0.005370 ± 0.000914 | 0.002556 ± 0.001375 | 0.003212 ± 0.001162 | 0.001948 ± 0.001092 | 0.001546 ± 0.000698 | 0.002525 ± 0.000959 | 0 | 0.000543 ± 0.000336 | 0.000792 ± 0.000598 | 0.001214 ± 0.000938 | 0.003716 ± 0.001616 | 0.001006 ± 0.001105 |
| Chitinispirillum | 0.000800 | 0 | 0 | 0 | 0 | 0 | 0 | 0.000459 ± 0.000539 | 0 | 0 | 0 | 0 | 0 | 0 |
| Chitinophaga | 0.023587 | 0.002308 ± 0.001033 | 0.001795 ± 0.000666 | 0.000811 ± 0.001281 | 0.002029 ± 0.002787 | 0.000044 ± 0.000099 | 0.001130 ± 0.001106 | 0.001499 ± 0.001080 | 0 | 0.000564 ± 0.000613 | 0.000491 ± 0.000522 | 0.001650 ± 0.001250 | 0.002327 ± 0.001070 | 0.001125 ± 0.001189 |
| Chitinophagaceae_X | 0.091854 | 0.004668 ± 0.002099 | 0.004307 ± 0.002534 | 0.005453 ± 0.002740 | 0.002154 ± 0.000609 | 0.004702 ± 0.001932 | 0.002808 ± 0.001671 | 0.003301 ± 0.001264 | 0.001105 ± 0.002210 | 0.005899 ± 0.002547 | 0.004836 ± 0.003066 | 0.006131 ± 0.006697 | 0.006404 ± 0.001561 | 0.003221 ± 0.001525 |
| Chitinophagales_X_X | 0.003867 | 0.001175 ± 0.000329 | 0.000180± 0.000361 | 0.001709 ± 0.001394 | 0.000188 ± 0.000377 | 0.000454 ± 0.000671 | 0.000132 ± 0.000184 | 0.000495 ± 0.000322 | 0 | 0 | 0.000342 ± 0.000469 | 0 | 0 | 0.000292 ± 0.000487 |
| Chloroflexaceae_X | 0.346060 | 0.000135 ± 0.000271 | 0 | 0.000147 ± 0.000329 | 0.000172 ± 0.000344 | 0.000241 ± 0.000335 | 0.000072 ± 0.000160 | 0.000211 ± 0.000326 | 0.000115 ± 0.000230 | 0.000793 ± 0.000626 | 0.000238 ± 0.000531 | 0.000138 ± 0.000308 | 0 | 0.000230 ± 0.000319 |
| Chloroflexales_X_X | 0.000309 | 0.000235 ± 0.000470 | 0.000295 ± 0.000590 | 0.000245 ± 0.000548 | 0.000126 ± 0.000251 | 0 | 0 | 0.001674 ± 0.000669 | 0 | 0.000436 ± 0.000688 | 0.001138 ± 0.000625 | 0 | 0 | 0.000205 ± 0.000248 |
| Chloroflexia_X_X_X | 0.546132 | 0 | 0 | 0 | 0 | 0 | 0.000234 ± 0.000523 | 0 | 0 | 0 | 0 | 0 | 0 | 0 |
| Chloroflexota_X_X_X_X | 0.028908 | 0.001069 ± 0.001270 | 0.000241 ± 0.000297 | 0.001028 ± 0.000691 | 0.001581 ± 0.001152 | 0.002981 ± 0.002124 | 0.002353 ± 0.001824 | 0.001676 ± 0.000556 | 0.000258 ± 0.000517 | 0.001517 ± 0.000956 | 0.001439 ± 0.000210 | 0.000561 ± 0.000814 | 0.000413 ± 0.000827 | 0.001919 ± 0.001387 |
| Chryseobacterium | 0.000081 | 0.001132 ± 0.001891 | 0.016823 ± 0.012941 | 0.070898 ± 0.069506 | 0.000786 ± 0.000910 | 0 | 0.003755 ± 0.003944 | 0.000243 ± 0.000370 | 0.026465 ± 0.028698 | 0.000550 ± 0.000866 | 0.002825 ± 0.002938 | 0.028930 ± 0.009234 | 0 | 0.000577 ± 0.001038 |
| Chryseolinea | 0.162438 | 0.002985 ± 0.000801 | 0.002777 ± 0.000907 | 0.002090 ± 0.001365 | 0.003050 ± 0.000784 | 0.001556 ± 0.000611 | 0.001633 ± 0.001175 | 0.003161 ± 0.000967 | 0.001632 ± 0.001890 | 0.002951 ± 0.001071 | 0.001978 ± 0.001060 | 0.001477 ± 0.001667 | 0.001092 ± 0.001343 | 0.002676 ± 0.001782 |
| Chryseolinea_A | 0.010320 | 0.000963 ± 0.001192 | 0.001513 ± 0.000775 | 0 | 0.001256 ± 0.001753 | 0.000093 ± 0.000207 | 0.000246 ± 0.000551 | 0.000047 ± 0.000104 | 0.000425 ± 0.000851 | 0.001310 ± 0.001753 | 0.001383 ± 0.001878 | 0.001389 ± 0.002181 | 0.006426 ± 0.004172 | 0.000997 ± 0.001760 |
| Chthoniobacter | 0.057212 | 0 | 0.000022 ± 0.000044 | 0 | 0 | 0 | 0 | 0 | 0 | 0.000017 ± 0.000025 | 0.000126 ± 0.000201 | 0.000101 ± 0.000225 | 0 | 0 |
| Chthoniobacteraceae_X | 0.017749 | 0 | 0 | 0 | 0 | 0.000521 ± 0.000729 | 0.000119 ± 0.000267 | 0.000820 ± 0.000866 | 0 | 0.000641 ± 0.000666 | 0.000822 ± 0.001127 | 0 | 0.001820 ± 0.002711 | 0.000634 ± 0.001269 |
| Chthoniobacterales_X_X | 0.424819 | 0 | 0.000255 ± 0.000336 | 0 | 0.000163 ± 0.000327 | 0.000059 ± 0.000133 | 0 | 0 | 0 | 0.000107 ± 0.000239 | 0.000018 ± 0.000039 | 0.000466 ± 0.000641 | 0 | 0.000249 ± 0.000498 |
| Clostridiaceae_X | 0.202618 | 0 | 0 | 0.000141 ± 0.000315 | 0 | 0.000162 ± 0.000181 | 0 | 0.000136 ± 0.000304 | 0.000187 ± 0.000373 | 0.000166 ± 0.000235 | 0.000218 ± 0.000218 | 0 | 0 | 0.000046 ± 0.000091 |
| Croceibacterium | 0.162230 | 0 | 0 | 0.000987 ± 0.000969 | 0.000490 ± 0.000980 | 0.000501 ± 0.000752 | 0.000359 ± 0.000802 | 0.000359 ± 0.000494 | 0.001450 ± 0.001733 | 0.000182 ± 0.000406 | 0 | 0 | 0 | 0 |
| Crossiella | 0.000005 | 0.012962 ± 0.006902 | 0 | 0 | 0 | 0 | 0 | 0.002291 ± 0.001677 | 0.014000± 0.008415 | 0.000843 ± 0.001234 | 0.000711 ± 0.000668 | 0 | 0 | 0 |
| Cryptosporangium | 0.000051 | 0.001154 ± 0.000559 | 0 | 0 | 0 | 0.000441 ± 0.000283 | 0.000246 ± 0.000551 | 0 | 0 | 0.000142 ± 0.000318 | 0.001590 ± 0.000353 | 0.001336 ± 0.002290 | 0 | 0 |
| CSP1-4_X | 0.376254 | 0.001126 ± 0.001519 | 0.000513 ± 0.001026 | 0.000546 ± 0.000937 | 0 | 0.000268 ± 0.000380 | 0.000587 ± 0.000733 | 0.000153 ± 0.000150 | 0 | 0.000459 ± 0.000690 | 0.000217 ± 0.000486 | 0.000714 ± 0.000668 | 0 | 0.000823 ± 0.000801 |
| Cupriavidus | 0.997308 | 0.001285 ± 0.001013 | 0.001323 ± 0.001726 | 0.001796 ± 0.001920 | 0.001823 ± 0.002839 | 0.001119 ± 0.001172 | 0.001103 ± 0.001228 | 0.001070 ± 0.000805 | 0.001783 ± 0.001292 | 0.001317 ± 0.001054 | 0.001271 ± 0.000816 | 0.001292 ± 0.001856 | 0.002568 ± 0.004246 | 0.000634 ± 0.001269 |
| Cyanobacteriales_X_X | 0.618381 | 0 | 0 | 0.000131 ± 0.000293 | 0 | 0 | 0 | 0 | 0 | 0 | 0.000133 ± 0.000297 | 0 | 0 | 0 |
| Cyclobacteriaceae_X | 0.012524 | 0.002058 ± 0.001018 | 0.001243 ± 0.001201 | 0.000989 ± 0.000777 | 0.003162 ± 0.001238 | 0.000452 ± 0.000386 | 0.000723 ± 0.000709 | 0.000753 ± 0.000373 | 0 | 0.000915 ± 0.000510 | 0.001911 ± 0.000330 | 0.001421 ± 0.001152 | 0.001254 ± 0.001701 | 0.002024 ± 0.001991 |
| Cystobacter | 0.006381 | 0.000614 ± 0.000951 | 0.001373 ± 0.000648 | 0.001182 ± 0.001985 | 0 | 0.000092 ± 0.000206 | 0 | 0 | 0 | 0.000107 ± 0.000239 | 0.000599 ± 0.000629 | 0 | 0 | 0.000362 ± 0.000725 |
| Cytobacillus | 0.026796 | 0 | 0 | 0 | 0 | 0 | 0.000818 ± 0.001174 | 0.000480 ± 0.000449 | 0 | 0 | 0.000175 ± 0.000391 | 0 | 0 | 0 |
| Cytophagales_X_X | 0.096377 | 0 | 0.000854 ± 0.001063 | 0 | 0.000086 ± 0.000172 | 0.000320 ± 0.000441 | 0.001237 ± 0.000941 | 0.000058 ± 0.000117 | 0 | 0.000259 ± 0.000246 | 0.000212 ± 0.000316 | 0.000299 ± 0.000668 | 0.000321 ± 0.000424 | 0.000362 ± 0.000725 |
| D16-63 | 0.051638 | 0 | 0 | 0 | 0 | 0 | 0 | 0 | 0 | 0.000319 ± 0.000714 | 0 | 0 | 0 | 0.000418 ± 0.000517 |
| Dehalococcoidia_X_X_X | 0.001014 | 0 | 0 | 0.000154 ± 0.000345 | 0 | 0.000906 ± 0.000934 | 0.001157 ± 0.000992 | 0.001655 ± 0.001308 | 0.000115 ± 0.000230 | 0.000540 ± 0.000385 | 0.000599 ± 0.000378 | 0 | 0 | 0.000203 ± 0.000406 |
| Devosia | 0.484612 | 0 | 0 | 0 | 0 | 0.000066 ± 0.000148 | 0 | 0 | 0 | 0 | 0 | 0 | 0.00045 ± 0.000901 | 0 |
| Devosiaceae_X | 0.454503 | 0.001273 ± 0.001475 | 0.000587 ± 0.001174 | 0.002130 ± 0.001226 | 0.002233 ± 0.003505 | 0.000362 ± 0.000810 | 0.000847 ± 0.001198 | 0.000337 ± 0.000753 | 0.000691 ± 0.001383 | 0.001209 ± 0.000716 | 0.000629 ± 0.000595 | 0.000328 ± 0.000732 | 0.001116 ± 0.001452 | 0.000372 ± 0.000744 |
| Domibacillaceae_X | 0.000003 | 0.004158 ± 0.000353 | 0.004577 ± 0.000711 | 0.002537 ± 0.001045 | 0.000775 ± 0.000919 | 0.002569 ± 0.000894 | 0.005556 ± 0.001272 | 0.000225 ± 0.000308 | 0.002266 ± 0.001718 | 0.000658 ± 0.000744 | 0.001544 ± 0.000671 | 0.004364 ± 0.001306 | 0.000294 ± 0.000588 | 0.005465 ± 0.001446 |
| Domibacillus | 0.000063 | 0 | 0 | 0 | 0 | 0.000337 ± 0.000754 | 0.002561 ± 0.000659 | 0 | 0 | 0 | 0 | 0.000566 ± 0.000812 | 0 | 0.000625 ± 0.000789 |
| Dongia | 0.121887 | 0 | 0 | 0 | 0.000163 ± 0.000327 | 0 | 0 | 0.000126 ± 0.000189 | 0 | 0.000190 ± 0.000263 | 0 | 0 | 0 | 0 |
| DP-1 | 0.007555 | 0.010689 ± 0.001002 | 0.005581 ± 0.003699 | 0.004716 ± 0.003177 | 0.008780 ± 0.004403 | 0.005474 ± 0.000425 | 0.004899 ± 0.002007 | 0.003692 ± 0.001261 | 0.004073 ± 0.004769 | 0.009033 ± 0.001745 | 0.008245 ± 0.002388 | 0.005731 ± 0.002337 | 0.007513 ± 0.003009 | 0.007720 ± 0.000668 |
| DP-6 | 0.018777 | 0.000217 ± 0.000433 | 0 | 0.000122 ± 0.000274 | 0 | 0.001205 ± 0.000831 | 0.000187 ± 0.000418 | 0.000600 ± 0.000592 | 0.000330 ± 0.000660 | 0.001179 ± 0.001083 | 0.000533 ± 0.000558 | 0 | 0 | 0.000272 ± 0.000544 |
| DSGV01 | 0.130613 | 0 | 0 | 0 | 0 | 0 | 0.000265 ± 0.000364 | 0.000085 ± 0.000190 | 0 | 0 | 0 | 0 | 0 | 0 |
| DSHD01 | 0.000035 | 0.000098 ± 0.000197 | 0 | 0 | 0 | 0 | 0 | 0.000890 ± 0.000165 | 0.000115 ± 0.000230 | 0 | 0.000072 ± 0.000162 | 0 | 0 | 0 |
| DSM-16500_X | 0.546132 | 0 | 0 | 0 | 0 | 0 | 0 | 0.000174 ± 0.000389 | 0 | 0 | 0 | 0 | 0 | 0 |
| DSM-18226_X | 0.048529 | 0.00182 ± 0.001218 | 0.002279 ± 0.001131 | 0.001635 ± 0.001056 | 0.002194 ± 0.002268 | 0.000491 ± 0.000605 | 0.002828 ± 0.001711 | 0.002252 ± 0.001357 | 0.004537 ± 0.001737 | 0.000832 ± 0.000814 | 0.000891 ± 0.000824 | 0.002111 ± 0.002856 | 0.003549 ± 0.002860 | 0.002666 ± 0.000920 |
| DSOM01 | 0.000279 | 0.000096 ± 0.000191 | 0 | 0.002246 ± 0.001260 | 0.000778 ± 0.000899 | 0.003998 ± 0.002135 | 0.000992 ± 0.001398 | 0.000479 ± 0.000593 | 0 | 0.002137 ± 0.000365 | 0.002807 ± 0.000881 | 0.003118 ± 0.002862 | 0 | 0.000540 ± 0.000721 |
| DSOP01 | 0.000126 | 0.000270 ± 0.000539 | 0 | 0 | 0 | 0.001734 ± 0.000594 | 0.000141 ± 0.000194 | 0.001297 ± 0.000882 | 0.000952 ± 0.000711 | 0.001897 ± 0.000650 | 0.000732 ± 0.000470 | 0 | 0.000413 ± 0.000825 | 0.000668 ± 0.000598 |
| DSPE01 | 0.013398 | 0 | 0 | 0 | 0 | 0.000655 ± 0.000466 | 0.000129 ± 0.000287 | 0.000197 ± 0.000296 | 0 | 0.000520 ± 0.000583 | 0.000133 ± 0.000297 | 0 | 0 | 0.000182 ± 0.000364 |
| DSQF01 | 0.059586 | 0 | 0 | 0 | 0 | 0 | 0 | 0.000115 ± 0.000159 | 0 | 0.000458 ± 0.000419 | 0.000127 ± 0.000180 | 0.000025 ± 0.000055 | 0 | 0.000051 ± 0.000101 |
| DSQQ01 | 0.612773 | 0.000190 ± 0.000379 | 0 | 0 | 0.000563 ± 0.001126 | 0.000125 ± 0.000280 | 0 | 0.000211 ± 0.000395 | 0 | 0.000105 ± 0.000234 | 0.000113 ± 0.000171 | 0 | 0 | 0.000220 ± 0.000440 |
| DSVQ01 | 0.000009 | 0 | 0 | 0 | 0 | 0 | 0 | 0.000835 ± 0.000237 | 0 | 0.000144 ± 0.000138 | 0.000042 ± 0.000094 | 0 | 0 | 0 |
| DSWF01 | 0.084882 | 0 | 0 | 0.000122 ± 0.000274 | 0 | 0.000130 ± 0.000290 | 0.000117 ± 0.000261 | 0 | 0 | 0.000359 ± 0.000518 | 0.000679 ± 0.000662 | 0 | 0 | 0.000466 ± 0.000586 |
| DSWX01 | 0.040503 | 0 | 0 | 0 | 0 | 0 | 0 | 0 | 0 | 0 | 0 | 0.000046 ± 0.000103 | 0 | 0.000650 ± 0.000987 |
| DSXL01 | 0.150299 | 0 | 0 | 0.000070 ± 0.000158 | 0 | 0 | 0 | 0.000087 ± 0.000126 | 0 | 0 | 0 | 0 | 0 | 0 |
| DSYW01 | 0.000656 | 0.002257 ± 0.000682 | 0.001644 ± 0.001356 | 0.001426 ± 0.001015 | 0.000352 ± 0.000703 | 0.007207 ± 0.002021 | 0.003370 ± 0.002433 | 0.003016 ± 0.001294 | 0.000397 ± 0.000795 | 0.002570 ± 0.001605 | 0.004816 ± 0.001354 | 0.003213 ± 0.001795 | 0.001611 ± 0.002415 | 0.003690 ± 0.002454 |
| DTNO01 | 0.000164 | 0.002768 ± 0.000590 | 0.000225 ± 0.000450 | 0.001715 ± 0.001046 | 0.000327 ± 0.000653 | 0.000980 ± 0.001079 | 0.000223 ± 0.000380 | 0.001280 ± 0.001454 | 0.004592 ± 0.004067 | 0.007513 ± 0.002079 | 0.005671 ± 0.001264 | 0.003093 ± 0.001843 | 0.004318 ± 0.003078 | 0.003035 ± 0.000705 |
| Dyadobacter | 0.006013 | 0.001068 ± 0.000485 | 0.003399 ± 0.003280 | 0.004757 ± 0.003451 | 0.000678 ± 0.000865 | 0.000575 ± 0.000427 | 0.003146 ± 0.003590 | 0.000175 ± 0.000300 | 0.001831 ± 0.001270 | 0.000745 ± 0.000513 | 0.001276 ± 0.000749 | 0.009758 ± 0.004072 | 0.000349 ± 0.000698 | 0.000351 ± 0.000537 |
| Dyella | 0.692967 | 0 | 0 | 0 | 0 | 0 | 0 | 0 | 0 | 0.000107 ± 0.000239 | 0.000084 ± 0.000189 | 0.000074 ± 0.000165 | 0 | 0 |
| Ectobacillus | 0.000089 | 0.002108 ± 0.001135 | 0.002306 ± 0.001116 | 0.007238 ± 0.002912 | 0.001325 ± 0.001555 | 0.002336 ± 0.000866 | 0.004756 ± 0.002055 | 0.000748 ± 0.000270 | 0.004465 ± 0.006244 | 0.001105 ± 0.000426 | 0.001589 ± 0.000480 | 0.004681 ± 0.001954 | 0.000282 ± 0.000449 | 0.001114 ± 0.000398 |
| Edaphocola | 0.266311 | 0 | 0.000055 ± 0.000111 | 0.000278 ± 0.000621 | 0 | 0.000074 ± 0.000166 | 0.000064 ± 0.000144 | 0 | 0 | 0 | 0.000260 ± 0.000280 | 0.000297 ± 0.000665 | 0 | 0 |
| Egibacteraceae_X | 0.074871 | 0 | 0 | 0 | 0 | 0.000158 ± 0.000252 | 0.000329 ± 0.000735 | 0 | 0 | 0 | 0 | 0 | 0 | 0.000228 ± 0.000291 |
| Elainellaceae_X | 0.598062 | 0 | 0 | 0 | 0 | 0 | 0 | 0 | 0.000115 ± 0.000230 | 0.000062 ± 0.000139 | 0 | 0.000061 ± 0.000137 | 0 | 0 |
| Elizabethkingia | 0.484612 | 0 | 0 | 0 | 0.000729 ± 0.001458 | 0 | 0 | 0 | 0 | 0 | 0.000358 ± 0.000801 | 0 | 0 | 0 |
| Emticicia | 0.037830 | 0 | 0 | 0 | 0 | 0 | 0 | 0 | 0 | 0.000203 ± 0.000281 | 0 | 0 | 0 | 0 |
| Enhygromyxa | 0.248656 | 0 | 0 | 0 | 0 | 0 | 0 | 0.000554 ± 0.000774 | 0 | 0.000077 ± 0.000172 | 0.000101 ± 0.000226 | 0 | 0 | 0 |
| Enterobacter | 0.173176 | 0 | 0.001051 ± 0.000885 | 0.002852 ± 0.003482 | 0.002858 ± 0.003132 | 0.001476 ± 0.002033 | 0.000151 ± 0.000338 | 0 | 0.001133 ± 0.001326 | 0.002677 ± 0.004534 | 0.000975 ± 0.001202 | 0.000770 ± 0.000765 | 0.002003 ± 0.004006 | 0.000068 ± 0.000135 |
| Enterobacter_D | 0.076758 | 0.001499 ± 0.001801 | 0.003652 ± 0.002670 | 0.004194 ± 0.004066 | 0.001234 ± 0.001431 | 0.004126 ± 0.007094 | 0.001134 ± 0.001297 | 0.000048 ± 0.000108 | 0.001530 ± 0.002621 | 0.002770 ± 0.002036 | 0.001646 ± 0.000405 | 0.003315 ± 0.002270 | 0.000239 ± 0.000478 | 0.000137 ± 0.000273 |
| Enterobacteriaceae_X | 0.094770 | 0.001729 ± 0.002587 | 0.004069 ± 0.003243 | 0.003538 ± 0.004060 | 0.001028 ± 0.001189 | 0.002250 ± 0.002724 | 0.005935 ± 0.004848 | 0.000356 ± 0.000543 | 0.000992 ± 0.001984 | 0.002293 ± 0.005126 | 0.001324 ± 0.001820 | 0.013548 ± 0.014671 | 0.000964 ± 0.000684 | 0.002732 ± 0.004958 |
| Ereboglobus | 0.016588 | 0.001042 ± 0.000723 | 0.000157 ± 0.000313 | 0.000754 ± 0.000807 | 0.001551 ± 0.001765 | 0.001971 ± 0.000610 | 0.000187 ± 0.000418 | 0.001459 ± 0.000513 | 0.001820 ± 0.001453 | 0.001966 ± 0.000410 | 0.000731 ± 0.000807 | 0.002023 ± 0.002048 | 0.000992 ± 0.001616 | 0.000442 ± 0.000512 |
| ETA-A8 | 0.000221 | 0.000344 ± 0.000419 | 0.000652 ± 0.000444 | 0.000622 ± 0.000634 | 0.000684 ± 0.000801 | 0.000914 ± 0.000548 | 0.000607 ± 0.001107 | 0.005624 ± 0.001130 | 0.002737 ± 0.001932 | 0.002971 ± 0.000952 | 0.001793 ± 0.000766 | 0 | 0.000925 ± 0.000626 | 0.000650 ± 0.000586 |
| Euzebya | 0.407846 | 0.000338 ± 0.00046 | 0 | 0 | 0.000717 ± 0.001434 | 0.000359 ± 0.000370 | 0.000035 ± 0.000078 | 0.000226 ± 0.000213 | 0.000183 ± 0.000365 | 0 | 0.000109 ± 0.000243 | 0.000131 ± 0.000292 | 0.000101 ± 0.000202 | 0.000354 ± 0.000409 |
| EW11 | 0.370923 | 0 | 0.000207 ± 0.000413 | 0 | 0 | 0 | 0 | 0 | 0 | 0 | 0 | 0 | 0.000464 ± 0.000928 | 0 |
| FEN-1191 | 0.000112 | 0.000235 ± 0.00047 | 0.000137 ± 0.000274 | 0 | 0.000301 ± 0.000603 | 0.000280 ± 0.000291 | 0 | 0.000830 ± 0.000233 | 0.001105 ± 0.001317 | 0.001765 ± 0.000315 | 0.001183 ± 0.000516 | 0.000098 ± 0.000220 | 0 | 0.000136 ± 0.000272 |
| Fen-1342_X | 0.055472 | 0 | 0 | 0 | 0 | 0 | 0 | 0.000242 ± 0.000234 | 0.000100 ± 0.000201 | 0.000149 ± 0.000225 | 0.000077 ± 0.000172 | 0 | 0 | 0 |
| Ferrovibrio | 0.513595 | 0.000427 ± 0.000495 | 0.000301 ± 0.000357 | 0.000104 ± 0.000232 | 0 | 0.000327 ± 0.000321 | 0.000319 ± 0.000327 | 0.000061 ± 0.000136 | 0.000244 ± 0.000488 | 0.000336 ± 0.000338 | 0.000042 ± 0.000094 | 0.000383 ± 0.000857 | 0.000165 ± 0.000331 | 0.000416 ± 0.000278 |
| Fimbriiglobus | 0.477662 | 0.000971 ± 0.000733 | 0.001111 ± 0.000836 | 0.001257 ± 0.001819 | 0.000946 ± 0.001893 | 0.001797 ± 0.001357 | 0.001226 ± 0.002285 | 0.001640 ± 0.000991 | 0.000445 ± 0.000890 | 0.002020 ± 0.001028 | 0.002590 ± 0.001207 | 0.001698 ± 0.001871 | 0.001414 ± 0.001328 | 0.001489 ± 0.000532 |
| Fimbriimonadaceae_X | 0.316957 | 0 | 0 | 0 | 0 | 0 | 0 | 0 | 0 | 0 | 0 | 0 | 0.000689 ± 0.001378 | 0 |
| Firmicutes_A_X_X_X_X | 0.130613 | 0 | 0 | 0 | 0 | 0 | 0.000129 ± 0.000176 | 0.000034 ± 0.000076 | 0 | 0 | 0 | 0 | 0 | 0 |
| Flavihumibacter | 0.123041 | 0 | 0 | 0 | 0 | 0.000087 ± 0.000194 | 0 | 0.000034 ± 0.000076 | 0.000209 ± 0.000418 | 0.000200 ± 0.000254 | 0.000245 ± 0.000336 | 0 | 0 | 0 |
| Flavisolibacter | 0.064357 | 0.008933 ± 0.001523 | 0.007615 ± 0.003788 | 0.007049 ± 0.002317 | 0.007501 ± 0.006273 | 0.008621 ± 0.001424 | 0.007724 ± 0.002232 | 0.005229 ± 0.000346 | 0.004559 ± 0.003588 | 0.004708 ± 0.000876 | 0.007058 ± 0.002136 | 0.010411 ± 0.004871 | 0.012426 ± 0.005944 | 0.007189 ± 0.003662 |
| Flavisolibacter_B | 0.003180 | 0.001179 ± 0.000917 | 0 | 0 | 0 | 0.000154 ± 0.000225 | 0.000810 ± 0.000610 | 0 | 0 | 0.000470 ± 0.000293 | 0.000053 ± 0.000104 | 0.000107 ± 0.000240 | 0.000532 ± 0.001013 | 0.000448 ± 0.000372 |
| Flavobacteriaceae_X | 0.002125 | 0 | 0 | 0 | 0 | 0.000245 ± 0.000153 | 0 | 0 | 0 | 0.000071 ± 0.000159 | 0.000079 ± 0.000178 | 0 | 0 | 0 |
| Flavobacterium | 0.037123 | 0.001837 ± 0.000618 | 0.000752 ± 0.000895 | 0.001965 ± 0.003301 | 0.002908 ± 0.004659 | 0.002130 ± 0.002675 | 0.000377 ± 0.000520 | 0.000057 ± 0.000079 | 0.001243 ± 0.001529 | 0.013022 ± 0.014404 | 0.003207 ± 0.003244 | 0.001722 ± 0.001285 | 0.001753 ± 0.002032 | 0.000450 ± 0.000583 |
| Fluviicola | 0.370923 | 0.000379 ± 0.000759 | 0 | 0 | 0 | 0 | 0 | 0 | 0 | 0 | 0 | 0 | 0 | 0.000068 ± 0.000136 |
| Frankia | 0.208742 | 0.008754 ± 0.002869 | 0.008438 ± 0.004444 | 0.004280 ± 0.005707 | 0.002122 ± 0.002297 | 0.000554 ± 0.000521 | 0.004571 ± 0.003829 | 0.002063 ± 0.002006 | 0.008889 ± 0.013290 | 0.004875 ± 0.004879 | 0.004043 ± 0.007370 | 0.001742 ± 0.002794 | 0.009713 ± 0.008881 | 0.003660 ± 0.005141 |
| Fuerstia | 0.002031 | 0.000082 ± 0.000164 | 0.000400 ± 0.000321 | 0.000141 ± 0.000315 | 0 | 0.000066 ± 0.000148 | 0 | 0.000418 ± 0.000432 | 0 | 0.000315 ± 0.000294 | 0.000808 ± 0.000251 | 0.000082 ± 0.000183 | 0 | 0.000063 ± 0.000126 |
| FW602-bin22 | 0.003315 | 0 | 0 | 0 | 0 | 0.001605 ± 0.001434 | 0 | 0.000281 ± 0.000392 | 0 | 0.000696 ± 0.000637 | 0.000159 ± 0.000355 | 0.000337 ± 0.000488 | 0 | 0 |
| Ga0077529_X | 0.000079 | 0.000261 ± 0.000522 | 0.000097 ± 0.000194 | 0 | 0.000510 ± 0.001021 | 0.000977 ± 0.000624 | 0.000318 ± 0.000443 | 0.003051 ± 0.001491 | 0.002222 ± 0.001543 | 0.003034 ± 0.001696 | 0.001660 ± 0.000619 | 0.000221 ± 0.000494 | 0 | 0.001414 ± 0.001319 |
| Ga0077541 | 0.546132 | 0 | 0 | 0 | 0 | 0 | 0 | 0.000075 ± 0.000168 | 0 | 0 | 0 | 0 | 0 | 0 |
| Ga0077545 | 0.298983 | 0 | 0 | 0.000112 ± 0.000251 | 0 | 0 | 0 | 0 | 0 | 0.000089 ± 0.000122 | 0 | 0.00013 ± 0.000291 | 0 | 0 |
| Ga0077550 | 0.006548 | 0 | 0 | 0 | 0 | 0.000408 ± 0.000390 | 0.000041 ± 0.000091 | 0 | 0 | 0 | 0 | 0 | 0 | 0 |
| Ga0077555 | 0.130613 | 0 | 0 | 0 | 0 | 0.000335 ± 0.000492 | 0 | 0.000087 ± 0.000195 | 0 | 0 | 0 | 0 | 0 | 0 |
| Gaiella | 0.005511 | 0.001263 ± 0.001459 | 0.004698 ± 0.001193 | 0.002576 ± 0.001336 | 0.003150 ± 0.002107 | 0.001694 ± 0.000505 | 0.002044 ± 0.000811 | 0.002932 ± 0.000835 | 0.002524 ± 0.003021 | 0.001279 ± 0.001489 | 0.000779 ± 0.000645 | 0.000614 ± 0.001373 | 0.000377 ± 0.000753 | 0.000259 ± 0.000316 |
| Gaiellaceae_X | 0.015288 | 0.005828 ± 0.001092 | 0.002113 ± 0.002029 | 0.006107 ± 0.002804 | 0.002934 ± 0.004186 | 0.003274 ± 0.001927 | 0.007684 ± 0.001651 | 0.006302 ± 0.002262 | 0.014596 ± 0.006889 | 0.006260 ± 0.002778 | 0.006521 ± 0.003403 | 0.005769 ± 0.003403 | 0.002132 ± 0.001499 | 0.007002 ± 0.001867 |
| Gammaproteobacteria_X_X_X | 0.000097 | 0.004442 ± 0.000453 | 0.002238 ± 0.000611 | 0.001892 ± 0.000530 | 0.003064 ± 0.000736 | 0.004697 ± 0.001178 | 0.004225 ± 0.000925 | 0.008539 ± 0.000606 | 0.002517 ± 0.003005 | 0.005485 ± 0.000822 | 0.003887 ± 0.001344 | 0.002082 ± 0.001407 | 0.004523 ± 0.001359 | 0.004993 ± 0.001225 |
| GCA-002050365 | 0.020571 | 0.001364 ± 0.000955 | 0.001852 ± 0.002296 | 0.000587 ± 0.000804 | 0.007072 ± 0.007082 | 0.001521 ± 0.000750 | 0.000993 ± 0.000797 | 0.003359 ± 0.001381 | 0 | 0.002784 ± 0.001149 | 0.002011 ± 0.000901 | 0.002638 ± 0.002413 | 0.001066 ± 0.001618 | 0.001411 ± 0.000944 |
| GCA-002686595_X | 0.002517 | 0 | 0 | 0 | 0 | 0.000074 ± 0.000166 | 0 | 0.000576 ± 0.000436 | 0 | 0.000128 ± 0.000211 | 0 | 0 | 0 | 0.000246 ± 0.000287 |
| GCA-2723275 | 0.000020 | 0 | 0 | 0 | 0 | 0 | 0 | 0.001680 ± 0.000760 | 0 | 0.000072 ± 0.000160 | 0.000175 ± 0.000240 | 0 | 0.000861 ± 0.000668 | 0 |
| GCA-2746885 | 0.005405 | 0.000887 ± 0.001238 | 0.001796 ± 0.000720 | 0.000351 ± 0.000610 | 0.001784 ± 0.002027 | 0 | 0.000397 ± 0.000671 | 0.013591 ± 0.004878 | 0.002477 ± 0.003000 | 0.002674 ± 0.002851 | 0.002391 ± 0.004015 | 0.000735 ± 0.001034 | 0.000844 ± 0.001061 | 0.000129 ± 0.000258 |
| GCA-2862545_X | 0.037830 | 0 | 0 | 0 | 0 | 0 | 0 | 0 | 0 | 0 | 0.000236 ± 0.000386 | 0 | 0 | 0 |
| GCA-900066495 | 0.240889 | 0 | 0 | 0 | 0.000691 ± 0.001382 | 0.000302 ± 0.000676 | 0.000401 ± 0.000565 | 0.000088 ± 0.000138 | 0 | 0 | 0 | 0 | 0 | 0 |
| Geminicoccaceae_X | 0.000673 | 0.005565 ± 0.004242 | 0.004711 ± 0.004739 | 0.004393 ± 0.002147 | 0.005165 ± 0.005220 | 0.001716 ± 0.00235 | 0.001337 ± 0.002135 | 0.024827 ± 0.007076 | 0.003930 ± 0.003179 | 0.002209 ± 0.001288 | 0.011011 ± 0.004162 | 0.000683 ± 0.000957 | 0.003238 ± 0.002192 | 0.006532 ± 0.003512 |
| Gemmata | 0.000002 | 0.004133 ± 0.001054 | 0.002436 ± 0.001774 | 0.001445 ± 0.001394 | 0.006497 ± 0.002261 | 0.002673 ± 0.001613 | 0.001558 ± 0.001004 | 0.009609 ± 0.002754 | 0.009040 ± 0.003154 | 0.011137 ± 0.002565 | 0.008711 ± 0.000572 | 0.000401 ± 0.000897 | 0.010017 ± 0.003662 | 0.004970 ± 0.001311 |
| Gemmataceae_X | 0.004360 | 0.007196 ± 0.001744 | 0.002569 ± 0.003033 | 0.002963 ± 0.001726 | 0.003286 ± 0.004865 | 0.007210 ± 0.003463 | 0.006347 ± 0.003547 | 0.009396 ± 0.001686 | 0.011652 ± 0.005750 | 0.012138 ± 0.006078 | 0.013997 ± 0.003047 | 0.004408 ± 0.003007 | 0.007039 ± 0.006515 | 0.011265 ± 0.005748 |
| Gemmatales_X_X | 0.261371 | 0 | 0.000264 ± 0.000528 | 0.000528 ± 0.001180 | 0.000214 ± 0.000427 | 0 | 0 | 0.000138 ± 0.000309 | 0 | 0.000136 ± 0.000304 | 0.001013 ± 0.001618 | 0 | 0 | 0 |
| Gemmatimonadaceae_X | 0.005922 | 0.001037 ± 0.000905 | 0.000388 ± 0.000485 | 0.001775 ± 0.000846 | 0.000545 ± 0.000681 | 0.004754 ± 0.001159 | 0.001010 ± 0.000694 | 0.002731 ± 0.001757 | 0.003135 ± 0.002386 | 0.000850 ± 0.000492 | 0.002556 ± 0.001155 | 0.001409 ± 0.001465 | 0.000705 ± 0.001036 | 0.001825 ± 0.001228 |
| Gemmatimonadetes_X_X_X | 0.079152 | 0.000810 ± 0.001002 | 0.000665 ± 0.000941 | 0.000088 ± 0.000197 | 0.001358 ± 0.001582 | 0.001219 ± 0.000882 | 0.001482 ± 0.001426 | 0.002038 ± 0.001192 | 0 | 0.000293 ± 0.000402 | 0.000703 ± 0.000394 | 0.000337 ± 0.000490 | 0.000635 ± 0.001269 | 0.001114 ± 0.000582 |
| Gemmatimonas | 0.026796 | 0 | 0 | 0 | 0 | 0.000268 ± 0.000342 | 0 | 0 | 0 | 0.000355 ± 0.000486 | 0.000126 ± 0.000281 | 0 | 0 | 0 |
| Gemmatirosa | 0.000080 | 0.001161 ± 0.001361 | 0.000127 ± 0.000254 | 0.000509 ± 0.000697 | 0 | 0.003846 ± 0.000746 | 0.004305 ± 0.002679 | 0.000897 ± 0.000846 | 0.000244 ± 0.000488 | 0.001715 ± 0.000824 | 0.001775 ± 0.001687 | 0.000222 ± 0.000497 | 0.000098 ± 0.000197 | 0.001765 ± 0.001171 |
| Geobacteraceae_X | 0.187026 | 0 | 0.000236 ± 0.000472 | 0 | 0 | 0 | 0 | 0.000085 ± 0.000189 | 0 | 0 | 0.000130 ± 0.000291 | 0 | 0 | 0.000446 ± 0.000523 |
| Geodermatophilaceae_X | 0.248489 | 0.000650 ± 0.001300 | 0.001518 ± 0.001813 | 0.000493 ± 0.000684 | 0.000892 ± 0.001784 | 0.000473 ± 0.000718 | 0.002801 ± 0.000910 | 0.001633 ± 0.001056 | 0.002467 ± 0.003287 | 0.001481 ± 0.001053 | 0.001521 ± 0.001217 | 0.001696 ± 0.002323 | 0 | 0.001518 ± 0.001791 |
| Geodermatophilus | 0.009610 | 0 | 0.004296 ± 0.002969 | 0 | 0 | 0.001101 ± 0.001508 | 0.009994 ± 0.006344 | 0 | 0 | 0.000617 ± 0.001379 | 0.000858 ± 0.001320 | 0.002835 ± 0.004069 | 0 | 0.001691 ± 0.003383 |
| GMQP-bins7 | 0.597065 | 0.000443 ± 0.000886 | 0 | 0 | 0.000641 ± 0.001281 | 0.000112 ± 0.000250 | 0.000375 ± 0.000524 | 0.000560 ± 0.000791 | 0.001254 ± 0.001566 | 0 | 0.000175 ± 0.000391 | 0 | 0.000668 ± 0.001336 | 0.000220 ± 0.000440 |
| Gp18-AA60 | 0.173187 | 0.000104 ± 0.000209 | 0 | 0 | 0 | 0.000280 ± 0.000305 | 0 | 0.000107 ± 0.000239 | 0.000239 ± 0.000479 | 0.000283 ± 0.000277 | 0.000107 ± 0.000239 | 0 | 0 | 0.000135 ± 0.000269 |
| Gp1-AA17 | 0.005580 | 0 | 0.000472 ± 0.000547 | 0 | 0 | 0 | 0 | 0 | 0 | 0 | 0 | 0 | 0 | 0 |
| Gp6-AA40 | 0.000103 | 0.010020 ± 0.001025 | 0.002465 ± 0.002924 | 0.008229 ± 0.003385 | 0.004077 ± 0.001627 | 0.018023 ± 0.004338 | 0.007375 ± 0.003334 | 0.014047 ± 0.002976 | 0.008952 ± 0.003499 | 0.014361 ± 0.001435 | 0.012825 ± 0.002902 | 0.007096 ± 0.004328 | 0.005918 ± 0.005117 | 0.011809 ± 0.006924 |
| Gp6-AA56 | 0.000001 | 0 | 0 | 0 | 0 | 0 | 0 | 0 | 0 | 0 | 0.001716 ± 0.000544 | 0 | 0 | 0.001106 ± 0.000738 |
| Gp7-AA10 | 0.029172 | 0.004951 ± 0.001678 | 0.001863 ± 0.001992 | 0.003827 ± 0.000910 | 0.003058 ± 0.002045 | 0.002349 ± 0.000777 | 0.003146 ± 0.001460 | 0.003010 ± 0.000366 | 0.005210 ± 0.002585 | 0.002682 ± 0.000385 | 0.003483 ± 0.000865 | 0.005268 ± 0.001239 | 0.003349 ± 0.002564 | 0.004553 ± 0.000644 |
| GWA2-73-35_X | 0.004996 | 0.006906 ± 0.001588 | 0.001448 ± 0.001683 | 0.002162 ± 0.002208 | 0.006822 ± 0.005536 | 0.009791 ± 0.003013 | 0.006909 ± 0.003034 | 0.007102 ± 0.002610 | 0.006989 ± 0.004677 | 0.005536 ± 0.001455 | 0.005493 ± 0.001522 | 0.003105 ± 0.003605 | 0.002780 ± 0.002036 | 0.008634 ± 0.002870 |
| GWC2-71-9_X | 0.114259 | 0.000148 ± 0.000296 | 0.000362 ± 0.000724 | 0 | 0 | 0.000310 ± 0.000347 | 0.000070 ± 0.000155 | 0.001028 ± 0.000690 | 0.000258 ± 0.000517 | 0.000851 ± 0.000928 | 0.000424 ± 0.000586 | 0.000178 ± 0.000399 | 0 | 0.000353 ± 0.000462 |
| GWC2-73-18 | 0.350797 | 0 | 0 | 0.000143 ± 0.000320 | 0.001897 ± 0.003794 | 0.001062 ± 0.001148 | 0.000160 ± 0.000219 | 0.000929 ± 0.001273 | 0.000741 ± 0.000874 | 0.000116 ± 0.000259 | 0.000460 ± 0.000524 | 0.000624 ± 0.001141 | 0 | 0 |
| Haliangium | 0.337371 | 0 | 0 | 0 | 0 | 0.000249 ± 0.000375 | 0 | 0.000059 ± 0.000133 | 0 | 0.000116 ± 0.000260 | 0.000237 ± 0.000406 | 0 | 0 | 0.000188 ± 0.000377 |
| Halieaceae_X | 0.008701 | 0 | 0 | 0 | 0 | 0 | 0 | 0 | 0 | 0.000083 ± 0.000185 | 0.000252 ± 0.000250 | 0 | 0 | 0 |
| Haloactinopolyspora | 0.002130 | 0.000217 ± 0.000433 | 0.001767 ± 0.000638 | 0.000787 ± 0.001112 | 0 | 0.000593 ± 0.000624 | 0.000117 ± 0.000261 | 0.001791 ± 0.001154 | 0 | 0.000285 ± 0.000464 | 0.001876 ± 0.001331 | 0 | 0 | 0.000498 ± 0.000619 |
| Haloferula | 0.002557 | 0.004062 ± 0.001737 | 0.009995 ± 0.003079 | 0.007919 ± 0.002667 | 0.007748 ± 0.005933 | 0.005417 ± 0.005473 | 0.005227 ± 0.001767 | 0.006037 ± 0.003981 | 0.000651 ± 0.001141 | 0.004802 ± 0.002615 | 0.007010 ± 0.002341 | 0.005340 ± 0.003178 | 0.025205 ± 0.013338 | 0.003162 ± 0.001970 |
| Halothermothrix | 0.140505 | 0 | 0 | 0 | 0 | 0 | 0.000087 ± 0.000195 | 0 | 0 | 0 | 0.000136 ± 0.000242 | 0 | 0 | 0 |
| Hamadaea | 0.604213 | 0 | 0 | 0 | 0 | 0 | 0 | 0 | 0 | 0.000155 ± 0.000347 | 0.000097 ± 0.000216 | 0 | 0.000177 ± 0.000354 | 0 |
| Herpetosiphon | 0.593416 | 0 | 0.000544 ± 0.000650 | 0.000245 ± 0.000548 | 0 | 0.000278 ± 0.000389 | 0.000088 ± 0.000196 | 0.000423 ± 0.000447 | 0 | 0.000246 ± 0.000549 | 0.000664 ± 0.001485 | 0.000330 ± 0.000739 | 0.000211 ± 0.000423 | 0 |
| HG15A2 | 0.054494 | 0 | 0.000215 ± 0.000431 | 0.000330 ± 0.000737 | 0 | 0.000155 ± 0.000234 | 0.000971 ± 0.000972 | 0.000662 ± 0.000816 | 0.000589 ± 0.000680 | 0.000683 ± 0.000388 | 0.000595 ± 0.000532 | 0 | 0.000386 ± 0.000772 | 0.000432 ± 0.000303 |
| HGW-BRC1-1 | 0.457737 | 0 | 0.000166 ± 0.000333 | 0 | 0 | 0 | 0.000076 ± 0.000170 | 0.000238 ± 0.000264 | 0.000266 ± 0.000532 | 0.000102 ± 0.000142 | 0.000094 ± 0.000210 | 0 | 0.000156 ± 0.000312 | 0.000054 ± 0.000108 |
| HMF7647 | 0.604213 | 0 | 0.000236 ± 0.000472 | 0 | 0 | 0 | 0.000210 ± 0.000470 | 0 | 0 | 0 | 0 | 0.000107 ± 0.000239 | 0 | 0 |
| HRBIN12_X | 0.023155 | 0.001294 ± 0.001209 | 0.002671 ± 0.003482 | 0.001645 ± 0.001602 | 0.00194 ± 0.002242 | 0.001333 ± 0.000834 | 0.001492 ± 0.001072 | 0.000148 ± 0.000331 | 0.005834 ± 0.002241 | 0.000697 ± 0.00075 | 0.003173 ± 0.001568 | 0.001475 ± 0.001434 | 0 | 0.001761 ± 0.001735 |
| Humibacillus | 0.067241 | 0.000252 ± 0.000504 | 0 | 0 | 0 | 0 | 0 | 0.000661 ± 0.000604 | 0 | 0.000393 ± 0.000539 | 0.000129 ± 0.000289 | 0 | 0.000156 ± 0.000312 | 0 |
| Hyalangium | 0.007439 | 0.002044 ± 0.001697 | 0.001887 ± 0.001315 | 0.001105 ± 0.001084 | 0.003316 ± 0.0013 | 0.001234 ± 0.000841 | 0.002318 ± 0.000707 | 0.000606 ± 0.000683 | 0.001091 ± 0.002182 | 0.000692 ± 0.001047 | 0.000108 ± 0.000242 | 0.003769 ± 0.002649 | 0.001573 ± 0.001215 | 0.001193 ± 0.000884 |
| Hydrogenedentiales_X_X | 0.298983 | 0 | 0 | 0.000141 ± 0.000315 | 0 | 0 | 0 | 0 | 0 | 0.000098 ± 0.000167 | 0.001011 ± 0.00226 | 0 | 0 | 0 |
| Hydrogenispora | 0.610162 | 0 | 0 | 0 | 0 | 0.000106 ± 0.000237 | 0 | 0.000194 ± 0.000434 | 0 | 0 | 0 | 0 | 0 | 0.000102 ± 0.000204 |
| Hydrogenophaga | 0.316957 | 0 | 0 | 0 | 0 | 0 | 0 | 0 | 0 | 0 | 0 | 0 | 0.000211 ± 0.000423 | 0 |
| Hymenobacteraceae_X | 0.000166 | 0.000287 ± 0.000574 | 0 | 0.000231 ± 0.000517 | 0.000854 ± 0.001708 | 0 | 0.002233 ± 0.000916 | 0 | 0 | 0.000159 ± 0.000355 | 0 | 0.00158 ± 0.001582 | 0 | 0.001407 ± 0.000151 |
| Hyphomicrobiaceae_X | 0.648584 | 0.000217 ± 0.000435 | 0.000266 ± 0.000531 | 0 | 0 | 0.000145 ± 0.000323 | 0.000378 ± 0.000845 | 0.000452 ± 0.000423 | 0 | 0.000374 ± 0.000603 | 0.000166 ± 0.000371 | 0.000153 ± 0.000343 | 0.000531 ± 0.001061 | 0 |
| Hyphomicrobium | 0.000974 | 0 | 0 | 0.000347 ± 0.000776 | 0 | 0 | 0 | 0.001241 ± 0.000817 | 0 | 0 | 0 | 0 | 0.000294 ± 0.000588 | 0 |
| Hyphomicrobium_A | 0.098651 | 0 | 0 | 0.000259 ± 0.000393 | 0 | 0.000063 ± 0.000142 | 0.000263 ± 0.000368 | 0.000377 ± 0.000588 | 0 | 0.000257 ± 0.00024 | 0.000197 ± 0.000287 | 0 | 0 | 0 |
| Hyphomonadaceae_X | 0.147654 | 0.001474 ± 0.000586 | 0.001152 ± 0.000814 | 0.001064 ± 0.001001 | 0.00167 ± 0.001975 | 0.001774 ± 0.000738 | 0.001005 ± 0.000649 | 0.001215 ± 0.001229 | 0.002166 ± 0.000542 | 0.001522 ± 0.000786 | 0.001155 ± 0.000393 | 0.000377 ± 0.000842 | 0.000211 ± 0.000423 | 0.001711 ± 0.000328 |
| Ilumatobacter | 0.027373 | 0.002576 ± 0.001348 | 0.001496 ± 0.001398 | 0.000825 ± 0.001846 | 0.002833 ± 0.003278 | 0.004723 ± 0.00083 | 0.001171 ± 0.001154 | 0.00342 ± 0.000947 | 0.002306 ± 0.002667 | 0.002421 ± 0.001829 | 0.002482 ± 0.001149 | 0.002248 ± 0.001377 | 0 | 0.003081 ± 0.000891 |
| Ilumatobacteraceae_X | 0.054303 | 0 | 0.001174 ± 0.00136 | 0 | 0 | 0.000371 ± 0.00054 | 0 | 0.000303 ± 0.000678 | 0 | 0.000814 ± 0.001152 | 0.000392 ± 0.000877 | 0 | 0.002394 ± 0.001786 | 0.000448 ± 0.000896 |
| IMCC26134_A | 0.130613 | 0 | 0 | 0 | 0 | 0.000062 ± 0.000138 | 0 | 0 | 0 | 0.000283 ± 0.000388 | 0 | 0 | 0 | 0 |
| IMCC26256_X_X | 0.236850 | 0 | 0 | 0 | 0 | 0 | 0 | 0 | 0.000585 ± 0.001171 | 0.000159 ± 0.000355 | 0.000329 ± 0.000496 | 0 | 0 | 0 |
| IMCC3088 | 0.000177 | 0 | 0 | 0 | 0 | 0 | 0 | 0 | 0 | 0.000213 ± 0.000192 | 0.000036 ± 0.000081 | 0 | 0 | 0 |
| Indioceanicola | 0.023688 | 0.000261 ± 0.000522 | 0 | 0 | 0 | 0 | 0 | 0.000375 ± 0.000364 | 0 | 0 | 0.000323 ± 0.000494 | 0 | 0 | 0 |
| Inquilinus | 0.297735 | 0.000788 ± 0.000948 | 0.000127 ± 0.000254 | 0 | 0.000102 ± 0.000205 | 0.000264 ± 0.000430 | 0.000222 ± 0.000336 | 0.000117 ± 0.000263 | 0 | 0 | 0 | 0.000417 ± 0.000634 | 0 | 0 |
| Isosphaeraceae_X | 0.603805 | 0.000592 ± 0.000835 | 0.000502 ± 0.001003 | 0.000584 ± 0.001306 | 0.000427 ± 0.000854 | 0.000905 ± 0.000724 | 0.000338 ± 0.000656 | 0.000583 ± 0.000673 | 0.000789 ± 0.001579 | 0.000245 ± 0.000386 | 0.000971 ± 0.001088 | 0 | 0.000818 ± 0.000948 | 0.000588 ± 0.000973 |
| J027_X | 0.037830 | 0 | 0 | 0 | 0 | 0 | 0 | 0.000089 ± 0.000127 | 0 | 0 | 0 | 0 | 0 | 0 |
| JAABRT01 | 0.546132 | 0 | 0 | 0 | 0 | 0 | 0 | 0 | 0 | 0.000194 ± 0.000434 | 0 | 0 | 0 | 0 |
| JAADHO01 | 0.150299 | 0 | 0 | 0 | 0 | 0 | 0 | 0 | 0 | 0 | 0.000199 ± 0.000381 | 0.000556 ± 0.001243 | 0 | 0 |
| JAADHY01 | 0.001073 | 0 | 0 | 0.000023 ± 0.000052 | 0.000603 ± 0.001206 | 0 | 0 | 0.001138 ± 0.000501 | 0.000201 ± 0.000402 | 0.000041 ± 0.000092 | 0.000147 ± 0.000328 | 0 | 0 | 0 |
| JAAUSQ01 | 0.000528 | 0 | 0 | 0.000104 ± 0.000232 | 0 | 0.000741 ± 0.000343 | 0 | 0.000252 ± 0.000272 | 0 | 0.000110 ± 0.000172 | 0.000561 ± 0.000403 | 0 | 0.000074 ± 0.000147 | 0.000084 ± 0.000167 |
| JAAYDF01 | 0.610162 | 0 | 0 | 0 | 0 | 0.000103 ± 0.000231 | 0 | 0 | 0 | 0 | 0.000042 ± 0.000094 | 0 | 0 | 0.000027 ± 0.000054 |
| JAAYXS01 | 0.037830 | 0 | 0 | 0 | 0 | 0 | 0 | 0.000081 ± 0.000111 | 0 | 0 | 0 | 0 | 0 | 0 |
| JABDBE01 | 0.598062 | 0.000087 ± 0.000174 | 0 | 0 | 0 | 0 | 0 | 0.000042 ± 0.000095 | 0 | 0.000055 ± 0.000123 | 0 | 0 | 0 | 0 |
| JABDDM01 | 0.510019 | 0 | 0.000059 ± 0.000117 | 0 | 0 | 0 | 0 | 0.000024 ± 0.000054 | 0 | 0 | 0 | 0 | 0 | 0.000063 ± 0.000126 |
| JABDGQ01 | 0.598062 | 0 | 0 | 0 | 0 | 0 | 0 | 0 | 0 | 0.000052 ± 0.000116 | 0.000054 ± 0.000121 | 0 | 0 | 0.000072 ± 0.000143 |
| JABDMH01 | 0.022996 | 0.00007 ± 0.000139 | 0.000623 ± 0.000454 | 0.000116 ± 0.000259 | 0.000201 ± 0.000401 | 0.000977 ± 0.000708 | 0 | 0.000195 ± 0.000306 | 0.00062 ± 0.000959 | 0.000314 ± 0.000299 | 0.000066 ± 0.000148 | 0 | 0 | 0.000382 ± 0.000256 |
| JABFRZ01 | 0.014793 | 0 | 0 | 0 | 0 | 0.000188 ± 0.000264 | 0 | 0.000078 ± 0.000173 | 0 | 0.000414 ± 0.000422 | 0.000419 ± 0.000244 | 0 | 0.000466 ± 0.000574 | 0.000188 ± 0.000377 |
| JABFSC01 | 0.034079 | 0.000551 ± 0.000657 | 0.000726 ± 0.000587 | 0.000658 ± 0.000604 | 0.000361 ± 0.000477 | 0.000066 ± 0.000129 | 0.001034 ± 0.000828 | 0.000238 ± 0.000275 | 0.000316 ± 0.000631 | 0.00168 ± 0.000559 | 0.000065 ± 0.000094 | 0.000131 ± 0.000293 | 0.000439 ± 0.000513 | 0.000403 ± 0.000594 |
| JABSRN01 | 0.454394 | 0 | 0 | 0 | 0 | 0.000130 ± 0.000290 | 0 | 0.000169 ± 0.000379 | 0 | 0.000587 ± 0.000986 | 0 | 0 | 0.000193 ± 0.000386 | 0.000304 ± 0.000607 |
| JABWAY01 | 0.316957 | 0 | 0 | 0 | 0 | 0 | 0 | 0 | 0.000388 ± 0.000775 | 0 | 0 | 0 | 0 | 0 |
| JABWBB01 | 0.000800 | 0 | 0 | 0 | 0 | 0.000405 ± 0.000390 | 0 | 0 | 0 | 0 | 0 | 0 | 0 | 0 |
| JABWCD01 | 0.037830 | 0 | 0 | 0 | 0 | 0 | 0 | 0.000096 ± 0.000132 | 0 | 0 | 0 | 0 | 0 | 0 |
| JACDBH01 | 0.484612 | 0 | 0 | 0 | 0 | 0 | 0 | 0 | 0 | 0.000138 ± 0.000308 | 0 | 0 | 0.001103 ± 0.002205 | 0 |
| JACDCA01 | 0.028409 | 0.019133 ± 0.002133 | 0.015727 ± 0.001112 | 0.010429 ± 0.005731 | 0.017010 ± 0.009836 | 0.013086 ± 0.001694 | 0.014559 ± 0.002102 | 0.007895 ± 0.004234 | 0.0076 ± 0.00954 | 0.016699 ± 0.003683 | 0.01549 ± 0.003807 | 0.009623 ± 0.008822 | 0.006357 ± 0.00864 | 0.018174 ± 0.003399 |
| JACDCH01 | 0.240387 | 0 | 0.000125 ± 0.00025 | 0 | 0 | 0.000198 ± 0.000442 | 0 | 0.000305 ± 0.000514 | 0 | 0.000491 ± 0.000679 | 0.000524 ± 0.000787 | 0 | 0 | 0 |
| JACDCK01 | 0.734567 | 0.000082 ± 0.000164 | 0 | 0.000161 ± 0.00036 | 0.000333 ± 0.000666 | 0 | 0 | 0 | 0 | 0.000055 ± 0.000123 | 0.000088 ± 0.000197 | 0 | 0 | 0 |
| JACDDX01 | 0.001046 | 0.005778 ± 0.001351 | 0.004898 ± 0.001716 | 0.006309 ± 0.001934 | 0.004336 ± 0.002361 | 0.005218 ± 0.001134 | 0.009216 ± 0.000531 | 0.004242 ± 0.00068 | 0.008677 ± 0.001634 | 0.003468 ± 0.001127 | 0.00309 ± 0.001347 | 0.005423 ± 0.002052 | 0.003113 ± 0.002308 | 0.00484 ± 0.000981 |
| JACDFN01 | 0.167926 | 0.000464 ± 0.000616 | 0 | 0.000829 ± 0.001854 | 0.000922 ± 0.001843 | 0 | 0 | 0.000835 ± 0.000858 | 0.000585 ± 0.000706 | 0.000207 ± 0.000463 | 0.00006 ± 0.000135 | 0 | 0 | 0 |
| JACDHG01 | 0.546132 | 0 | 0 | 0 | 0 | 0 | 0 | 0.000078 ± 0.000173 | 0 | 0 | 0 | 0 | 0 | 0 |
| JACDHN01 | 0.692967 | 0 | 0 | 0 | 0 | 0 | 0 | 0.000133 ± 0.000298 | 0 | 0.000157 ± 0.000351 | 0.000126 ± 0.000281 | 0 | 0 | 0 |
| JACEFB01 | 0.002730 | 0 | 0 | 0 | 0 | 0 | 0 | 0.000187 ± 0.000183 | 0.001528 ± 0.001764 | 0.0001 ± 0.000138 | 0.000437 ± 0.000516 | 0 | 0 | 0 |
| JACMME01 | 0.000002 | 0.001422 ± 0.000804 | 0.001895 ± 0.00035 | 0.003635 ± 0.001769 | 0.000452 ± 0.000904 | 0.008981 ± 0.003108 | 0.012447 ± 0.001995 | 0.000361 ± 0.000399 | 0 | 0.000072 ± 0.00016 | 0.003179 ± 0.000465 | 0.002402 ± 0.001525 | 0 | 0.003193 ± 0.001846 |
| JC640 | 0.109107 | 0.000499 ± 0.00041 | 0.000339 ± 0.000679 | 0.000188 ± 0.000421 | 0.000476 ± 0.000615 | 0 | 0.000378 ± 0.00035 | 0 | 0 | 0 | 0.000328 ± 0.000734 | 0.000398 ± 0.000555 | 0 | 0 |
| Jiangella | 0.316957 | 0 | 0 | 0 | 0.001203 ± 0.002407 | 0 | 0 | 0 | 0 | 0 | 0 | 0 | 0 | 0 |
| Jiangellaceae_X | 0.546132 | 0 | 0 | 0 | 0 | 0 | 0 | 0 | 0 | 0.000142 ± 0.000318 | 0 | 0 | 0 | 0 |
| JKG1 | 0.060220 | 0.000416 ± 0.000502 | 0.000412 ± 0.000486 | 0.000516 ± 0.000763 | 0.000289 ± 0.000578 | 0.000461 ± 0.000307 | 0.001906 ± 0.001215 | 0.000755 ± 0.000797 | 0 | 0.000041 ± 0.000092 | 0.000762 ± 0.000726 | 0.010345 ± 0.02196 | 0 | 0.000765 ± 0.001039 |
| Kaistiaceae_X | 0.037830 | 0 | 0 | 0 | 0 | 0 | 0 | 0 | 0 | 0.000472 ± 0.000852 | 0 | 0 | 0 | 0 |
| KBS-96 | 0.005580 | 0 | 0 | 0 | 0.001037 ± 0.001204 | 0 | 0 | 0 | 0 | 0 | 0 | 0 | 0 | 0 |
| Kibdelosporangium | 0.113607 | 0.000461 ± 0.000921 | 0 | 0 | 0 | 0 | 0.000515 ± 0.000705 | 0 | 0 | 0 | 0 | 0 | 0 | 0 |
| Klebsiella | 0.204579 | 0.000339 ± 0.000678 | 0.000656 ± 0.000815 | 0.00117 ± 0.001712 | 0 | 0 | 0.000174 ± 0.00039 | 0 | 0 | 0.00046 ± 0.001028 | 0 | 0.000805 ± 0.000897 | 0 | 0.000289 ± 0.000577 |
| Koribacter | 0.692967 | 0 | 0 | 0 | 0 | 0.000119 ± 0.000265 | 0.000103 ± 0.000231 | 0 | 0 | 0 | 0 | 0.000215 ± 0.00048 | 0 | 0 |
| Kouleothrix | 0.000503 | 0.000149 ± 0.000298 | 0 | 0.001686 ± 0.001292 | 0 | 0.001311 ± 0.00048 | 0.000578 ± 0.000567 | 0.002326 ± 0.000554 | 0.000574 ± 0.001148 | 0.001643 ± 0.000924 | 0.001015 ± 0.001266 | 0 | 0 | 0.000372 ± 0.000744 |
| Kribbella | 0.010219 | 0.00076 ± 0.000896 | 0 | 0.000131 ± 0.000293 | 0 | 0 | 0.000158 ± 0.000353 | 0 | 0.000301 ± 0.000603 | 0.000521 ± 0.000479 | 0.000918 ± 0.000646 | 0 | 0 | 0 |
| Ktedonobacteraceae_X | 0.546132 | 0 | 0 | 0 | 0 | 0 | 0.000175 ± 0.000392 | 0 | 0 | 0 | 0 | 0 | 0 | 0 |
| Labrys | 0.023582 | 0 | 0 | 0 | 0 | 0.000068 ± 0.000152 | 0 | 0 | 0 | 0.000076 ± 0.00017 | 0 | 0.003075 ± 0.005528 | 0 | 0 |
| Lachnospiraceae_X | 0.546132 | 0 | 0 | 0 | 0 | 0 | 0 | 0.000073 ± 0.000163 | 0 | 0 | 0 | 0 | 0 | 0 |
| Lacibacter | 0.005939 | 0 | 0 | 0 | 0 | 0.000318 ± 0.000321 | 0 | 0 | 0 | 0.000294 ± 0.000384 | 0.000083 ± 0.000186 | 0 | 0 | 0 |
| Lacipirellula | 0.001729 | 0.003557 ± 0.000864 | 0.005191 ± 0.002344 | 0.004354 ± 0.0026 | 0.006691 ± 0.00252 | 0.002993 ± 0.001106 | 0.002338 ± 0.001563 | 0.005076 ± 0.001536 | 0.001695 ± 0.001312 | 0.003858 ± 0.000514 | 0.005017 ± 0.000895 | 0.002289 ± 0.002174 | 0.013126 ± 0.007822 | 0.004611 ± 0.001292 |
| Lacunisphaera | 0.247282 | 0 | 0 | 0 | 0 | 0 | 0 | 0.000058 ± 0.00013 | 0 | 0.000682 ± 0.001526 | 0.000178 ± 0.000398 | 0 | 0 | 0.000362 ± 0.000444 |
| Larkinella | 0.316957 | 0 | 0 | 0 | 0 | 0 | 0 | 0 | 0 | 0 | 0 | 0 | 0.000211 ± 0.000423 | 0 |
| LB-PLM-3 | 0.128388 | 0 | 0.000207 ± 0.000413 | 0 | 0 | 0.000233 ± 0.000346 | 0.000044 ± 0.000099 | 0 | 0 | 0.000256 ± 0.000253 | 0.000094 ± 0.00021 | 0 | 0 | 0.000235 ± 0.000271 |
| LHW63021 | 0.484612 | 0 | 0 | 0 | 0 | 0 | 0 | 0 | 0 | 0 | 0 | 0.000161 ± 0.00036 | 0.000276 ± 0.000551 | 0 |
| Limnoglobus | 0.177783 | 0.000191 ± 0.000383 | 0 | 0.000396 ± 0.000659 | 0.001075 ± 0.002151 | 0.000425 ± 0.000437 | 0 | 0.000012 ± 0.000026 | 0.000568 ± 0.000976 | 0.000248 ± 0.000374 | 0.000184 ± 0.000374 | 0.000092 ± 0.000206 | 0.003474 ± 0.002369 | 0.000115 ± 0.000211 |
| Litorilinea | 0.072272 | 0 | 0.000487 ± 0.000974 | 0.000449 ± 0.001004 | 0.000602 ± 0.001205 | 0 | 0 | 0.001435 ± 0.001215 | 0.00094 ± 0.00188 | 0 | 0.000259 ± 0.000578 | 0 | 0.000661 ± 0.000784 | 0 |
| Longimicrobiales_X_X | 0.374830 | 0.000366 ± 0.000732 | 0 | 0 | 0 | 0.000184 ± 0.000412 | 0.000105 ± 0.000235 | 0 | 0 | 0.000261 ± 0.000358 | 0 | 0 | 0 | 0 |
| Longimicrobium | 0.048250 | 0.002053 ± 0.001677 | 0.001343 ± 0.000787 | 0.000352 ± 0.000788 | 0.003482 ± 0.004644 | 0.003659 ± 0.002211 | 0.002134 ± 0.001767 | 0.000657 ± 0.000649 | 0.000718 ± 0.001436 | 0.000956 ± 0.001053 | 0.002866 ± 0.001104 | 0.00179 ± 0.002343 | 0 | 0.003049 ± 0.002866 |
| Luteimonas_C | 0.515778 | 0.000433 ± 0.000867 | 0 | 0 | 0 | 0 | 0 | 0 | 0 | 0 | 0 | 0.000197 ± 0.000439 | 0.000122 ± 0.000244 | 0 |
| Luteitalea | 0.005020 | 0.002279 ± 0.001698 | 0.003071 ± 0.003622 | 0.000743 ± 0.001178 | 0.001897 ± 0.002345 | 0.003011 ± 0.001336 | 0.002134 ± 0.001478 | 0.000725 ± 0.000466 | 0.000991 ± 0.00067 | 0.00405 ± 0.000782 | 0.002637 ± 0.000776 | 0.001822 ± 0.00112 | 0.011714 ± 0.006787 | 0.002647 ± 0.00102 |
| Lysinibacillus | 0.417257 | 0 | 0 | 0 | 0 | 0 | 0.000095 ± 0.000213 | 0.000085 ± 0.00019 | 0 | 0.000269 ± 0.000602 | 0.000217 ± 0.000486 | 0 | 0 | 0.00026 ± 0.000363 |
| Lysobacter | 0.000964 | 0.005727 ± 0.002089 | 0.005247 ± 0.002463 | 0.003528 ± 0.002119 | 0.008078 ± 0.00471 | 0.002571 ± 0.001426 | 0.003341 ± 0.00128 | 0.001358 ± 0.001001 | 0.001599 ± 0.001402 | 0.001951 ± 0.000987 | 0.001922 ± 0.001385 | 0.011144 ± 0.005508 | 0.006646 ± 0.002553 | 0.003207 ± 0.003392 |
| Lysobacter_A | 0.000391 | 0.004037 ± 0.001888 | 0.006271 ± 0.001112 | 0.003509 ± 0.002212 | 0.00501 ± 0.002695 | 0.001515 ± 0.000911 | 0.00176 ± 0.000968 | 0.001414 ± 0.000891 | 0.004147 ± 0.002293 | 0.003102 ± 0.000567 | 0.002681 ± 0.000462 | 0.002494 ± 0.000665 | 0.006551 ± 0.002131 | 0.001942 ± 0.001083 |
| Mal4 | 0.003403 | 0.000183 ± 0.000365 | 0 | 0.0001 ± 0.000224 | 0.000226 ± 0.000452 | 0.000119 ± 0.000265 | 0.00029 ± 0.00041 | 0.001822 ± 0.00068 | 0.000373 ± 0.000746 | 0.000746 ± 0.000497 | 0.000474 ± 0.000437 | 0 | 0.000498 ± 0.000622 | 0.001133 ± 0.000866 |
| Mariniblastus | 0.005572 | 0 | 0.00043 ± 0.000306 | 0.000231 ± 0.000271 | 0 | 0 | 0.000032 ± 0.000071 | 0 | 0 | 0.00015 ± 0.000211 | 0.000121 ± 0.000121 | 0 | 0 | 0 |
| Marmoricola | 0.005580 | 0 | 0.001268 ± 0.002069 | 0 | 0 | 0 | 0 | 0 | 0 | 0 | 0 | 0 | 0 | 0 |
| Massilia | 0.148640 | 0.001221 ± 0.001434 | 0.001468 ± 0.001036 | 0.001266 ± 0.00119 | 0 | 0.001216 ± 0.000754 | 0.001721 ± 0.001004 | 0.000543 ± 0.000535 | 0 | 0.000682 ± 0.000657 | 0.000975 ± 0.000915 | 0.002342 ± 0.002213 | 0.000488 ± 0.000976 | 0.00046 ± 0.000531 |
| MB-PLM-1 | 0.082080 | 0.000296 ± 0.000591 | 0.000752 ± 0.001505 | 0.000193 ± 0.000431 | 0.001675 ± 0.001312 | 0.0011 ± 0.001102 | 0.000708 ± 0.000981 | 0.003044 ± 0.001141 | 0.001048 ± 0.002095 | 0.00046 ± 0.000664 | 0.000883 ± 0.001421 | 0 | 0.000643 ± 0.001286 | 0.000677 ± 0.001353 |
| Mesobacillus | 0.089984 | 0.000684 ± 0.00079 | 0 | 0 | 0 | 0 | 0 | 0.00009 ± 0.000201 | 0 | 0 | 0 | 0 | 0 | 0.000161 ± 0.000323 |
| Mesorhizobium_D | 0.316957 | 0 | 0 | 0 | 0 | 0 | 0 | 0 | 0 | 0 | 0 | 0 | 0.001498 ± 0.002996 | 0 |
| Metabacillus | 0.000186 | 0 | 0.005222 ± 0.001798 | 0.002077 ± 0.001332 | 0.003829 ± 0.003219 | 0.000784 ± 0.000875 | 0.003071 ± 0.001775 | 0.00088 ± 0.00107 | 0.000316 ± 0.000631 | 0.00027 ± 0.000604 | 0.000349 ± 0.000781 | 0 | 0.003162 ± 0.002127 | 0.002811 ± 0.000621 |
| Methylobacterium | 0.598062 | 0.000217 ± 0.000433 | 0 | 0 | 0 | 0.000106 ± 0.000237 | 0 | 0 | 0 | 0 | 0 | 0.000115 ± 0.000256 | 0 | 0 |
| Methylophilaceae_X | 0.040633 | 0.000756 ± 0.000899 | 0.000388 ± 0.000777 | 0.000069 ± 0.000155 | 0.001708 ± 0.002028 | 0.000181 ± 0.000248 | 0.001011 ± 0.000568 | 0.000241 ± 0.000268 | 0 | 0 | 0 | 0 | 0 | 0.000178 ± 0.000217 |
| Methylophilus | 0.362149 | 0 | 0 | 0.00049 ± 0.000487 | 0 | 0.000096 ± 0.000214 | 0.000058 ± 0.000131 | 0.000036 ± 0.000081 | 0.000201 ± 0.000402 | 0 | 0.000154 ± 0.000226 | 0.000215 ± 0.00048 | 0 | 0.000118 ± 0.000237 |
| Methyloversatilis | 0.546132 | 0 | 0 | 0 | 0 | 0 | 0 | 0.000061 ± 0.000136 | 0 | 0 | 0 | 0 | 0 | 0 |
| Microbacteriaceae_X | 0.294382 | 0.000326 ± 0.000416 | 0.000732 ± 0.00115 | 0 | 0 | 0.000263 ± 0.000404 | 0.000152 ± 0.000339 | 0 | 0 | 0.000133 ± 0.000185 | 0.000072 ± 0.000161 | 0.000476 ± 0.001063 | 0 | 0 |
| Microbacterium | 0.010036 | 0 | 0.001958 ± 0.001418 | 0.000769 ± 0.001087 | 0 | 0.000179 ± 0.000401 | 0.000326 ± 0.000728 | 0 | 0 | 0 | 0 | 0 | 0 | 0 |
| Micrococcaceae_X | 0.000000 | 0.002096 ± 0.000751 | 0.021503 ± 0.010682 | 0.037915 ± 0.045132 | 0.005976 ± 0.002986 | 0.014672 ± 0.011005 | 0.011303 ± 0.003487 | 0.000553 ± 0.000602 | 0 | 0.002028 ± 0.000367 | 0.001652 ± 0.000705 | 0.019843 ± 0.008452 | 0 | 0.003373 ± 0.001421 |
| Microlunatus | 0.698475 | 0.000261 ± 0.000522 | 0 | 0 | 0 | 0.000211 ± 0.000288 | 0.000252 ± 0.000564 | 0.000162 ± 0.000363 | 0 | 0.000168 ± 0.000376 | 0.000147 ± 0.000328 | 0 | 0 | 0 |
| Micromonospora | 0.546132 | 0 | 0 | 0 | 0 | 0 | 0.002986 ± 0.006676 | 0 | 0 | 0 | 0 | 0 | 0 | 0 |
| Micromonosporaceae_X | 0.010190 | 0.007095 ± 0.002108 | 0.006091 ± 0.005283 | 0.002296 ± 0.002115 | 0.003484 ± 0.004264 | 0.006752 ± 0.00283 | 0.015541 ± 0.007027 | 0.002828 ± 0.002251 | 0.001033 ± 0.002067 | 0.007323 ± 0.001891 | 0.009029 ± 0.007133 | 0.005611 ± 0.006338 | 0.006161 ± 0.005659 | 0.002095 ± 0.001208 |
| Microtetraspora | 0.546132 | 0 | 0 | 0 | 0 | 0 | 0 | 0 | 0 | 0.000386 ± 0.000862 | 0 | 0 | 0 | 0 |
| Microvirga | 0.000019 | 0.020732 ± 0.001901 | 0.027733 ± 0.002299 | 0.021736 ± 0.005769 | 0.013472 ± 0.001259 | 0.00972 ± 0.002366 | 0.027116 ± 0.004481 | 0.007742 ± 0.001262 | 0.018103 ± 0.007854 | 0.009373 ± 0.003672 | 0.011475 ± 0.001727 | 0.027466 ± 0.01057 | 0.018193 ± 0.006945 | 0.013216 ± 0.005599 |
| Minicystis | 0.692967 | 0 | 0 | 0 | 0 | 0.000059 ± 0.000132 | 0 | 0 | 0 | 0.00017 ± 0.00038 | 0 | 0.000107 ± 0.00024 | 0 | 0 |
| Mizugakiibacter | 0.000800 | 0 | 0 | 0 | 0 | 0 | 0 | 0 | 0 | 0.000413 ± 0.000484 | 0 | 0 | 0 | 0 |
| MM2 | 0.159334 | 0.00149 ± 0.000724 | 0.001206 ± 0.001253 | 0.001433 ± 0.000842 | 0.000997 ± 0.001168 | 0.001017 ± 0.000456 | 0.001012 ± 0.000667 | 0.000452 ± 0.000447 | 0.001053 ± 0.000916 | 0.000898 ± 0.000769 | 0.000263 ± 0.000249 | 0.00133 ± 0.00194 | 0.000334 ± 0.000668 | 0 |
| Mongoliimonas | 0.026334 | 0 | 0.000694 ± 0.000597 | 0 | 0 | 0.000166 ± 0.000228 | 0.000099 ± 0.000222 | 0.000421 ± 0.000538 | 0.000339 ± 0.000679 | 0.000291 ± 0.000267 | 0.000419 ± 0.000441 | 0 | 0 | 0 |
| Moranbacterales_X_X | 0.484612 | 0 | 0 | 0.000232 ± 0.000518 | 0 | 0 | 0 | 0 | 0.000301 ± 0.000603 | 0 | 0 | 0 | 0 | 0 |
| Mycobacteriales_X_X | 0.009020 | 0.000379 ± 0.000759 | 0.000828 ± 0.000984 | 0.000274 ± 0.000377 | 0 | 0.001449 ± 0.00198 | 0.002721 ± 0.003014 | 0 | 0 | 0 | 0.000894 ± 0.001395 | 0 | 0 | 0.00126 ± 0.002521 |
| Mycobacterium | 0.131028 | 0.002833 ± 0.001609 | 0.000245 ± 0.000489 | 0.000157 ± 0.000351 | 0.001222 ± 0.001499 | 0.000482 ± 0.001078 | 0.001088 ± 0.000668 | 0.000366 ± 0.000416 | 0.000373 ± 0.000746 | 0.000488 ± 0.00029 | 0.000202 ± 0.000452 | 0.00076 ± 0.00107 | 0.000873 ± 0.001746 | 0.000181 ± 0.000362 |
| Mycoplana | 0.232381 | 0.000722 ± 0.001444 | 0.002058 ± 0.002921 | 0.000499 ± 0.001116 | 0.000602 ± 0.001203 | 0 | 0.000171 ± 0.000382 | 0.001065 ± 0.001188 | 0 | 0.001796 ± 0.000721 | 0.000383 ± 0.000855 | 0.002506 ± 0.003727 | 0.00156 ± 0.002342 | 0.001155 ± 0.002311 |
| Myxococcaceae_X | 0.598062 | 0 | 0 | 0 | 0 | 0 | 0 | 0.000097 ± 0.000218 | 0 | 0 | 0 | 0.000176 ± 0.000394 | 0.000386 ± 0.000772 | 0 |
| Myxococcales_X_X | 0.546132 | 0 | 0 | 0 | 0 | 0 | 0 | 0.000053 ± 0.000119 | 0 | 0 | 0 | 0 | 0 | 0 |
| Myxococcus | 0.546132 | 0 | 0 | 0 | 0 | 0 | 0 | 0.000058 ± 0.00013 | 0 | 0 | 0 | 0 | 0 | 0 |
| Nannocystaceae_X | 0.076623 | 0.00013 ± 0.000261 | 0 | 0.00023 ± 0.000317 | 0 | 0.000434 ± 0.000453 | 0 | 0.000148 ± 0.000219 | 0 | 0 | 0 | 0 | 0 | 0.000178 ± 0.000355 |
| Nannocystis | 0.534446 | 0.000165 ± 0.000331 | 0 | 0.000131 ± 0.000293 | 0 | 0.000348 ± 0.000627 | 0.000259 ± 0.000363 | 0.000131 ± 0.000192 | 0 | 0.000201 ± 0.000193 | 0.000072 ± 0.000161 | 0 | 0.000426 ± 0.000631 | 0.00017 ± 0.00034 |
| Nevskiaceae_X | 0.546132 | 0 | 0 | 0 | 0 | 0.000165 ± 0.000368 | 0 | 0 | 0 | 0 | 0 | 0 | 0 | 0 |
| Niastella | 0.484223 | 0.00177 ± 0.00087 | 0.00205 ± 0.001821 | 0.000695 ± 0.001057 | 0.001019 ± 0.001719 | 0.001084 ± 0.000661 | 0.000904 ± 0.00058 | 0.001477 ± 0.001562 | 0.000675 ± 0.001349 | 0.000571 ± 0.000199 | 0.000573 ± 0.000555 | 0.003522 ± 0.003999 | 0.000995 ± 0.001581 | 0.000833 ± 0.001001 |
| Nibribacter | 0.484612 | 0 | 0 | 0 | 0 | 0 | 0 | 0.000103 ± 0.00023 | 0 | 0 | 0 | 0 | 0.000202 ± 0.000404 | 0 |
| Nibricoccus | 0.761040 | 0.000325 ± 0.00065 | 0.000295 ± 0.00059 | 0.000193 ± 0.000265 | 0.001015 ± 0.001195 | 0.000181 ± 0.000249 | 0 | 0.000619 ± 0.000701 | 0.00023 ± 0.000459 | 0.000346 ± 0.000373 | 0.000108 ± 0.000242 | 0.000537 ± 0.000736 | 0.000142 ± 0.000285 | 0.000276 ± 0.000326 |
| Nitrosomonas | 0.286451 | 0 | 0 | 0 | 0 | 0 | 0 | 0 | 0 | 0.000034 ± 0.000077 | 0.000114 ± 0.000183 | 0.00012 ± 0.000269 | 0 | 0 |
| Nitrosospira | 0.008759 | 0 | 0.000162 ± 0.000325 | 0.000696 ± 0.000953 | 0 | 0.00052 ± 0.000559 | 0.000107 ± 0.000238 | 0.000323 ± 0.000375 | 0.000273 ± 0.000545 | 0 | 0.000794 ± 0.000637 | 0 | 0 | 0.000912 ± 0.000352 |
| Nitrospira_A | 0.008705 | 0.001138 ± 0.000762 | 0.000597 ± 0.000707 | 0.003119 ± 0.001678 | 0 | 0.000111 ± 0.000247 | 0.000698 ± 0.000478 | 0.000763 ± 0.00071 | 0.000373 ± 0.000746 | 0.000651 ± 0.00055 | 0.000805 ± 0.000335 | 0.000575 ± 0.00079 | 0.000129 ± 0.000257 | 0.000489 ± 0.000572 |
| Nitrospira_C | 0.001367 | 0.007276 ± 0.001678 | 0.006149 ± 0.000673 | 0.005575 ± 0.001419 | 0.006833 ± 0.002119 | 0.004887 ± 0.001189 | 0.003666 ± 0.000824 | 0.005664 ± 0.001109 | 0.010485 ± 0.002053 | 0.004146 ± 0.001084 | 0.004024 ± 0.001435 | 0.0053 ± 0.000973 | 0.004689 ± 0.001484 | 0.006185 ± 0.000194 |
| Nocardia | 0.316957 | 0.000191 ± 0.000383 | 0 | 0 | 0 | 0 | 0 | 0 | 0 | 0 | 0 | 0 | 0 | 0 |
| Nocardioidaceae_X | 0.030213 | 0.002551 ± 0.001078 | 0.000853 ± 0.001062 | 0.001988 ± 0.003555 | 0 | 0.00179 ± 0.001495 | 0.005096 ± 0.001091 | 0.006507 ± 0.004511 | 0.002446 ± 0.004892 | 0.006255 ± 0.00878 | 0.004297 ± 0.003276 | 0.001855 ± 0.00176 | 0.002229 ± 0.002655 | 0.005547 ± 0.004175 |
| Nocardioides | 0.013927 | 0.001371 ± 0.000917 | 0.00188 ± 0.000768 | 0.000324 ± 0.000724 | 0.001075 ± 0.002151 | 0.000895 ± 0.000681 | 0.000158 ± 0.000353 | 0.000351 ± 0.000368 | 0 | 0.00044 ± 0.000603 | 0.001479 ± 0.000835 | 0.001207 ± 0.001368 | 0 | 0.000651 ± 0.000798 |
| Nocardioides_B | 0.192448 | 0 | 0.00135 ± 0.002701 | 0 | 0.000628 ± 0.001256 | 0 | 0.000152 ± 0.00034 | 0.001124 ± 0.001075 | 0 | 0 | 0.00117 ± 0.001697 | 0 | 0.00091 ± 0.001819 | 0 |
| Nonomuraea | 0.020707 | 0.008712 ± 0.010853 | 0 | 0.000941 ± 0.002103 | 0.001714 ± 0.002279 | 0.000274 ± 0.000613 | 0.001597 ± 0.001564 | 0.001038 ± 0.001288 | 0.00333 ± 0.002321 | 0.001043 ± 0.000872 | 0.000646 ± 0.000892 | 0.00019 ± 0.000425 | 0 | 0.000956 ± 0.001111 |
| Nordella | 0.000047 | 0.001285 ± 0.000911 | 0.002324 ± 0.000579 | 0.003149 ± 0.000436 | 0.001755 ± 0.001198 | 0.001468 ± 0.000427 | 0.002405 ± 0.000636 | 0.000989 ± 0.000699 | 0.002846 ± 0.000924 | 0.001468 ± 0.000438 | 0.000701 ± 0.000401 | 0.000222 ± 0.000497 | 0.000183 ± 0.000366 | 0.001063 ± 0.000809 |
| Nostocaceae_X | 0.546132 | 0 | 0 | 0.000647 ± 0.001447 | 0 | 0 | 0 | 0 | 0 | 0 | 0 | 0 | 0 | 0 |
| Noviherbaspirillum | 0.056716 | 0.001212 ± 0.000928 | 0.001184 ± 0.001032 | 0.001508 ± 0.001579 | 0.000163 ± 0.000327 | 0.001579 ± 0.000806 | 0.000732 ± 0.000676 | 0.000438 ± 0.000496 | 0.000209 ± 0.000418 | 0.000132 ± 0.000296 | 0.000679 ± 0.000451 | 0.00094 ± 0.000199 | 0.000773 ± 0.000895 | 0.000803 ± 0.000791 |
| Novosphingopyxis | 0.001754 | 0.003166 ± 0.00163 | 0.006408 ± 0.002088 | 0.007605 ± 0.00311 | 0.010894 ± 0.002157 | 0.004841 ± 0.002454 | 0.00161 ± 0.001184 | 0.007372 ± 0.00417 | 0.002596 ± 0.002122 | 0.003832 ± 0.001696 | 0.003258 ± 0.0021 | 0.00307 ± 0.002705 | 0.005553 ± 0.002015 | 0.001483 ± 0.001924 |
| NP-7 | 0.692967 | 0 | 0 | 0 | 0 | 0.000042 ± 0.000094 | 0 | 0.000024 ± 0.000054 | 0 | 0 | 0.000059 ± 0.000131 | 0 | 0 | 0 |
| OLB13 | 0.874461 | 0 | 0 | 0 | 0.000172 ± 0.000344 | 0.000093 ± 0.000207 | 0 | 0.000042 ± 0.000095 | 0.000029 ± 0.000057 | 0.000039 ± 0.000086 | 0.000058 ± 0.000129 | 0 | 0.000037 ± 0.000074 | 0 |
| OLB17 | 0.001657 | 0.014034 ± 0.00337 | 0.01851 ± 0.001501 | 0.014992 ± 0.005699 | 0.029087 ± 0.0103 | 0.019502 ± 0.004229 | 0.012549 ± 0.002627 | 0.024062 ± 0.002784 | 0.016614 ± 0.007325 | 0.015234 ± 0.001286 | 0.013835 ± 0.003999 | 0.012336 ± 0.004749 | 0.023428 ± 0.006714 | 0.013574 ± 0.0024 |
| OLB7 | 0.114282 | 0.000377 ± 0.000755 | 0.000407 ± 0.000472 | 0.000108 ± 0.000241 | 0.000559 ± 0.001119 | 0.000987 ± 0.000414 | 0.000019 ± 0.000042 | 0.000495 ± 0.000418 | 0 | 0.000405 ± 0.000376 | 0.000416 ± 0.000387 | 0.00071 ± 0.00067 | 0.000295 ± 0.00059 | 0.00017 ± 0.00034 |
| Omnitrophales_X_X | 0.316957 | 0 | 0 | 0 | 0 | 0 | 0 | 0 | 0 | 0 | 0 | 0 | 0 | 0.000186 ± 0.000372 |
| Opitutaceae_X | 0.105161 | 0.000325 ± 0.00065 | 0.000522 ± 0.000636 | 0.000262 ± 0.000585 | 0.000094 ± 0.000143 | 0.000574 ± 0.000368 | 0.001156 ± 0.000801 | 0.000559 ± 0.000839 | 0.000478 ± 0.000744 | 0 | 0.000237 ± 0.000363 | 0.000792 ± 0.000761 | 0 | 0.000468 ± 0.000516 |
| Oscillochloris | 0.546132 | 0 | 0 | 0 | 0 | 0 | 0 | 0 | 0 | 0.000132 ± 0.000296 | 0 | 0 | 0 | 0 |
| P52-10 | 0.004979 | 0.004037 ± 0.001222 | 0.003693 ± 0.002996 | 0.001907 ± 0.001909 | 0.000666 ± 0.001332 | 0.003661 ± 0.00086 | 0.002685 ± 0.000802 | 0.002109 ± 0.001593 | 0.004614 ± 0.003342 | 0.002687 ± 0.000199 | 0.001519 ± 0.001042 | 0.000452 ± 0.001011 | 0 | 0.002283 ± 0.000591 |
| Paenarthrobacter | 0.000104 | 0 | 0.005664 ± 0.011328 | 0.009211 ± 0.0104 | 0 | 0 | 0 | 0 | 0 | 0 | 0 | 0.002775 ± 0.001739 | 0 | 0 |
| Paenibacillaceae_X | 0.114602 | 0 | 0.000254 ± 0.000509 | 0 | 0 | 0 | 0.000412 ± 0.000779 | 0 | 0 | 0.000282 ± 0.000389 | 0 | 0.000074 ± 0.000165 | 0 | 0.000235 ± 0.000471 |
| Paenibacillales_X_X | 0.546132 | 0 | 0 | 0 | 0 | 0 | 0 | 0 | 0 | 0.000138 ± 0.000308 | 0 | 0 | 0 | 0 |
| Paenibacillus_AE | 0.510019 | 0 | 0.000176 ± 0.000352 | 0 | 0 | 0 | 0 | 0.000097 ± 0.000218 | 0 | 0 | 0 | 0 | 0 | 0.000152 ± 0.000304 |
| Paenibacillus_C | 0.099967 | 0 | 0 | 0.000717 ± 0.000746 | 0 | 0 | 0.000217 ± 0.000486 | 0.000257 ± 0.000369 | 0.001526 ± 0.003053 | 0 | 0.00028 ± 0.000387 | 0 | 0 | 0 |
| Paenibacillus_T | 0.316957 | 0 | 0 | 0 | 0 | 0 | 0 | 0 | 0.000976 ± 0.001952 | 0 | 0 | 0 | 0 | 0 |
| Paenirhodobacter | 0.061728 | 0 | 0.000611 ± 0.000718 | 0 | 0 | 0.001037 ± 0.001677 | 0 | 0 | 0 | 0 | 0.000065 ± 0.000145 | 0 | 0 | 0 |
| PALSA-1347 | 0.034103 | 0.000994 ± 0.001175 | 0.000421 ± 0.000507 | 0 | 0.000528 ± 0.001055 | 0.000455 ± 0.001017 | 0.001089 ± 0.001777 | 0 | 0 | 0.001834 ± 0.002136 | 0.001841 ± 0.002191 | 0 | 0 | 0 |
| PALSA-1355_X | 0.734809 | 0 | 0 | 0 | 0 | 0 | 0 | 0.000128 ± 0.000286 | 0.000158 ± 0.000316 | 0.000061 ± 0.000135 | 0.000076 ± 0.000171 | 0 | 0 | 0.000099 ± 0.000197 |
| PALSA-1444 | 0.000009 | 0 | 0 | 0 | 0 | 0 | 0 | 0.000291 ± 0.000229 | 0 | 0 | 0 | 0 | 0 | 0 |
| Palsa-1447 | 0.236184 | 0.000803 ± 0.001011 | 0 | 0.000245 ± 0.000548 | 0.001573 ± 0.001458 | 0.000136 ± 0.000304 | 0.000204 ± 0.000455 | 0.000041 ± 0.000092 | 0 | 0.000403 ± 0.000587 | 0.000145 ± 0.000324 | 0.000603 ± 0.000603 | 0.000447 ± 0.000894 | 0.000594 ± 0.000694 |
| Palsa-739 | 0.037142 | 0.000586 ± 0.000708 | 0.004454 ± 0.003703 | 0.002427 ± 0.001651 | 0.001244 ± 0.002487 | 0.001546 ± 0.001159 | 0.002354 ± 0.00118 | 0.000856 ± 0.001079 | 0.006167 ± 0.004542 | 0.000432 ± 0.000591 | 0.001635 ± 0.002087 | 0.000491 ± 0.000801 | 0 | 0.0019 ± 0.001415 |
| Pan216 | 0.333832 | 0.000139 ± 0.000278 | 0 | 0 | 0 | 0 | 0 | 0.000089 ± 0.000199 | 0 | 0 | 0.000177 ± 0.000247 | 0 | 0 | 0.00011 ± 0.00022 |
| Pan44 | 0.014475 | 0.00353 ± 0.000844 | 0.004432 ± 0.000537 | 0.003816 ± 0.002836 | 0.009237 ± 0.006582 | 0.003653 ± 0.00051 | 0.004402 ± 0.00077 | 0.004221 ± 0.001443 | 0.005826 ± 0.001347 | 0.006433 ± 0.000985 | 0.002963 ± 0.000559 | 0.004055 ± 0.001132 | 0.006635 ± 0.005632 | 0.002801 ± 0.001538 |
| Paracnuella | 0.033921 | 0.000203 ± 0.000406 | 0 | 0 | 0.000572 ± 0.000734 | 0 | 0 | 0 | 0 | 0 | 0 | 0 | 0 | 0 |
| Paracraurococcus | 0.043797 | 0.000461 ± 0.000922 | 0 | 0.000617 ± 0.000922 | 0 | 0.000626 ± 0.001024 | 0.00085 ± 0.000922 | 0 | 0 | 0.0009 ± 0.000535 | 0.000123 ± 0.000274 | 0 | 0 | 0.000203 ± 0.000406 |
| Paraflavitalea | 0.037488 | 0.000217 ± 0.000433 | 0 | 0 | 0 | 0 | 0 | 0 | 0 | 0 | 0 | 0 | 0 | 0.000322 ± 0.000381 |
| Paramesorhizobium | 0.007565 | 0 | 0.011614 ± 0.010391 | 0.004915 ± 0.003144 | 0.006029 ± 0.001715 | 0.002449 ± 0.000916 | 0.001454 ± 0.001374 | 0.00205 ± 0.000815 | 0.007533 ± 0.007919 | 0.002144 ± 0.002466 | 0.004159 ± 0.002296 | 0.003215 ± 0.003953 | 0.000747 ± 0.001493 | 0.00063 ± 0.00126 |
| PCC7113 | 0.546132 | 0 | 0 | 0 | 0 | 0 | 0 | 0.000375 ± 0.000838 | 0 | 0 | 0 | 0 | 0 | 0 |
| Pedosphaera | 0.069777 | 0 | 0 | 0 | 0 | 0 | 0 | 0 | 0 | 0.000145 ± 0.000324 | 0.000143 ± 0.000222 | 0 | 0 | 0.000268 ± 0.0004 |
| Pedosphaerales_X_X | 0.248071 | 0.003868 ± 0.00106 | 0.001787 ± 0.00141 | 0.002012 ± 0.00172 | 0.00367 ± 0.003266 | 0.005818 ± 0.001792 | 0.001985 ± 0.001407 | 0.003501 ± 0.001864 | 0.005066 ± 0.002448 | 0.004399 ± 0.002194 | 0.004047 ± 0.002051 | 0.004232 ± 0.003289 | 0.002864 ± 0.004142 | 0.004084 ± 0.002316 |
| Pelagibius | 0.000800 | 0 | 0 | 0 | 0 | 0 | 0 | 0.000648 ± 0.000737 | 0 | 0 | 0 | 0 | 0 | 0 |
| Pelomonas | 0.618381 | 0 | 0 | 0.000193 ± 0.000431 | 0 | 0 | 0.000456 ± 0.001019 | 0 | 0 | 0 | 0 | 0 | 0 | 0 |
| Peptostreptococcaceae_X | 0.362798 | 0 | 0 | 0 | 0.000768 ± 0.001536 | 0 | 0.000364 ± 0.000518 | 0.000142 ± 0.000318 | 0 | 0 | 0.000095 ± 0.000212 | 0 | 0 | 0 |
| Phenylobacterium | 0.020974 | 0.000886 ± 0.001772 | 0.002479 ± 0.002396 | 0.000162 ± 0.000362 | 0 | 0.001354 ± 0.000362 | 0.000343 ± 0.000494 | 0.000968 ± 0.000638 | 0 | 0.00128 ± 0.000788 | 0.000303 ± 0.000678 | 0.000909 ± 0.001256 | 0.000468 ± 0.000935 | 0 |
| Phragmitibacter | 0.012612 | 0.002435 ± 0.000753 | 0.001177 ± 0.000244 | 0.001275 ± 0.001311 | 0.000565 ± 0.001131 | 0.001116 ± 0.000639 | 0.000592 ± 0.001059 | 0.001806 ± 0.000305 | 0 | 0.001264 ± 0.000741 | 0.000183 ± 0.000318 | 0.000153 ± 0.000343 | 0.002016 ± 0.002875 | 0.000448 ± 0.000897 |
| Phycisphaerae_X_X_X | 0.000254 | 0.000443 ± 0.000886 | 0 | 0.000177 ± 0.000397 | 0.00221 ± 0.001789 | 0.001378 ± 0.001265 | 0.001153 ± 0.000693 | 0.003293 ± 0.001337 | 0.000258 ± 0.000517 | 0.001348 ± 0.000363 | 0.002019 ± 0.000506 | 0 | 0.002131 ± 0.00174 | 0.002421 ± 0.00101 |
| Phycisphaerales_X_X | 0.047304 | 0.000087 ± 0.000174 | 0.000262 ± 0.000303 | 0.000102 ± 0.000228 | 0 | 0.000634 ± 0.000418 | 0.000126 ± 0.000282 | 0.000401 ± 0.000432 | 0.000235 ± 0.00047 | 0.0005 ± 0.000485 | 0.000807 ± 0.000191 | 0.000066 ± 0.000146 | 0.000614 ± 0.00076 | 0.000531 ± 0.000748 |
| Phytohabitans | 0.478997 | 0.002585 ± 0.002774 | 0.005303 ± 0.006248 | 0.003186 ± 0.004409 | 0.001909 ± 0.003819 | 0.000983 ± 0.001565 | 0.004081 ± 0.003501 | 0.003754 ± 0.003271 | 0.001622 ± 0.003244 | 0.000536 ± 0.000794 | 0.001692 ± 0.001808 | 0.001257 ± 0.00281 | 0 | 0.0036 ± 0.004199 |
| Pirellula | 0.002479 | 0.005717 ± 0.001648 | 0.006127 ± 0.003284 | 0.004821 ± 0.001998 | 0.008805 ± 0.003676 | 0.007072 ± 0.003191 | 0.006373 ± 0.000633 | 0.012169 ± 0.002739 | 0.012333 ± 0.005371 | 0.009612 ± 0.003033 | 0.007677 ± 0.000966 | 0.005162 ± 0.00095 | 0.015475 ± 0.005095 | 0.012268 ± 0.005039 |
| Pirellulaceae_X | 0.024576 | 0.002876 ± 0.001246 | 0.005852 ± 0.002137 | 0.002449 ± 0.000726 | 0.006319 ± 0.001715 | 0.003417 ± 0.001262 | 0.003063 ± 0.001085 | 0.005191 ± 0.002461 | 0.002852 ± 0.000649 | 0.005482 ± 0.00167 | 0.005744 ± 0.001716 | 0.003485 ± 0.001142 | 0.004902 ± 0.003661 | 0.003912 ± 0.000602 |
| Pirellulales_X_X | 0.000172 | 0.008074 ± 0.001675 | 0.013508 ± 0.003616 | 0.012322 ± 0.00554 | 0.009696 ± 0.001489 | 0.012227 ± 0.003664 | 0.00932 ± 0.00088 | 0.020174 ± 0.002847 | 0.023673 ± 0.00536 | 0.01662 ± 0.002205 | 0.018662 ± 0.00425 | 0.009488 ± 0.003338 | 0.014568 ± 0.005802 | 0.010817 ± 0.003166 |
| Piscinibacter | 0.055493 | 0 | 0 | 0 | 0 | 0 | 0 | 0.000076 ± 0.000127 | 0 | 0.00021 ± 0.000311 | 0 | 0 | 0 | 0 |
| Pla111 | 0.051762 | 0 | 0 | 0 | 0 | 0.000165 ± 0.000233 | 0 | 0.000396 ± 0.000413 | 0 | 0.00018 ± 0.000273 | 0.000249 ± 0.000557 | 0 | 0 | 0 |
| Pla175 | 0.008365 | 0.000461 ± 0.000922 | 0 | 0 | 0.000377 ± 0.000754 | 0.000105 ± 0.000235 | 0.000204 ± 0.000455 | 0.004696 ± 0.002035 | 0.000373 ± 0.000746 | 0.000255 ± 0.000421 | 0.000539 ± 0.000588 | 0 | 0.000427 ± 0.000854 | 0.000495 ± 0.000731 |
| Planctomyces_A | 0.549796 | 0.000916 ± 0.001264 | 0.000367 ± 0.000476 | 0.000621 ± 0.000613 | 0.000814 ± 0.001032 | 0.000871 ± 0.000644 | 0.000164 ± 0.000247 | 0.000846 ± 0.000605 | 0.000496 ± 0.000992 | 0.001086 ± 0.000658 | 0.001346 ± 0.000991 | 0.001072 ± 0.001669 | 0.001757 ± 0.002741 | 0.000345 ± 0.000452 |
| Planctomycetaceae_X | 0.000023 | 0.010144 ± 0.002463 | 0.004415 ± 0.003311 | 0.003854 ± 0.001273 | 0.005841 ± 0.005876 | 0.003249 ± 0.001262 | 0.00589 ± 0.000714 | 0.02059 ± 0.002635 | 0.009422 ± 0.000914 | 0.009175 ± 0.002836 | 0.010408 ± 0.002036 | 0.00214 ± 0.002082 | 0.017007 ± 0.004685 | 0.007325 ± 0.004512 |
| Planctomycetales_X_X | 0.000218 | 0.000313 ± 0.000626 | 0.000523 ± 0.000438 | 0.001618 ± 0.000392 | 0 | 0.000574 ± 0.000241 | 0.000455 ± 0.000446 | 0.002399 ± 0.000484 | 0.001821 ± 0.001451 | 0.001237 ± 0.000841 | 0.000879 ± 0.000555 | 0 | 0.000092 ± 0.000184 | 0.000627 ± 0.000436 |
| Planctomycetes_X_X_X | 0.003753 | 0.001692 ± 0.001382 | 0.000391 ± 0.000783 | 0.000151 ± 0.000337 | 0.002227 ± 0.002058 | 0.002393 ± 0.000184 | 0.000689 ± 0.000616 | 0.003092 ± 0.001501 | 0.001805 ± 0.00217 | 0.001599 ± 0.000549 | 0.001425 ± 0.000894 | 0 | 0.001039 ± 0.000931 | 0.001577 ± 0.001001 |
| Planctomycetota_X_X_X_X | 0.130251 | 0.000138 ± 0.00018 | 0 | 0 | 0.000109 ± 0.000219 | 0.000384 ± 0.000393 | 0 | 0.000209 ± 0.000207 | 0 | 0.000176 ± 0.000394 | 0.000018 ± 0.00004 | 0.000036 ± 0.00008 | 0 | 0.000086 ± 0.000118 |
| Planococcaceae_X | 0.058725 | 0.00135 ± 0.000407 | 0.000949 ± 0.000909 | 0.000503 ± 0.000981 | 0.000621 ± 0.000725 | 0.000405 ± 0.000591 | 0.002208 ± 0.001122 | 0.000382 ± 0.000636 | 0.000798 ± 0.001595 | 0.000817 ± 0.000988 | 0.000544 ± 0.000526 | 0.000648 ± 0.000739 | 0.001864 ± 0.000285 | 0.000636 ± 0.000381 |
| Planomonospora | 0.190463 | 0.000325 ± 0.00065 | 0.001711 ± 0.002072 | 0.000539 ± 0.001206 | 0 | 0.001299 ± 0.000915 | 0.000407 ± 0.00091 | 0 | 0 | 0.000165 ± 0.00037 | 0.000424 ± 0.000611 | 0.001908 ± 0.002852 | 0 | 0.001815 ± 0.002684 |
| Planosporangium | 0.036421 | 0.002752 ± 0.003722 | 0.001741 ± 0.003481 | 0.000784 ± 0.001754 | 0.00256 ± 0.001921 | 0.004401 ± 0.00186 | 0.00419 ± 0.003165 | 0.000928 ± 0.001366 | 0 | 0.003383 ± 0.000711 | 0.002153 ± 0.002001 | 0.000942 ± 0.002105 | 0.000588 ± 0.001176 | 0.001509 ± 0.001838 |
| PMG-095 | 0.504719 | 0.000244 ± 0.000488 | 0.002014 ± 0.002415 | 0.000312 ± 0.000698 | 0.005424 ± 0.006506 | 0.001052 ± 0.000813 | 0.000487 ± 0.000666 | 0.000668 ± 0.001294 | 0.000388 ± 0.000775 | 0.000863 ± 0.000483 | 0.000275 ± 0.000399 | 0.00107 ± 0.001665 | 0.00168 ± 0.001851 | 0.000743 ± 0.000501 |
| PMNU01 | 0.234531 | 0.000104 ± 0.000209 | 0.000472 ± 0.000553 | 0.000316 ± 0.000378 | 0 | 0.000136 ± 0.000195 | 0.000227 ± 0.000331 | 0.000063 ± 0.000141 | 0.000502 ± 0.001004 | 0.000202 ± 0.000452 | 0.000087 ± 0.000194 | 0.000682 ± 0.001525 | 0 | 0.000708 ± 0.000379 |
| PNKZ01 | 0.055680 | 0.000353 ± 0.000413 | 0.001473 ± 0.000602 | 0.000617 ± 0.00065 | 0.000991 ± 0.001055 | 0.000968 ± 0.000591 | 0.000833 ± 0.000505 | 0.001674 ± 0.000732 | 0.000129 ± 0.000258 | 0.000997 ± 0.000591 | 0.000958 ± 0.000341 | 0.000483 ± 0.000669 | 0.000413 ± 0.000825 | 0.000351 ± 0.000406 |
| Polyangia_X_X_X | 0.598062 | 0 | 0 | 0.000051 ± 0.000114 | 0.000366 ± 0.000731 | 0 | 0 | 0 | 0 | 0.00009 ± 0.000201 | 0 | 0 | 0 | 0 |
| Polyangiaceae_X | 0.002801 | 0 | 0.000127 ± 0.000254 | 0.001875 ± 0.003736 | 0 | 0.002079 ± 0.001029 | 0 | 0.001225 ± 0.001191 | 0.000774 ± 0.000913 | 0.001599 ± 0.001403 | 0.002122 ± 0.000824 | 0.000935 ± 0.002091 | 0.000553 ± 0.000639 | 0.000175 ± 0.000349 |
| Polyangiales_X_X | 0.037760 | 0.00023 ± 0.000461 | 0.001442 ± 0.001036 | 0.000216 ± 0.000482 | 0.002311 ± 0.001589 | 0.000833 ± 0.00034 | 0.000064 ± 0.000144 | 0.000559 ± 0.000541 | 0.000355 ± 0.000711 | 0.000816 ± 0.000477 | 0.000311 ± 0.00031 | 0.001108 ± 0.001214 | 0.001244 ± 0.001318 | 0.000866 ± 0.000713 |
| Polyangium | 0.193829 | 0.004461 ± 0.002632 | 0.004226 ± 0.002974 | 0.000876 ± 0.001215 | 0.000301 ± 0.000603 | 0.000723 ± 0.000687 | 0.001609 ± 0.001473 | 0.001837 ± 0.001489 | 0.000675 ± 0.001349 | 0.0018 ± 0.001179 | 0.000806 ± 0.00088 | 0.002768 ± 0.002902 | 0.001716 ± 0.001993 | 0.00191 ± 0.002552 |
| Pontibacter | 0.000126 | 0.000096 ± 0.000191 | 0.001965 ± 0.000301 | 0.000132 ± 0.000295 | 0.001368 ± 0.001735 | 0.002431 ± 0.000715 | 0.000462 ± 0.000636 | 0.001565 ± 0.001 | 0 | 0.000379 ± 0.00053 | 0.001647 ± 0.000497 | 0 | 0.000138 ± 0.000276 | 0.00056 ± 0.000649 |
| Povalibacter | 0.000082 | 0.014447 ± 0.001226 | 0.009462 ± 0.001584 | 0.005518 ± 0.002305 | 0.008764 ± 0.003417 | 0.012646 ± 0.004022 | 0.008134 ± 0.001478 | 0.007723 ± 0.001711 | 0.012545 ± 0.002858 | 0.018534 ± 0.003617 | 0.011519 ± 0.001958 | 0.013036 ± 0.004274 | 0.016506 ± 0.003804 | 0.01129 ± 0.001202 |
| Priestia | 0.018430 | 0 | 0 | 0.000276 ± 0.000616 | 0 | 0.000171 ± 0.000383 | 0 | 0 | 0 | 0.000948 ± 0.000684 | 0.000147 ± 0.000328 | 0.000299 ± 0.000668 | 0 | 0 |
| Prosthecobacter | 0.232505 | 0.000148 ± 0.000295 | 0 | 0.000952 ± 0.00122 | 0.000756 ± 0.00114 | 0 | 0 | 0.000056 ± 0.000125 | 0 | 0.000164 ± 0.000366 | 0.000094 ± 0.00021 | 0 | 0.000441 ± 0.000882 | 0.000125 ± 0.000249 |
| Providencia | 0.546132 | 0 | 0 | 0 | 0 | 0 | 0 | 0 | 0 | 0 | 0 | 0.001272 ± 0.002845 | 0 | 0 |
| Pseudoduganella | 0.000091 | 0 | 0.00031 ± 0.000419 | 0.006331 ± 0.004113 | 0.000201 ± 0.000402 | 0.00137 ± 0.001022 | 0.000533 ± 0.000501 | 0.000906 ± 0.001505 | 0.000761 ± 0.001521 | 0.002627 ± 0.000499 | 0.000689 ± 0.000989 | 0.005585 ± 0.004856 | 0 | 0.001833 ± 0.001751 |
| Pseudohaliea | 0.006548 | 0 | 0 | 0 | 0 | 0 | 0 | 0 | 0 | 0.000229 ± 0.000224 | 0.000042 ± 0.000094 | 0 | 0 | 0 |
| Pseudomonadaceae_X | 0.067527 | 0.002007 ± 0.002098 | 0.002766 ± 0.002414 | 0.003353 ± 0.001632 | 0.003215 ± 0.002735 | 0.006807 ± 0.005707 | 0.000641 ± 0.000681 | 0.002116 ± 0.000861 | 0.002014 ± 0.001375 | 0.004876 ± 0.004233 | 0.004075 ± 0.003809 | 0.005309 ± 0.00197 | 0.002883 ± 0.002495 | 0.001678 ± 0.001679 |
| Pseudomonas | 0.546132 | 0 | 0 | 0 | 0 | 0 | 0 | 0 | 0 | 0 | 0.000787 ± 0.00176 | 0 | 0 | 0 |
| Pseudomonas_F | 0.077047 | 0.001046 ± 0.001656 | 0.000155 ± 0.00031 | 0.000019 ± 0.000042 | 0.000194 ± 0.000387 | 0.000148 ± 0.000213 | 0 | 0.000019 ± 0.000043 | 0.00047 ± 0.00094 | 0.000849 ± 0.000737 | 0.000357 ± 0.000459 | 0 | 0 | 0 |
| Pseudomonas_M | 0.000020 | 0 | 0.002374 ± 0.000635 | 0.001432 ± 0.002552 | 0.0049 ± 0.00342 | 0.010434 ± 0.012628 | 0 | 0.001971 ± 0.000969 | 0 | 0.000152 ± 0.000339 | 0.000355 ± 0.00065 | 0 | 0 | 0 |
| Pseudomonas_R | 0.316957 | 0 | 0 | 0 | 0 | 0 | 0 | 0 | 0 | 0 | 0 | 0 | 0.00059 ± 0.001179 | 0 |
| Pseudomonas_S | 0.546132 | 0 | 0 | 0 | 0 | 0.000136 ± 0.000304 | 0 | 0 | 0 | 0 | 0 | 0 | 0 | 0 |
| Pseudonocardia | 0.006872 | 0.000522 ± 0.001044 | 0.000856 ± 0.001711 | 0.000643 ± 0.001438 | 0 | 0.000859 ± 0.000843 | 0.001567 ± 0.002177 | 0.001615 ± 0.001345 | 0.000531 ± 0.001062 | 0.001565 ± 0.002144 | 0.006029 ± 0.002697 | 0 | 0.001605 ± 0.001856 | 0.00846 ± 0.010765 |
| Pseudonocardiaceae_X | 0.586564 | 0 | 0 | 0.000247 ± 0.000552 | 0.000754 ± 0.001507 | 0.001484 ± 0.003319 | 0.003571 ± 0.007101 | 0 | 0 | 0 | 0 | 0.000262 ± 0.000586 | 0.000707 ± 0.001415 | 0 |
| Pseudorivibacter | 0.059446 | 0.00063 ± 0.000755 | 0.000274 ± 0.000548 | 0.000127 ± 0.000285 | 0 | 0.00073 ± 0.000485 | 0.000278 ± 0.000411 | 0.000235 ± 0.000525 | 0.002681 ± 0.004164 | 0.001677 ± 0.001025 | 0.000264 ± 0.000365 | 0.000796 ± 0.00178 | 0 | 0.000461 ± 0.000627 |
| Pseudoxanthomonas | 0.000063 | 0.000244 ± 0.000487 | 0 | 0 | 0 | 0.000161 ± 0.000359 | 0 | 0.000075 ± 0.000168 | 0 | 0.006794 ± 0.007856 | 0.001283 ± 0.001457 | 0 | 0.002377 ± 0.000524 | 0 |
| Pseudoxanthomonas_A | 0.008858 | 0 | 0.001146 ± 0.000771 | 0.004768 ± 0.005305 | 0 | 0.001072 ± 0.000968 | 0 | 0.002074 ± 0.000767 | 0 | 0.000322 ± 0.00047 | 0.000905 ± 0.00057 | 0.003637 ± 0.005727 | 0.001305 ± 0.00261 | 0.000924 ± 0.000858 |
| Pyrinomonadaceae_X | 0.006335 | 0.001 ± 0.000544 | 0 | 0.000154 ± 0.000345 | 0 | 0.001154 ± 0.001593 | 0.000271 ± 0.000397 | 0.002617 ± 0.001875 | 0.00149 ± 0.002106 | 0.00326 ± 0.001886 | 0.001043 ± 0.000328 | 0.000359 ± 0.000618 | 0.001589 ± 0.001935 | 0.000353 ± 0.000475 |
| QHBO01 | 0.618381 | 0 | 0 | 0 | 0 | 0 | 0 | 0.000301 ± 0.000672 | 0 | 0.000186 ± 0.000416 | 0 | 0 | 0 | 0 |
| QHVH01 | 0.000005 | 0.000623 ± 0.000498 | 0.000157 ± 0.000313 | 0.000899 ± 0.001036 | 0.006964 ± 0.002407 | 0.003081 ± 0.000708 | 0.000311 ± 0.000301 | 0.00988 ± 0.002612 | 0.000772 ± 0.000892 | 0.002605 ± 0.00075 | 0.001109 ± 0.000714 | 0.000415 ± 0.000631 | 0.003445 ± 0.002357 | 0.002758 ± 0.001027 |
| QHWT01 | 0.000047 | 0.012444 ± 0.004997 | 0.0067 ± 0.006169 | 0.012584 ± 0.004851 | 0.001244 ± 0.002487 | 0.007825 ± 0.002211 | 0.00249 ± 0.001693 | 0.015195 ± 0.002695 | 0.042229 ± 0.017573 | 0.015218 ± 0.007752 | 0.01159 ± 0.002631 | 0.026084 ± 0.011042 | 0.013786 ± 0.006054 | 0.010382 ± 0.003464 |
| QHXM01 | 0.000511 | 0.007259 ± 0.005086 | 0.004537 ± 0.001517 | 0.009582 ± 0.003325 | 0.008763 ± 0.001642 | 0.018705 ± 0.003523 | 0.006167 ± 0.005687 | 0.00826 ± 0.002228 | 0.003732 ± 0.007463 | 0.008431 ± 0.000457 | 0.008163 ± 0.004822 | 0.000782 ± 0.001748 | 0 | 0.011592 ± 0.003424 |
| Qipengyuania | 0.023512 | 0.001662 ± 0.001444 | 0.001122 ± 0.000783 | 0.001454 ± 0.000993 | 0 | 0.000642 ± 0.000639 | 0.000629 ± 0.000963 | 0.000102 ± 0.000227 | 0 | 0.000354 ± 0.000331 | 0.001606 ± 0.000662 | 0.000714 ± 0.001011 | 0.000358 ± 0.000717 | 0.000715 ± 0.000703 |
| QOAZ01 | 0.546132 | 0 | 0 | 0.000362 ± 0.00081 | 0 | 0 | 0 | 0 | 0 | 0 | 0 | 0 | 0 | 0 |
| QQVM01 | 0.618381 | 0 | 0 | 0 | 0 | 0 | 0 | 0 | 0 | 0.000055 ± 0.000123 | 0.000137 ± 0.000307 | 0 | 0 | 0 |
| RBC074 | 0.024952 | 0.00455 ± 0.000629 | 0.003714 ± 0.003087 | 0.003125 ± 0.002613 | 0.006306 ± 0.004676 | 0.005215 ± 0.001371 | 0.001856 ± 0.000814 | 0.004147 ± 0.000501 | 0.005164 ± 0.005568 | 0.001062 ± 0.000917 | 0.004328 ± 0.001054 | 0.002006 ± 0.001855 | 0.003261 ± 0.002951 | 0.004385 ± 0.000176 |
| RBG-16-71-46 | 0.037363 | 0.000252 ± 0.000504 | 0 | 0.000364 ± 0.000502 | 0 | 0.00123 ± 0.001262 | 0 | 0.00072 ± 0.001002 | 0.000558 ± 0.001117 | 0.000579 ± 0.000609 | 0.000925 ± 0.000317 | 0 | 0 | 0.000606 ± 0.000702 |
| RBG-16-71-46_X | 0.019664 | 0.000222 ± 0.000421 | 0.000291 ± 0.000582 | 0.000167 ± 0.000373 | 0 | 0.001343 ± 0.000727 | 0.000199 ± 0.000444 | 0.000803 ± 0.001183 | 0 | 0.000517 ± 0.000725 | 0.000443 ± 0.000396 | 0.000446 ± 0.000626 | 0 | 0.000845 ± 0.000951 |
| REEP01 | 0.037830 | 0 | 0 | 0 | 0 | 0 | 0 | 0.00019 ± 0.000311 | 0 | 0 | 0 | 0 | 0 | 0 |
| Reyranella | 0.305468 | 0.000522 ± 0.001044 | 0 | 0 | 0 | 0.000607 ± 0.001116 | 0.0007 ± 0.000964 | 0.000107 ± 0.000238 | 0 | 0 | 0 | 0 | 0.000322 ± 0.000643 | 0 |
| RGDT01 | 0.000056 | 0.002793 ± 0.000962 | 0 | 0.007036 ± 0.002011 | 0.000327 ± 0.000653 | 0.010658 ± 0.00243 | 0.003894 ± 0.003844 | 0.005425 ± 0.001759 | 0.002159 ± 0.001812 | 0.002769 ± 0.001834 | 0.00461 ± 0.000766 | 0.003743 ± 0.002781 | 0.000778 ± 0.000909 | 0.001755 ± 0.001181 |
| Rhizobiaceae_A_X | 0.643955 | 0 | 0.000153 ± 0.000305 | 0 | 0 | 0 | 0 | 0.000269 ± 0.000601 | 0 | 0 | 0 | 0.004126 ± 0.009225 | 0.000257 ± 0.000515 | 0 |
| Rhizobiaceae_X | 0.096737 | 0.00216 ± 0.000773 | 0.004543 ± 0.002424 | 0.002632 ± 0.002505 | 0.002694 ± 0.003253 | 0.002034 ± 0.000674 | 0.001094 ± 0.00075 | 0.000623 ± 0.000619 | 0.002169 ± 0.002092 | 0.000451 ± 0.000427 | 0.001051 ± 0.000709 | 0.002362 ± 0.002383 | 0.002376 ± 0.00285 | 0.000581 ± 0.000679 |
| Rhizobiales_X_X | 0.158557 | 0.002491 ± 0.002055 | 0.001081 ± 0.001 | 0.000918 ± 0.000836 | 0.002585 ± 0.002511 | 0.00263 ± 0.001556 | 0.002861 ± 0.001821 | 0.002199 ± 0.001833 | 0.001378 ± 0.002756 | 0.000945 ± 0.000511 | 0.001983 ± 0.00103 | 0.000406 ± 0.000908 | 0.000818 ± 0.001636 | 0.002201 ± 0.001483 |
| Rhizobium | 0.006332 | 0.003073 ± 0.001315 | 0.002177 ± 0.000732 | 0.004686 ± 0.000707 | 0.001742 ± 0.001518 | 0.003215 ± 0.001476 | 0.00546 ± 0.003512 | 0.001813 ± 0.002414 | 0.001783 ± 0.00122 | 0.002159 ± 0.00078 | 0.002997 ± 0.001164 | 0.010872 ± 0.008664 | 0.00457 ± 0.00436 | 0.002152 ± 0.001307 |
| Rhodanobacteraceae_X | 0.010243 | 0.000313 ± 0.00046 | 0.001417 ± 0.000707 | 0 | 0 | 0.000081 ± 0.000111 | 0.000226 ± 0.000314 | 0.000075 ± 0.000167 | 0 | 0.000075 ± 0.000111 | 0.000112 ± 0.00017 | 0.001155 ± 0.001214 | 0 | 0 |
| Rhodobacteraceae_X | 0.504697 | 0 | 0 | 0.0001 ± 0.000224 | 0 | 0.000096 ± 0.000216 | 0 | 0.000293 ± 0.000656 | 0 | 0 | 0.000379 ± 0.000525 | 0 | 0.000459 ± 0.000919 | 0 |
| Rhodocyclaceae_X | 0.232500 | 0.001336 ± 0.001808 | 0.002408 ± 0.001707 | 0.000857 ± 0.000993 | 0.00317 ± 0.00185 | 0.001855 ± 0.001165 | 0.00278 ± 0.001862 | 0.001 ± 0.001076 | 0.000174 ± 0.000311 | 0.002015 ± 0.001664 | 0.001216 ± 0.001069 | 0.001307 ± 0.001963 | 0.000894 ± 0.001789 | 0.002278 ± 0.000901 |
| Rhodoligotrophos | 0.546132 | 0 | 0 | 0 | 0 | 0 | 0 | 0.000162 ± 0.000363 | 0 | 0 | 0 | 0 | 0 | 0 |
| Rhodomicrobiaceae_X | 0.064425 | 0.001461 ± 0.001183 | 0.001305 ± 0.001182 | 0 | 0 | 0.000876 ± 0.000981 | 0.000583 ± 0.000636 | 0.000732 ± 0.000791 | 0.000431 ± 0.000861 | 0.000083 ± 0.000185 | 0 | 0.001875 ± 0.002249 | 0.000511 ± 0.001022 | 0.000117 ± 0.000233 |
| Rhodothermaceae_X | 0.618381 | 0 | 0 | 0 | 0 | 0 | 0 | 0 | 0 | 0.000168 ± 0.000377 | 0.000119 ± 0.000266 | 0 | 0 | 0 |
| Rhodothermales_X_X | 0.002662 | 0 | 0 | 0 | 0.001637 ± 0.002582 | 0.000052 ± 0.000115 | 0 | 0.000563 ± 0.000377 | 0 | 0 | 0 | 0 | 0.000138 ± 0.000276 | 0 |
| Risungbinella | 0.493106 | 0.000043 ± 0.000087 | 0 | 0 | 0 | 0 | 0 | 0 | 0 | 0.000099 ± 0.000222 | 0 | 0 | 0 | 0 |
| Robertmurraya | 0.017790 | 0 | 0 | 0.001762 ± 0.00113 | 0.000578 ± 0.001156 | 0.000231 ± 0.000517 | 0.000915 ± 0.001295 | 0 | 0 | 0 | 0.000585 ± 0.000586 | 0 | 0.000648 ± 0.001297 | 0 |
| Roseiflexaceae_X | 0.010173 | 0.000148 ± 0.000296 | 0.000111 ± 0.000221 | 0.000267 ± 0.000266 | 0 | 0.000363 ± 0.000228 | 0.000063 ± 0.000141 | 0.000436 ± 0.000677 | 0 | 0.001099 ± 0.000685 | 0.000757 ± 0.000604 | 0 | 0.000691 ± 0.001382 | 0.000827 ± 0.001184 |
| Roseimicrobium | 0.000390 | 0.005468 ± 0.001858 | 0.00191 ± 0.001448 | 0.002236 ± 0.000797 | 0.001488 ± 0.001047 | 0.000765 ± 0.000477 | 0.003565 ± 0.00169 | 0.001658 ± 0.000439 | 0 | 0.001249 ± 0.000773 | 0.000709 ± 0.00043 | 0.003476 ± 0.001828 | 0.00224 ± 0.001756 | 0.002812 ± 0.001856 |
| Roseomonas_B | 0.025209 | 0 | 0.000236 ± 0.000472 | 0 | 0.00086 ± 0.001721 | 0.000312 ± 0.000308 | 0 | 0.000199 ± 0.000306 | 0 | 0 | 0.000418 ± 0.000289 | 0 | 0.001024 ± 0.001445 | 0 |
| RPRB01 | 0.546132 | 0 | 0 | 0 | 0 | 0 | 0 | 0.000093 ± 0.000208 | 0 | 0 | 0 | 0 | 0 | 0 |
| Rubellimicrobium | 0.202866 | 0.000566 ± 0.000665 | 0.002764 ± 0.002431 | 0.00174 ± 0.002096 | 0.000871 ± 0.001217 | 0.00048 ± 0.000711 | 0.001022 ± 0.001011 | 0.000967 ± 0.000952 | 0 | 0.000387 ± 0.000607 | 0.001327 ± 0.000782 | 0.000898 ± 0.001247 | 0 | 0.000072 ± 0.000143 |
| Rubrivirga | 0.137063 | 0 | 0 | 0 | 0 | 0.00013 ± 0.000187 | 0 | 0.000211 ± 0.000318 | 0 | 0 | 0.000128 ± 0.000177 | 0 | 0 | 0.000057 ± 0.000113 |
| Rubrobacteraceae_X | 0.000568 | 0.007828 ± 0.002388 | 0.008695 ± 0.00273 | 0.008327 ± 0.003057 | 0.007274 ± 0.003182 | 0.007716 ± 0.001371 | 0.012077 ± 0.002926 | 0.004765 ± 0.00106 | 0.006142 ± 0.004703 | 0.005792 ± 0.002157 | 0.008073 ± 0.001917 | 0.004349 ± 0.002622 | 0.000763 ± 0.001525 | 0.012436 ± 0.003839 |
| RZYF01 | 0.618381 | 0 | 0 | 0.000185 ± 0.000414 | 0 | 0 | 0.000076 ± 0.00017 | 0 | 0 | 0 | 0 | 0 | 0 | 0 |
| Saccharimonadaceae_X | 0.002430 | 0.000156 ± 0.000188 | 0.000566 ± 0.000701 | 0.004958 ± 0.004338 | 0.001919 ± 0.001465 | 0.000577 ± 0.001156 | 0.000875 ± 0.001497 | 0.001302 ± 0.000962 | 0.005067 ± 0.004744 | 0.003 ± 0.002129 | 0.014465 ± 0.008213 | 0.000871 ± 0.001446 | 0.002997 ± 0.002996 | 0 |
| Saccharimonadales_X_X | 0.069918 | 0.005486 ± 0.005135 | 0.001149 ± 0.000202 | 0.0011 ± 0.001432 | 0.002954 ± 0.003365 | 0.002027 ± 0.002187 | 0.001013 ± 0.001524 | 0.012211 ± 0.004406 | 0.00175 ± 0.000781 | 0.002479 ± 0.003463 | 0.002902 ± 0.001566 | 0.000685 ± 0.000917 | 0.008579 ± 0.017157 | 0.017974 ± 0.025777 |
| Salmonella | 0.105902 | 0 | 0.000888 ± 0.001775 | 0.000594 ± 0.001328 | 0 | 0 | 0.00156 ± 0.002591 | 0 | 0 | 0 | 0.000135 ± 0.000302 | 0.006415 ± 0.012432 | 0 | 0 |
| Saprospiraceae_X | 0.213585 | 0.0002 ± 0.0004 | 0.000451 ± 0.000524 | 0.000537 ± 0.000342 | 0.000874 ± 0.001022 | 0.000204 ± 0.000189 | 0.000183 ± 0.000188 | 0.000788 ± 0.000563 | 0.000172 ± 0.000344 | 0.000288 ± 0.000272 | 0.00048 ± 0.000211 | 0 | 0.000224 ± 0.000447 | 0.00045 ± 0.000353 |
| SBR1031_X_X | 0.018162 | 0 | 0 | 0 | 0 | 0 | 0 | 0.000318 ± 0.000315 | 0 | 0 | 0.000131 ± 0.000183 | 0 | 0.000122 ± 0.000244 | 0 |
| SCGC-AG-212-J23 | 0.000146 | 0.008877 ± 0.002029 | 0.004497 ± 0.00063 | 0.002558 ± 0.00061 | 0.009419 ± 0.004264 | 0.009347 ± 0.002906 | 0.006081 ± 0.002165 | 0.006326 ± 0.002521 | 0.007114 ± 0.004291 | 0.007848 ± 0.001281 | 0.005906 ± 0.00131 | 0.00584 ± 0.000803 | 0.002029 ± 0.001555 | 0.009793 ± 0.001794 |
| SCMT01 | 0.689800 | 0.000078 ± 0.000157 | 0 | 0.000193 ± 0.000431 | 0 | 0.00013 ± 0.00029 | 0.000126 ± 0.000282 | 0.000072 ± 0.000161 | 0 | 0 | 0.000153 ± 0.000244 | 0 | 0 | 0 |
| SCN-69-37 | 0.000279 | 0.003313 ± 0.000422 | 0.001903 ± 0.001565 | 0 | 0.000477 ± 0.000955 | 0.002989 ± 0.001451 | 0.001473 ± 0.001289 | 0.00544 ± 0.000617 | 0.000933 ± 0.001866 | 0.006083 ± 0.00144 | 0.003164 ± 0.001005 | 0.00164 ± 0.001929 | 0.001683 ± 0.003009 | 0.004048 ± 0.002725 |
| SCSIO-52909 | 0.004068 | 0.001147 ± 0.000844 | 0.002342 ± 0.000964 | 0.002263 ± 0.000553 | 0.004089 ± 0.001472 | 0.002483 ± 0.000656 | 0.003954 ± 0.001375 | 0.002242 ± 0.00092 | 0.000753 ± 0.001505 | 0.002438 ± 0.001185 | 0.003521 ± 0.000682 | 0.002602 ± 0.001659 | 0 | 0.005066 ± 0.003477 |
| SCSIO-58843 | 0.493106 | 0.000209 ± 0.000417 | 0 | 0 | 0 | 0 | 0 | 0 | 0 | 0 | 0.000273 ± 0.000609 | 0 | 0 | 0 |
| SCTI01 | 0.000237 | 0.001427 ± 0.001142 | 0 | 0 | 0.000237 ± 0.000474 | 0.000197 ± 0.00044 | 0 | 0.00494 ± 0.002773 | 0.00984 ± 0.00571 | 0.001271 ± 0.002707 | 0.000455 ± 0.000268 | 0 | 0.00124 ± 0.00248 | 0.001008 ± 0.002016 |
| SCTM01 | 0.001223 | 0 | 0.000466 ± 0.000554 | 0 | 0 | 0.000601 ± 0.000584 | 0 | 0.000866 ± 0.000548 | 0.000388 ± 0.000775 | 0.001868 ± 0.000656 | 0.001023 ± 0.000764 | 0 | 0.000951 ± 0.001182 | 0.000487 ± 0.000464 |
| SCUD01 | 0.003600 | 0.001335 ± 0.000412 | 0.000703 ± 0.000812 | 0.000163 ± 0.000365 | 0.000613 ± 0.000827 | 0.001879 ± 0.000762 | 0.00007 ± 0.000157 | 0.001185 ± 0.001294 | 0.001345 ± 0.001571 | 0.002586 ± 0.000716 | 0.00122 ± 0.000359 | 0.000077 ± 0.000171 | 0.000678 ± 0.001041 | 0.000899 ± 0.00061 |
| SDU3-3 | 0.011315 | 0 | 0 | 0 | 0 | 0.000871 ± 0.000653 | 0.000524 ± 0.001173 | 0 | 0 | 0 | 0.000224 ± 0.0005 | 0.000131 ± 0.000292 | 0 | 0.000204 ± 0.000305 |
| Shimazuella | 0.040503 | 0 | 0 | 0 | 0 | 0.000048 ± 0.000108 | 0 | 0 | 0 | 0 | 0 | 0 | 0 | 0.000187 ± 0.000217 |
| SHUM01 | 0.247809 | 0 | 0 | 0 | 0 | 0.000059 ± 0.000132 | 0 | 0 | 0 | 0.000077 ± 0.000105 | 0 | 0 | 0 | 0.000101 ± 0.000203 |
| SHUZ01 | 0.395833 | 0.000318 ± 0.000408 | 0.00028 ± 0.000561 | 0.000312 ± 0.000435 | 0 | 0.000303 ± 0.000288 | 0.000285 ± 0.000407 | 0.000214 ± 0.000294 | 0.000223 ± 0.000278 | 0.00052 ± 0.000447 | 0.000546 ± 0.000384 | 0.000195 ± 0.000437 | 0 | 0.000099 ± 0.000197 |
| SHVA01 | 0.149578 | 0.000081 ± 0.000163 | 0 | 0 | 0 | 0 | 0 | 0.000258 ± 0.000393 | 0.000753 ± 0.001505 | 0.000274 ± 0.000376 | 0.00056 ± 0.000546 | 0.000166 ± 0.000372 | 0 | 0 |
| SHYK01 | 0.000393 | 0 | 0 | 0 | 0 | 0.000989 ± 0.000663 | 0.000571 ± 0.000539 | 0 | 0 | 0 | 0.000159 ± 0.000355 | 0 | 0 | 0 |
| SIAJ01 | 0.167175 | 0.001165 ± 0.00233 | 0.000812 ± 0.001624 | 0.000944 ± 0.001716 | 0.001644 ± 0.002077 | 0.001333 ± 0.001038 | 0.001461 ± 0.001364 | 0.001575 ± 0.000624 | 0.002211 ± 0.003236 | 0.003749 ± 0.000678 | 0.003356 ± 0.001991 | 0.002367 ± 0.002988 | 0.000873 ± 0.001746 | 0.002691 ± 0.000455 |
| Singulisphaera | 0.618381 | 0 | 0 | 0.000349 ± 0.000779 | 0 | 0.00008 ± 0.00018 | 0 | 0 | 0 | 0 | 0 | 0 | 0 | 0 |
| Siphonobacter | 0.037830 | 0 | 0 | 0 | 0 | 0 | 0 | 0 | 0 | 0.000226 ± 0.000347 | 0 | 0 | 0 | 0 |
| SIRX01 | 0.313671 | 0.000174 ± 0.000348 | 0.000487 ± 0.000974 | 0.000471 ± 0.001053 | 0.000747 ± 0.001495 | 0.000229 ± 0.000512 | 0.001369 ± 0.00137 | 0.000351 ± 0.000786 | 0.001507 ± 0.001107 | 0.000362 ± 0.00052 | 0.000276 ± 0.000617 | 0.001068 ± 0.000977 | 0 | 0 |
| SM1A02_X | 0.015601 | 0 | 0 | 0 | 0 | 0.000274 ± 0.000376 | 0.000075 ± 0.000103 | 0.00074 ± 0.000601 | 0 | 0.000067 ± 0.000151 | 0.000137 ± 0.000188 | 0 | 0 | 0.000136 ± 0.000174 |
| SMWR01 | 0.618381 | 0 | 0 | 0 | 0 | 0.000177 ± 0.000395 | 0 | 0 | 0 | 0.000154 ± 0.000345 | 0 | 0 | 0 | 0 |
| SMWR01_X | 0.104670 | 0.000582 ± 0.000673 | 0 | 0 | 0 | 0.000328 ± 0.000487 | 0 | 0.000502 ± 0.000817 | 0.001228 ± 0.001093 | 0.000107 ± 0.000239 | 0.000396 ± 0.000557 | 0 | 0.000236 ± 0.000472 | 0.000661 ± 0.000855 |
| SMYC01 | 0.000616 | 0.000139 ± 0.000278 | 0 | 0 | 0 | 0 | 0 | 0.000861 ± 0.000276 | 0 | 0.000055 ± 0.000123 | 0.000227 ± 0.00033 | 0 | 0.000098 ± 0.000195 | 0.000107 ± 0.000124 |
| Solirubrobacter | 0.070171 | 0.001212 ± 0.000985 | 0.001392 ± 0.001645 | 0.002971 ± 0.002491 | 0 | 0.001679 ± 0.00111 | 0.00344 ± 0.001938 | 0.000889 ± 0.001221 | 0.00183 ± 0.002326 | 0.002787 ± 0.00174 | 0.001504 ± 0.001274 | 0.001844 ± 0.001781 | 0 | 0.001832 ± 0.001956 |
| Solirubrobacteraceae_X | 0.066945 | 0.002375 ± 0.000982 | 0.00214 ± 0.002993 | 0.003426 ± 0.004193 | 0.003959 ± 0.005625 | 0.001992 ± 0.001524 | 0.002384 ± 0.001991 | 0.00553 ± 0.002444 | 0.00542 ± 0.003025 | 0.00371 ± 0.002164 | 0.004471 ± 0.002045 | 0.001755 ± 0.001693 | 0.000732 ± 0.001464 | 0.002177 ± 0.001653 |
| Solirubrobacterales_X_X | 0.541858 | 0 | 0.00047 ± 0.000939 | 0.001311 ± 0.001812 | 0 | 0.00051 ± 0.000748 | 0 | 0.000058 ± 0.00013 | 0.001777 ± 0.003554 | 0 | 0.000119 ± 0.000266 | 0 | 0 | 0.00071 ± 0.001421 |
| Solitalea | 0.113607 | 0 | 0 | 0 | 0 | 0 | 0 | 0 | 0 | 0.000353 ± 0.000589 | 0 | 0 | 0.000432 ± 0.000865 | 0 |
| Sorangium | 0.635594 | 0 | 0.000382 ± 0.000763 | 0.000414 ± 0.000927 | 0.00087 ± 0.001741 | 0.000372 ± 0.000351 | 0.000605 ± 0.000895 | 0.001882 ± 0.003659 | 0 | 0.000361 ± 0.000496 | 0.0009 ± 0.000772 | 0.000131 ± 0.000293 | 0.000395 ± 0.00079 | 0.000423 ± 0.000536 |
| Sphaerobacter | 0.316957 | 0 | 0 | 0 | 0.000946 ± 0.001893 | 0 | 0 | 0 | 0 | 0 | 0 | 0 | 0 | 0 |
| Sphingobacteriaceae_X | 0.143910 | 0 | 0.000591 ± 0.000732 | 0.000393 ± 0.000879 | 0 | 0 | 0.000152 ± 0.000339 | 0 | 0 | 0 | 0 | 0.00044 ± 0.000634 | 0 | 0 |
| Sphingobacterium | 0.001524 | 0 | 0.001209 ± 0.002098 | 0.010667 ± 0.011762 | 0.00906 ± 0.01812 | 0 | 0.004782 ± 0.010384 | 0 | 0.003772 ± 0.004383 | 0 | 0.000275 ± 0.000399 | 0.014622 ± 0.014071 | 0 | 0 |
| Sphingobium | 0.217684 | 0.000197 ± 0.000394 | 0 | 0 | 0 | 0 | 0 | 0.000268 ± 0.000413 | 0 | 0.000075 ± 0.00017 | 0 | 0 | 0 | 0 |
| Sphingobium_A | 0.003413 | 0.010145 ± 0.001169 | 0.011318 ± 0.002107 | 0.006273 ± 0.00304 | 0.010949 ± 0.001708 | 0.008799 ± 0.000574 | 0.009567 ± 0.00284 | 0.005806 ± 0.001965 | 0.005947 ± 0.002519 | 0.007075 ± 0.00146 | 0.005615 ± 0.000567 | 0.005762 ± 0.003179 | 0.008205 ± 0.00364 | 0.006943 ± 0.001996 |
| Sphingomicrobium | 0.000040 | 0.027112 ± 0.001949 | 0.033046 ± 0.004563 | 0.031731 ± 0.009903 | 0.03044 ± 0.006355 | 0.017483 ± 0.001398 | 0.022128 ± 0.003285 | 0.020254 ± 0.003831 | 0.0295 ± 0.004889 | 0.019785 ± 0.000857 | 0.016225 ± 0.004372 | 0.024378 ± 0.003541 | 0.018474 ± 0.007177 | 0.013646 ± 0.001056 |
| Sphingomonadaceae_X | 0.000677 | 0.044461 ± 0.003084 | 0.057401 ± 0.009951 | 0.048744 ± 0.016333 | 0.04995 ± 0.016679 | 0.031019 ± 0.004568 | 0.042588 ± 0.009873 | 0.02685 ± 0.003951 | 0.02774 ± 0.008389 | 0.035169 ± 0.015524 | 0.023622 ± 0.003217 | 0.036526 ± 0.009376 | 0.037432 ± 0.008902 | 0.027446 ± 0.002945 |
| Sphingomonas_G | 0.000872 | 0 | 0 | 0.000446 ± 0.000616 | 0 | 0.000198 ± 0.000378 | 0.00049 ± 0.00029 | 0 | 0 | 0 | 0 | 0.000131 ± 0.000293 | 0 | 0 |
| Sphingomonas_H | 0.000207 | 0.000217 ± 0.000435 | 0 | 0.000822 ± 0.000781 | 0.000384 ± 0.000768 | 0.000834 ± 0.000184 | 0.001126 ± 0.000341 | 0 | 0 | 0 | 0 | 0.000369 ± 0.000575 | 0 | 0.000489 ± 0.00007 |
| Sphingomonas_K | 0.546132 | 0 | 0 | 0 | 0 | 0 | 0.000152 ± 0.000339 | 0 | 0 | 0 | 0 | 0 | 0 | 0 |
| Spirillospora | 0.008122 | 0.000884 ± 0.001086 | 0 | 0 | 0 | 0 | 0 | 0 | 0 | 0 | 0 | 0 | 0 | 0.00076 ± 0.00115 |
| Sporosarcina | 0.546132 | 0 | 0 | 0 | 0 | 0 | 0.000204 ± 0.000455 | 0 | 0 | 0 | 0 | 0 | 0 | 0 |
| Stappiaceae_X | 0.037830 | 0 | 0 | 0 | 0 | 0 | 0 | 0.000169 ± 0.00024 | 0 | 0 | 0 | 0 | 0 | 0 |
| Starkeya | 0.037830 | 0 | 0 | 0 | 0 | 0 | 0 | 0 | 0 | 0.000146 ± 0.000209 | 0 | 0 | 0 | 0 |
| Stellaceae_X | 0.037830 | 0 | 0 | 0 | 0 | 0 | 0 | 0.000176 ± 0.000244 | 0 | 0 | 0 | 0 | 0 | 0 |
| Stenotrophomonas | 0.004846 | 0 | 0.000098 ± 0.000196 | 0.000839 ± 0.001235 | 0.001054 ± 0.002108 | 0.000087 ± 0.000194 | 0.006023 ± 0.011245 | 0.000109 ± 0.000244 | 0 | 0.000246 ± 0.000345 | 0.000675 ± 0.0005 | 0.008182 ± 0.004235 | 0.001075 ± 0.00215 | 0.000042 ± 0.000085 |
| Steroidobacter | 0.063217 | 0.000333 ± 0.000385 | 0.000398 ± 0.000797 | 0 | 0.001178 ± 0.002355 | 0 | 0.000401 ± 0.000367 | 0.000169 ± 0.000249 | 0 | 0 | 0 | 0 | 0 | 0 |
| Steroidobacter_A | 0.005484 | 0.005183 ± 0.00143 | 0.003893 ± 0.000993 | 0.003585 ± 0.000823 | 0.005014 ± 0.000583 | 0.002688 ± 0.000585 | 0.003507 ± 0.001365 | 0.002738 ± 0.00098 | 0.003409 ± 0.002384 | 0.002207 ± 0.000443 | 0.002086 ± 0.0008 | 0.005007 ± 0.001448 | 0.005667 ± 0.003904 | 0.003661 ± 0.00146 |
| Steroidobacteraceae_X | 0.006006 | 0.002567 ± 0.001835 | 0.003335 ± 0.002786 | 0.001198 ± 0.001291 | 0.0027 ± 0.002043 | 0.006958 ± 0.002203 | 0.0061 ± 0.001237 | 0.004252 ± 0.001843 | 0.002004 ± 0.002556 | 0.001638 ± 0.001702 | 0.002727 ± 0.001921 | 0.001128 ± 0.002476 | 0.001338 ± 0.001441 | 0.001711 ± 0.0013 |
| Streptomyces | 0.090091 | 0.002412 ± 0.001771 | 0.003099 ± 0.00369 | 0.000571 ± 0.001276 | 0.008242 ± 0.006632 | 0.002801 ± 0.001128 | 0.002255 ± 0.001521 | 0.000373 ± 0.000537 | 0.002295 ± 0.003272 | 0.001546 ± 0.00096 | 0.000604 ± 0.000556 | 0.002878 ± 0.001709 | 0.000542 ± 0.001084 | 0.00259 ± 0.003753 |
| Streptomyces_A | 0.001484 | 0.006311 ± 0.003293 | 0.001469 ± 0.001701 | 0 | 0.000528 ± 0.001055 | 0.000681 ± 0.00094 | 0.003305 ± 0.000604 | 0 | 0.000957 ± 0.001914 | 0 | 0.000565 ± 0.000813 | 0.003325 ± 0.00468 | 0.002083 ± 0.002447 | 0.0043 ± 0.0028 |
| Streptomycetaceae_X | 0.991999 | 0.001701 ± 0.002287 | 0.002664 ± 0.001168 | 0.003283 ± 0.002012 | 0.002509 ± 0.002981 | 0.00292 ± 0.002845 | 0.00302 ± 0.002024 | 0.003025 ± 0.000798 | 0.004292 ± 0.006257 | 0.004016 ± 0.002316 | 0.002956 ± 0.002571 | 0.003474 ± 0.004614 | 0.003614 ± 0.003377 | 0.002204 ± 0.001881 |
| Streptosporangiaceae_X | 0.059344 | 0.00188 ± 0.001267 | 0.000588 ± 0.000705 | 0.000184 ± 0.000411 | 0.001946 ± 0.003891 | 0.001338 ± 0.000398 | 0.000999 ± 0.001118 | 0.000868 ± 0.001269 | 0.000187 ± 0.000373 | 0.000265 ± 0.00028 | 0.000063 ± 0.000141 | 0.003089 ± 0.004179 | 0.002201 ± 0.001477 | 0.001622 ± 0.00115 |
| Sulfurifustaceae_X | 0.000401 | 0 | 0 | 0 | 0 | 0 | 0 | 0 | 0.000856 ± 0.000606 | 0 | 0 | 0 | 0 | 0.000068 ± 0.000135 |
| SXKJ01 | 0.130613 | 0 | 0 | 0 | 0 | 0 | 0 | 0.000191 ± 0.000297 | 0 | 0.000039 ± 0.000086 | 0 | 0 | 0 | 0 |
| Symbiobacterium | 0.007570 | 0 | 0 | 0 | 0 | 0 | 0 | 0.000849 ± 0.000965 | 0 | 0.000233 ± 0.00052 | 0 | 0 | 0 | 0 |
| SYSU-D60007 | 0.000069 | 0.000357 ± 0.000713 | 0 | 0.000376 ± 0.000553 | 0 | 0.000656 ± 0.000667 | 0.000171 ± 0.000237 | 0.001835 ± 0.000592 | 0 | 0.000633 ± 0.000393 | 0.000121 ± 0.00027 | 0 | 0.003738 ± 0.00104 | 0 |
| SYSU-D60009 | 0.001669 | 0.005541 ± 0.00209 | 0.006243 ± 0.001587 | 0.002044 ± 0.002106 | 0.004062 ± 0.001771 | 0.00737 ± 0.001714 | 0.004648 ± 0.001179 | 0.003731 ± 0.001087 | 0.007632 ± 0.003684 | 0.006777 ± 0.000801 | 0.004841 ± 0.001482 | 0.002051 ± 0.001279 | 0.004564 ± 0.003744 | 0.0056 ± 0.001296 |
| SYSU-D60014 | 0.001683 | 0.012104 ± 0.000895 | 0.008432 ± 0.001566 | 0.007517 ± 0.002565 | 0.009356 ± 0.005072 | 0.006969 ± 0.001524 | 0.009866 ± 0.001735 | 0.006966 ± 0.001962 | 0.011624 ± 0.00048 | 0.006607 ± 0.001904 | 0.007281 ± 0.001233 | 0.008339 ± 0.001596 | 0.008194 ± 0.002491 | 0.012461 ± 0.002112 |
| SYSU-D60015 | 0.277885 | 0.000451 ± 0.000303 | 0.000251 ± 0.000502 | 0.000195 ± 0.000276 | 0 | 0.000581 ± 0.000716 | 0.000276 ± 0.000347 | 0.000162 ± 0.000238 | 0 | 0.000359 ± 0.000217 | 0.000225 ± 0.000385 | 0 | 0.000491 ± 0.000983 | 0.000329 ± 0.000383 |
| SZUA-115 | 0.264374 | 0.003563 ± 0.001839 | 0.001993 ± 0.001394 | 0.003423 ±  0.0023 | 0.001929 ± 0.001482 | 0.002381 ± 0.001291 | 0.001834 ±  0.0012 | 0.004014 ±  0.00196 | 0.005081 ± 0.003756 | 0.002507 ±  0.00097 | 0.000741 ±  0.00085 | 0.002406 ± 0.001676 | 0.001537 ± 0.002521 | 0.001925 ±  0.00209 |
| SZUA-217 | 0.000149 | 0 | 0 | 0 | 0.002527 ± 0.003671 | 0 | 0 | 0.001867 ± 0.001288 | 0 | 0 | 0 | 0 | 0 | 0 |
| SZUA-252 | 0.001800 | 0.000066 ± 0.000131 | 0.000097 ± 0.000194 | 0 | 0 | 0.000723 ± 0.000401 | 0 | 0.000522 ± 0.000519 | 0.000146 ± 0.000293 | 0.00016  ± 0.000227 | 0.000267 ± 0.000189 | 0 | 0 | 0.000336 ± 0.000379 |
| SZUA-318 | 0.006548 | 0 | 0 | 0 | 0 | 0 | 0 | 0.000369 ± 0.000339 | 0 | 0.000116 ± 0.000259 | 0 | 0 | 0 | 0 |
| SZUA-320 | 0.000009 | 0 | 0 | 0 | 0 | 0 | 0 | 0.000825 ±  0.00057 | 0 | 0 | 0 | 0 | 0 | 0 |
| SZUA-336_X | 0.314502 | 0.000061 ± 0.000122 | 0 | 0 | 0 | 0.000068 ± 0.000152 | 0 | 0 | 0 | 0.000199 ±  0.0003 | 0.000121 ± 0.000166 | 0 | 0 | 0.000106 ± 0.000213 |
| SZUA-42 | 0.316957 | 0 | 0 | 0 | 0 | 0 | 0 | 0 | 0 | 0 | 0 | 0 | 0.000799 ± 0.001599 | 0 |
| Tahibacter | 0.039897 | 0 | 0 | 0 | 0 | 0.000212 ± 0.000474 | 0 | 0 | 0 | 0.000356 ± 0.000362 | 0.000123 ± 0.000274 | 0 | 0 | 0 |
| Tardibacter | 0.546132 | 0 | 0 | 0 | 0 | 0 | 0 | 0.000221 ± 0.000495 | 0 | 0 | 0 | 0 | 0 | 0 |
| Tautonia | 0.150299 | 0 | 0 | 0 | 0 | 0 | 0.000688 ± 0.001539 | 0 | 0 | 0 | 0.000536 ±  0.00083 | 0 | 0 | 0 |
| Terribacillus | 0.546132 | 0 | 0 | 0 | 0 | 0 | 0 | 0 | 0 | 0.000143 ±  0.00032 | 0 | 0 | 0 | 0 |
| Terricaulis | 0.023255 | 0 | 0 | 0.000245 ± 0.000548 | 0.00137  ± 0.001605 | 0.000225 ± 0.000503 | 0.000099 ± 0.000222 | 0.000867 ± 0.000847 | 0.001655 ± 0.000563 | 0 | 0 | 0.000213 ± 0.000476 | 0.001185 ± 0.002371 | 0.000529 ± 0.000667 |
| Terrimicrobium | 0.286655 | 0.001167 ± 0.001004 | 0.002258 ± 0.002001 | 0.000718 ± 0.000861 | 0.000512 ± 0.001024 | 0.000871 ± 0.001473 | 0.00027  ± 0.000266 | 0.001587 ± 0.000375 | 0.000679 ± 0.001358 | 0.001259 ± 0.000798 | 0.000859 ± 0.000873 | 0.000761 ± 0.001701 | 0.002593 ± 0.001864 | 0.000592 ± 0.000901 |
| Terrimonas | 0.049316 | 0 | 0 | 0.000131 ± 0.000293 | 0.00044  ± 0.000879 | 0 | 0 | 0 | 0 | 0.000871 ±  0.0009 | 0 | 0.000978 ± 0.001613 | 0 | 0 |
| Thermoactinomyces_A | 0.546132 | 0 | 0 | 0 | 0 | 0 | 0 | 0.000058 ± 0.00013 | 0 | 0 | 0 | 0 | 0 | 0 |
| Thermoactinomycetaceae_X | 0.546132 | 0 | 0 | 0 | 0 | 0 | 0 | 0.000068 ± 0.000152 | 0 | 0 | 0 | 0 | 0 | 0 |
| Thermomicrobiales_X_X | 0.040503 | 0 | 0 | 0 | 0.001441 ± 0.002028 | 0 | 0 | 0.000114 ± 0.000256 | 0 | 0 | 0 | 0 | 0 | 0 |
| Thermomonas | 0.546132 | 0 | 0 | 0 | 0 | 0 | 0 | 0 | 0 | 0 | 0.000173 ± 0.000387 | 0 | 0 | 0 |
| Thermomonospora | 0.484612 | 0 | 0 | 0 | 0.001183 ± 0.002366 | 0.000429 ±  0.00096 | 0 | 0 | 0 | 0 | 0 | 0 | 0 | 0 |
| Tumebacillales_X_X | 0.044643 | 0 | 0.000111 ± 0.000222 | 0 | 0 | 0.000125 ± 0.00028 | 0 | 0.000937 ± 0.000853 | 0.000646 ± 0.001292 | 0 | 0.000131 ± 0.000292 | 0 | 0.000447 ± 0.000894 | 0.000609 ± 0.000834 |
| Tumebacillus_A | 0.028647 | 0.000148 ± 0.000295 | 0 | 0.000854 ± 0.001323 | 0 | 0 | 0.000371 ± 0.000514 | 0.001692 ± 0.001719 | 0 | 0.000714 ± 0.000659 | 0.001286 ± 0.001011 | 0.000335 ± 0.000549 | 0 | 0 |
| Turicibacter | 0.546132 | 0 | 0 | 0 | 0 | 0.000288 ± 0.000645 | 0 | 0 | 0 | 0 | 0 | 0 | 0 | 0 |
| UBA1020 | 0.546132 | 0 | 0 | 0 | 0 | 0 | 0 | 0 | 0 | 0 | 0.000217 ± 0.000484 | 0 | 0 | 0 |
| UBA10212_X | 0.013764 | 0 | 0 | 0 | 0 | 0.00131  ± 0.001776 | 0.000132 ± 0.000196 | 0 | 0 | 0.000116 ±  0.00026 | 0.000131 ± 0.000183 | 0 | 0 | 0.000566 ± 0.000612 |
| UBA10450_X | 0.000902 | 0.006077 ± 0.002287 | 0.010522 ± 0.004733 | 0.004319 ± 0.002737 | 0.012551 ± 0.005493 | 0.006793 ± 0.002645 | 0.005494 ± 0.002759 | 0.004121 ± 0.001353 | 0.004029 ± 0.003844 | 0.007595 ± 0.002015 | 0.010177 ±  0.00383 | 0.010063 ±  0.00369 | 0.017441 ± 0.004657 | 0.004012 ± 0.003923 |
| UBA10511 | 0.001586 | 0.000854 ± 0.000699 | 0.002121 ± 0.000516 | 0.001799 ± 0.001185 | 0.002836 ± 0.001171 | 0.002309 ±  0.00097 | 0.001139 ± 0.000778 | 0.005948 ± 0.001036 | 0.002137 ± 0.002782 | 0.002986 ± 0.000933 | 0.002474 ±  0.00084 | 0.001118 ± 0.000826 | 0.003123 ± 0.001049 | 0.001326 ± 0.001058 |
| UBA1161_X | 0.000275 | 0.022078 ± 0.001824 | 0.026619 ± 0.006315 | 0.023701 ± 0.006931 | 0.025808 ± 0.011908 | 0.028581 ± 0.003057 | 0.034529 ± 0.006246 | 0.013292 ± 0.002472 | 0.010936 ± 0.008107 | 0.020817 ± 0.002573 | 0.029864 ±  0.00263 | 0.030669 ± 0.008137 | 0.035699 ± 0.005226 | 0.028791 ± 0.000977 |
| UBA11704 | 0.598062 | 0 | 0 | 0.000131 ± 0.000293 | 0.000438 ± 0.000875 | 0 | 0 | 0 | 0 | 0.00005  ± 0.000111 | 0 | 0 | 0 | 0 |
| UBA11741 | 0.031608 | 0.000459 ± 0.000919 | 0.001328 ± 0.002655 | 0.000148 ± 0.000204 | 0.008123 ± 0.006061 | 0.00269  ± 0.000855 | 0.001461 ± 0.002021 | 0.001131 ±  0.00253 | 0.000115 ±  0.00023 | 0.003103 ±  0.00312 | 0.001032 ± 0.001424 | 0.001104 ± 0.002468 | 0 | 0.000628 ± 0.001255 |
| UBA12294 | 0.304464 | 0.000066 ± 0.000131 | 0.000089 ± 0.000177 | 0.000111 ± 0.000248 | 0 | 0 | 0.000123 ± 0.000169 | 0.000175 ± 0.000285 | 0 | 0 | 0 | 0 | 0 | 0.000093 ± 0.000112 |
| UBA12499 | 0.618381 | 0 | 0 | 0.000093 ± 0.000207 | 0 | 0 | 0 | 0 | 0 | 0 | 0.000078 ± 0.000175 | 0 | 0 | 0 |
| UBA1315 | 0.001170 | 0.000864 ±  0.00106 | 0.004023 ± 0.004033 | 0.000548 ± 0.000794 | 0.000151 ± 0.000301 | 0.001696 ± 0.001842 | 0.000998 ±  0.00092 | 0.000251 ± 0.000343 | 0.000439 ± 0.000878 | 0.001354 ± 0.000775 | 0.003602 ± 0.001193 | 0.005279 ± 0.003177 | 0.004355 ± 0.004061 | 0.000401 ± 0.000482 |
| UBA1568 | 0.061471 | 0.00035  ± 0.000466 | 0.00054  ± 0.000644 | 0.000046 ± 0.000103 | 0 | 0 | 0.000155 ± 0.000203 | 0.000396 ± 0.000387 | 0.000188 ± 0.000376 | 0.000409 ± 0.000283 | 0.00014  ±  0.00025 | 0 | 0 | 0.000384 ± 0.000493 |
| UBA1845_X_X | 0.000001 | 0 | 0 | 0 | 0 | 0 | 0 | 0.000747 ±  0.00051 | 0 | 0 | 0.000054 ± 0.000121 | 0 | 0 | 0 |
| UBA2386 | 0.048691 | 0.000213 ± 0.000427 | 0.000372 ± 0.000744 | 0.000211 ± 0.000473 | 0.001166 ± 0.000973 | 0.000546 ± 0.000552 | 0.00029  ± 0.000274 | 0.001542 ± 0.001222 | 0.000301 ± 0.000603 | 0.001056 ± 0.000812 | 0.001202 ± 0.000763 | 0.000511 ± 0.000911 | 0.000129 ± 0.000257 | 0.000159 ± 0.000319 |
| UBA2421 | 0.000027 | 0.015492 ± 0.002592 | 0.017699 ± 0.006722 | 0.009158 ± 0.003997 | 0.020638 ± 0.007041 | 0.025612 ± 0.004194 | 0.011057 ± 0.002903 | 0.023671 ± 0.002006 | 0.015388 ±  0.00293 | 0.018423 ± 0.002148 | 0.025763 ± 0.001236 | 0.014792 ± 0.004816 | 0.031841 ± 0.003574 | 0.015345 ± 0.003499 |
| UBA2475 | 0.194723 | 0.000312 ± 0.000623 | 0 | 0 | 0.000452 ± 0.000903 | 0.000441 ± 0.000664 | 0.000268 ± 0.000403 | 0.000226 ± 0.000244 | 0 | 0.000522 ± 0.000537 | 0 | 0.000297 ± 0.000452 | 0 | 0 |
| UBA2774_X_X | 0.257091 | 0 | 0.000362 ± 0.000421 | 0 | 0.000151 ± 0.000301 | 0.000067 ± 0.000151 | 0.000088 ± 0.000198 | 0.00017  ± 0.000259 | 0 | 0 | 0.000128 ± 0.000176 | 0 | 0 | 0 |
| UBA2982 | 0.000650 | 0.000766 ± 0.000517 | 0 | 0 | 0 | 0 | 0 | 0.000685 ±  0.00048 | 0 | 0.000458 ± 0.000285 | 0.000554 ± 0.000391 | 0 | 0.000314 ± 0.000629 | 0.000226 ±  0.00027 |
| UBA2991_X | 0.006330 | 0 | 0.000215 ± 0.000431 | 0 | 0.000276 ± 0.000553 | 0.000205 ± 0.000303 | 0 | 0.001333 ± 0.000424 | 0.000544 ± 0.001087 | 0.000519 ±  0.00051 | 0.000224 ± 0.000323 | 0 | 0.001451 ± 0.001237 | 0.000424 ± 0.000559 |
| UBA2999_X | 0.000049 | 0.010634 ± 0.001304 | 0.010531 ±  0.00141 | 0.008332 ± 0.002733 | 0.007699 ±  0.00224 | 0.005604 ± 0.000967 | 0.006747 ± 0.001984 | 0.004093 ±  0.001 | 0.007551 ± 0.005737 | 0.00773  ± 0.001511 | 0.003397 ± 0.001957 | 0.010858 ± 0.002149 | 0.001103 ± 0.002205 | 0.011252 ± 0.001412 |
| UBA3495_X | 0.484612 | 0 | 0 | 0 | 0 | 0 | 0 | 0 | 0 | 0 | 0.000023 ± 0.000053 | 0 | 0 | 0.000152 ± 0.000304 |
| UBA4664 | 0.008701 | 0 | 0 | 0 | 0 | 0.000138 ± 0.000151 | 0 | 0 | 0 | 0.000038 ± 0.000084 | 0 | 0 | 0 | 0 |
| UBA4665_X | 0.006263 | 0.004112 ± 0.005209 | 0 | 0.00208  ± 0.002132 | 0.000776 ± 0.000983 | 0.000519 ± 0.000595 | 0.000244 ± 0.000453 | 0.004126 ± 0.002834 | 0.001675 ± 0.001569 | 0.002104 ± 0.001238 | 0.004304 ± 0.002886 | 0.000156 ± 0.000348 | 0.002155 ±  0.00431 | 0.001003 ± 0.001042 |
| UBA4720 | 0.024383 | 0 | 0.00135  ± 0.000953 | 0.000441 ± 0.000605 | 0.000515 ±  0.00103 | 0.00071  ±  0.00043 | 0.000111 ± 0.000248 | 0.000456 ± 0.000452 | 0 | 0.001135 ± 0.000506 | 0.000282 ± 0.000386 | 0.000287 ± 0.000434 | 0.000452 ± 0.000904 | 0 |
| UBA4722 | 0.316957 | 0 | 0 | 0 | 0.000563 ± 0.001126 | 0 | 0 | 0 | 0 | 0 | 0 | 0 | 0 | 0 |
| UBA4738_X_X_X | 0.546132 | 0 | 0 | 0.000208 ± 0.000465 | 0 | 0 | 0 | 0 | 0 | 0 | 0 | 0 | 0 | 0 |
| UBA4823_X | 0.001000 | 0 | 0 | 0 | 0.002003 ± 0.001693 | 0 | 0 | 0.001208 ± 0.001925 | 0 | 0 | 0 | 0 | 0 | 0 |
| UBA5704_X | 0.009419 | 0.000786 ± 0.000595 | 0.000752 ± 0.001505 | 0.001456 ± 0.000501 | 0.00346  ± 0.000328 | 0.000561 ± 0.000839 | 0.00025  ± 0.000559 | 0.000982 ±  0.00104 | 0.001624 ± 0.002418 | 0.000605 ±  0.00062 | 0.000166 ± 0.000371 | 0 | 0.00017  ± 0.000198 | 0.000179 ± 0.000359 |
| UBA6082 | 0.001378 | 0.004427 ± 0.002294 | 0.001342 ± 0.001672 | 0.001796 ± 0.001288 | 0.003252 ± 0.001347 | 0.005159 ± 0.001732 | 0.001917 ± 0.002712 | 0.001894 ± 0.000983 | 0.010831 ±  0.00188 | 0.005335 ± 0.000512 | 0.003843 ± 0.001023 | 0.003482 ± 0.002065 | 0.001886 ± 0.003773 | 0.005137 ± 0.001077 |
| UBA6175 | 0.316957 | 0 | 0 | 0 | 0 | 0 | 0 | 0 | 0 | 0 | 0 | 0 | 0.000276 ± 0.000551 | 0 |
| UBA6265_X | 0.316957 | 0 | 0 | 0 | 0 | 0 | 0 | 0 | 0 | 0 | 0 | 0 | 0 | 0.000178 ± 0.000355 |
| UBA695 | 0.044935 | 0.003994 ± 0.001081 | 0.004367 ± 0.002499 | 0.003565 ±  0.00055 | 0.002494 ± 0.003391 | 0.005042 ± 0.001897 | 0.004512 ± 0.001725 | 0.001925 ± 0.000669 | 0.002751 ± 0.001428 | 0.005056 ± 0.001724 | 0.006767 ± 0.004505 | 0.004598 ± 0.002112 | 0.007054 ± 0.003774 | 0.006813 ± 0.004888 |
| UBA6960_X | 0.205013 | 0 | 0 | 0 | 0.000301 ± 0.000603 | 0.00038  ± 0.000526 | 0 | 0 | 0 | 0 | 0.000268 ± 0.000373 | 0 | 0 | 0.000254 ± 0.000507 |
| UBA8199 | 0.000009 | 0 | 0 | 0 | 0 | 0 | 0 | 0 | 0 | 0.000855 ± 0.000559 | 0 | 0 | 0 | 0 |
| UBA9160_X | 0.037488 | 0.000303 ± 0.000352 | 0 | 0 | 0 | 0 | 0 | 0 | 0 | 0 | 0 | 0 | 0.000314 ± 0.000629 | 0 |
| UBA9160_X_X | 0.150299 | 0 | 0 | 0 | 0 | 0 | 0 | 0.000232 ± 0.000332 | 0 | 0 | 0.000169 ± 0.000378 | 0 | 0 | 0 |
| UBA9160_X_X_X | 0.708879 | 0 | 0 | 0 | 0 | 0.000231 ± 0.000516 | 0 | 0.000269 ± 0.000602 | 0.000129 ± 0.000258 | 0 | 0.000115 ± 0.000256 | 0 | 0 | 0 |
| UBA920 | 0.484612 | 0 | 0.000147 ± 0.000294 | 0.000078 ± 0.000175 | 0 | 0 | 0 | 0 | 0 | 0 | 0 | 0 | 0 | 0 |
| UBA964 | 0.170643 | 0.002298 ± 0.001412 | 0.000861 ± 0.001722 | 0.000551 ± 0.001122 | 0.001507 ± 0.001779 | 0.00228  ± 0.001483 | 0.001843 ± 0.001905 | 0.001049 ± 0.000717 | 0.001627 ± 0.002535 | 0.002663 ± 0.001745 | 0.001254 ± 0.000849 | 0.001299 ± 0.001363 | 0 | 0.000523 ± 0.000688 |
| UBA9968_X | 0.546132 | 0 | 0 | 0 | 0 | 0 | 0 | 0.00007  ± 0.000157 | 0 | 0 | 0 | 0 | 0 | 0 |
| UBA9983_A_X_X | 0.598062 | 0 | 0 | 0 | 0 | 0 | 0 | 0.000056 ± 0.000125 | 0.000115 ±  0.00023 | 0 | 0.000047 ± 0.000106 | 0 | 0 | 0 |
| UKL13-2 | 0.070844 | 0 | 0 | 0 | 0.000903 ± 0.001807 | 0.000595 ± 0.000832 | 0.001028 ±  0.00083 | 0 | 0 | 0.000094 ± 0.000209 | 0.000551 ± 0.001019 | 0.000287 ± 0.000641 | 0 | 0.000401 ± 0.000518 |
| UKL13-2_X | 0.028206 | 0.005101 ± 0.001225 | 0.00546  ± 0.000805 | 0.003792 ± 0.001883 | 0.007725 ± 0.001139 | 0.005176 ±  0.00074 | 0.00278  ± 0.002613 | 0.003536 ± 0.000777 | 0.00145  ± 0.002899 | 0.004888 ± 0.001559 | 0.003731 ± 0.001472 | 0.003513 ± 0.002228 | 0.005241 ± 0.002347 | 0.004884 ± 0.001668 |
| UKL13-3 | 0.610162 | 0 | 0 | 0 | 0 | 0.000105 ± 0.000235 | 0 | 0 | 0 | 0.000076 ± 0.00017 | 0 | 0 | 0 | 0.000057 ± 0.000113 |
| URHD0088 | 0.007029 | 0.000607 ± 0.001214 | 0 | 0 | 0 | 0.001351 ± 0.000847 | 0.000516 ± 0.001155 | 0 | 0 | 0.000512 ± 0.000711 | 0 | 0 | 0 | 0 |
| UTPRO1_X | 0.692967 | 0 | 0 | 0 | 0 | 0 | 0 | 0.00011  ± 0.000246 | 0 | 0.000077 ± 0.000172 | 0.000048 ± 0.000108 | 0 | 0 | 0 |
| V1-33_X | 0.251780 | 0 | 0.00028  ± 0.000561 | 0.000171 ± 0.000383 | 0 | 0.000116 ± 0.000259 | 0 | 0.000375 ± 0.000411 | 0 | 0.000475 ± 0.000738 | 0.00048  ± 0.000505 | 0.000069 ± 0.000154 | 0 | 0.000228 ± 0.000456 |
| VAYN01 | 0.037488 | 0.000365 ± 0.000731 | 0.000461 ± 0.000538 | 0 | 0 | 0 | 0 | 0 | 0 | 0 | 0 | 0 | 0 | 0 |
| VAZQ01 | 0.000020 | 0.001475 ± 0.000726 | 0.001191 ± 0.000961 | 0.001882 ±  0.00056 | 0.000314 ± 0.000628 | 0.00804  ± 0.002199 | 0.006302 ±  0.00159 | 0.001365 ± 0.000689 | 0.000496 ± 0.000992 | 0.002804 ± 0.001117 | 0.004111 ± 0.001818 | 0.002684 ± 0.001878 | 0 | 0.001937 ± 0.001343 |
| VBAS01 | 0.123693 | 0 | 0.000544 ±  0.00065 | 0.000322 ±  0.00072 | 0.000452 ± 0.000903 | 0.000551 ± 0.000768 | 0.000123 ± 0.000274 | 0.000908 ± 0.000672 | 0 | 0.00053  ± 0.000749 | 0.00043  ± 0.000504 | 0.000107 ±  0.00024 | 0 | 0 |
| VBCG01 | 0.000153 | 0.003244 ± 0.000553 | 0.003586 ± 0.001091 | 0.004163 ± 0.003264 | 0.001425 ± 0.002204 | 0.003525 ± 0.000337 | 0.00233  ± 0.000581 | 0.001282 ± 0.001226 | 0.000603 ± 0.001206 | 0.004631 ± 0.000814 | 0.003385 ± 0.000248 | 0.001049 ± 0.001455 | 0.000239 ± 0.000478 | 0.001762 ±  0.00019 |
| Verrucomicrobiaceae_X | 0.124815 | 0 | 0.00062  ± 0.001239 | 0.000785 ± 0.001103 | 0.000529 ± 0.001057 | 0 | 0.00146  ± 0.001069 | 0.000257 ± 0.000353 | 0.000129 ± 0.000258 | 0.000303 ± 0.000678 | 0.000982 ± 0.001053 | 0 | 0 | 0.00144  ± 0.002117 |
| Verrucomicrobiae_X_X_X | 0.618381 | 0 | 0 | 0 | 0 | 0.000193 ± 0.000431 | 0 | 0.00008  ±  0.00018 | 0 | 0 | 0 | 0 | 0 | 0 |
| Verrucomicrobiales_X_X | 0.653159 | 0.000295 ± 0.000591 | 0 | 0.00075  ± 0.000777 | 0.000537 ± 0.000621 | 0.000173 ± 0.000238 | 0.000336 ± 0.000526 | 0.000363 ± 0.000811 | 0.000459 ± 0.000919 | 0.000902 ± 0.000998 | 0.000245 ± 0.000549 | 0.000123 ± 0.000274 | 0 | 0.000296 ± 0.000592 |
| Verrucomicrobium | 0.004918 | 0 | 0.001365 ± 0.001739 | 0.000653 ± 0.001276 | 0 | 0 | 0.000087 ± 0.000195 | 0 | 0.000086 ± 0.000172 | 0.000358 ± 0.000266 | 0.00052  ± 0.000323 | 0.002619 ± 0.003004 | 0 | 0.000816 ± 0.001304 |
| Vicinamibacterales_X_X | 0.020005 | 0.054499 ± 0.005981 | 0.039201 ± 0.005382 | 0.038947 ± 0.014797 | 0.056446 ± 0.022864 | 0.045843 ± 0.009136 | 0.054602 ± 0.008915 | 0.036558 ± 0.004654 | 0.055658 ± 0.007202 | 0.055313 ± 0.016554 | 0.037539 ± 0.008793 | 0.046824 ± 0.010185 | 0.043675 ± 0.013355 | 0.060904 ± 0.011961 |
| Viridilinea | 0.370923 | 0.00027  ± 0.000539 | 0 | 0 | 0 | 0 | 0 | 0 | 0 | 0 | 0 | 0 | 0.000294 ± 0.000588 | 0 |
| Vitiosangium | 0.005931 | 0.006137 ± 0.002493 | 0.011336 ± 0.002995 | 0.008939 ± 0.003439 | 0.01284  ± 0.002228 | 0.004767 ±  0.00118 | 0.006944 ± 0.003225 | 0.004575 ±  0.00224 | 0.006315 ± 0.004787 | 0.003888 ± 0.000986 | 0.004741 ± 0.001912 | 0.008173 ± 0.002553 | 0.006334 ±  0.00153 | 0.006584 ± 0.001576 |
| VXMT01 | 0.041352 | 0.000246 ± 0.000492 | 0.000801 ± 0.000561 | 0.000092 ± 0.000205 | 0 | 0.000457 ± 0.000268 | 0.000295 ± 0.000424 | 0.000357 ± 0.000389 | 0 | 0.000454 ± 0.000287 | 0.000072 ± 0.000162 | 0.000092 ± 0.000206 | 0 | 0.000364 ± 0.000446 |
| WHTF01 | 0.000080 | 0.00388  ± 0.001697 | 0.00121  ± 0.001389 | 0.000628 ± 0.000884 | 0.003135 ± 0.001176 | 0.002218 ± 0.000869 | 0.001544 ± 0.000986 | 0.005745 ± 0.000712 | 0.008547 ± 0.004741 | 0.004441 ± 0.001654 | 0.001696 ± 0.000884 | 0.002444 ± 0.000954 | 0.001391 ± 0.001083 | 0.00408  ±  0.001553 |
| WHTK01 | 0.430389 | 0.001385 ± 0.000987 | 0.000485 ± 0.000971 | 0.000747 ± 0.001062 | 0.001632 ± 0.001547 | 0.00188  ±  0.001077 | 0.000831 ±  0.00126 | 0.002302 ± 0.001686 | 0.001479 ± 0.002258 | 0.000859 ± 0.000928 | 0.000841 ± 0.000804 | 0.000319 ± 0.000714 | 0.000663 ± 0.000861 | 0.001018 ± 0.001115 |
| WHTO01 | 0.113607 | 0 | 0 | 0 | 0.000327 ± 0.000653 | 0 | 0 | 0.000264 ± 0.000391 | 0 | 0 | 0 | 0 | 0 | 0 |
| WQYP01 | 0.618381 | 0 | 0 | 0 | 0 | 0.000059 ± 0.000133 | 0 | 0 | 0 | 0.000072 ±  0.00016 | 0 | 0 | 0 | 0 |
| WS-7 | 0.000181 | 0.00007 ± 0.000139 | 0.000514 ± 0.000345 | 0.000108 ± 0.000241 | 0.000452 ± 0.000904 | 0.001311 ± 0.000913 | 0.000253 ± 0.000347 | 0.001661 ± 0.000917 | 0.00086  ±  0.000994 | 0.002389 ± 0.000803 | 0.001421 ± 0.000215 | 0 | 0 | 0.000752 ± 0.000564 |
| WYBJ01 | 0.049135 | 0.000328 ± 0.000656 | 0.000294 ± 0.000587 | 0 | 0.001097 ± 0.001547 | 0.000999 ± 0.001461 | 0.000853 ± 0.000839 | 0.000421 ± 0.000576 | 0.000258 ± 0.000517 | 0.001319 ± 0.000341 | 0.000681 ± 0.000461 | 0 | 0 | 0.000596 ± 0.000698 |
| WYBL01 | 0.019423 | 0.005681 ± 0.000903 | 0.003929 ± 0.002741 | 0.004436 ±  0.00195 | 0.004683 ± 0.000616 | 0.005821 ± 0.001744 | 0.005958 ± 0.001884 | 0.006509 ± 0.002832 | 0.007862 ± 0.006135 | 0.007491 ± 0.002489 | 0.005504 ± 0.001558 | 0.001387 ± 0.001273 | 0.003155 ± 0.002258 | 0.006769 ± 0.001965 |
| WYBW01 | 0.598062 | 0 | 0 | 0 | 0 | 0.000225 ± 0.000503 | 0 | 0 | 0.000627 ± 0.001253 | 0 | 0 | 0.000139 ± 0.000311 | 0 | 0 |
| Xanthobacteraceae_X | 0.110222 | 0.000755 ± 0.000873 | 0.000697 ± 0.000861 | 0 | 0.000596 ± 0.000689 | 0 | 0.000357 ± 0.000345 | 0.000051 ± 0.000114 | 0 | 0.000269 ± 0.000378 | 0 | 0 | 0.00045  ± 0.000901 | 0 |
| Xanthomonadaceae_X | 0.030029 | 0.009213 ±  0.00171 | 0.011877 ± 0.003056 | 0.009098 ± 0.001615 | 0.006759 ± 0.003644 | 0.004619 ± 0.001659 | 0.008137 ± 0.003355 | 0.006271 ± 0.001561 | 0.006334 ± 0.003591 | 0.00557  ± 0.002899 | 0.006495 ± 0.002699 | 0.007868 ± 0.003056 | 0.013074 ± 0.004975 | 0.007385 ± 0.003255 |
| Xanthomonadales_X_X | 0.072547 | 0.000794 ±  0.00068 | 0.001712 ± 0.001335 | 0.001856 ± 0.002769 | 0.000138 ± 0.000276 | 0.000542 ± 0.000804 | 0.000117 ± 0.000261 | 0.000541 ± 0.000762 | 0 | 0 | 0.000385 ± 0.000694 | 0 | 0.000366 ± 0.000732 | 0.000567 ± 0.000511 |
| Xanthomonas | 0.037830 | 0 | 0 | 0 | 0 | 0 | 0 | 0 | 0 | 0.001246 ± 0.001809 | 0 | 0 | 0 | 0 |
| Xylophilus | 0.000375 | 0.009302 ± 0.004356 | 0.009962 ±  0.00249 | 0.009093 ± 0.002632 | 0.00884  ± 0.001045 | 0.006977 ± 0.002419 | 0.008979 ± 0.003517 | 0.002207 ± 0.001355 | 0.004103 ±  0.00489 | 0.002972 ± 0.000902 | 0.004048 ± 0.000918 | 0.011256 ± 0.003678 | 0.010453 ± 0.004213 | 0.007645 ± 0.001389 |
| Z2-YC6860 | 0.026576 | 0 | 0 | 0 | 0.002065 ±  0.00413 | 0.000621 ± 0.001056 | 0.000358 ±  0.0008 | 0.00022  ± 0.000492 | 0 | 0.002022 ± 0.001404 | 0.000189 ± 0.000422 | 0 | 0 | 0 |
| Zavarzinella | 0.015110 | 0 | 0 | 0.001631 ± 0.002247 | 0 | 0.000162 ± 0.000362 | 0.002274 ± 0.002019 | 0.000355 ± 0.000346 | 0 | 0 | 0.000358 ± 0.000359 | 0.000606 ± 0.001356 | 0 | 0 |
| ZC4RG30 | 0.618381 | 0 | 0 | 0 | 0 | 0.000204 ± 0.000457 | 0 | 0 | 0 | 0 | 0.000261 ± 0.000584 | 0 | 0 | 0 |

**Table S4**: p-values from pairwise adonis between all farm’s samples.

|  | F02 | F03 | F04 | F05 | F07 | F08 | F10 | F11 | F12 | F13 | F14 | F15 |
| --- | --- | --- | --- | --- | --- | --- | --- | --- | --- | --- | --- | --- |
| F01 | 0.039 | 0.006 | 0.033 | 0.012 | 0.01 | 0.008 | 0.022 | 0.01 | 0.007 | 0.01 | 0.034 | 0.026 |
| F02 |  | 0.005 | 0.021 | 0.008 | 0.008 | 0.007 | 0.028 | 0.007 | 0.012 | 0.008 | 0.03 | 0.037 |
| F03 |  |  | 0.012 | 0.01 | 0.008 | 0.003 | 0.007 | 0.01 | 0.007 | 0.011 | 0.009 | 0.005 |
| F04 |  |  |  | 0.006 | 0.009 | 0.012 | 0.033 | 0.011 | 0.008 | 0.01 | 0.024 | 0.027 |
| F05 |  |  |  |  | 0.008 | 0.006 | 0.007 | 0.006 | 0.01 | 0.003 | 0.009 | 0.006 |
| F07 |  |  |  |  |  | 0.007 | 0.008 | 0.009 | 0.009 | 0.011 | 0.009 | 0.008 |
| F08 |  |  |  |  |  |  | 0.011 | 0.007 | 0.006 | 0.009 | 0.006 | 0.01 |
| F10 |  |  |  |  |  |  |  | 0.009 | 0.012 | 0.01 | 0.033 | 0.035 |
| F11 |  |  |  |  |  |  |  |  | 0.009 | 0.006 | 0.008 | 0.012 |
| F12 |  |  |  |  |  |  |  |  |  | 0.006 | 0.009 | 0.006 |
| F13 |  |  |  |  |  |  |  |  |  |  | 0.011 | 0.008 |
| F14 |  |  |  |  |  |  |  |  |  |  |  | 0.032 |

**Table S5:** List of media used for culture dependent study.

| Media | Targeted organisms |
| --- | --- |
| R_2_A | Slow growing bacteria |
| Soil extract agar | Common soil bacteria |
| Actinomycetes isolation agar | Actinomycetes isolation |
| Azotobacter mannitol agar | Azotobacter isolation |
| Tryptic soy agar | Fastidious growing organisms |
| Nutrient agar | General bacteria |
| Pseudomonas agar | Pseudomonas isolation |
| Yeast mannitol agar w/Congo red | Rhizobium isolation |

**Supplementary method**

**Initial quality check using DADA2 pipeline**

All the initial necessary quality parameter were checked for both approaches’ sequences. The first step of DADA2 pipeline was to quality filter the data. This step was necessary to remove the low-quality sequences or to trim the poor-quality portion from the sequences**.** The sequence quality starts degrading towards the righthand side or 3’ end in both the approaches.

For CI approach run (run-1), average quality score of forward reads show in the range of 40 to 30. While for reverse reads it observed in the range of 40 to 25 at 3’ end. This is commonly observed in all the NGS data and hence, should be adjusted accordingly. Also, the reverse/R2 reads data were of comparatively poor quality than forward/R1 reads. So, it was decided to trim 2 and 11 bases from the right-end for R1 and R2 reads, respectively. The trimming parameters were decided upon considering that sufficient overlap of >20 bases should remain after trimming to allow the merging of paired reads. While the overall data quality was comparatively better, reads with expected error >3 were filtered from both files.

For CD approach run (run-2), average quality score of forward reads show in the range of 40 to 30. While for reverse reads it observed in the range of 40 to 35 at 3’ end. So, it was decided to trim 2 and 6 bases from the right-end for R1 and R2 reads, respectively. Further reads with expected error >2 were filtered from both files.

Additionally, in both approaches, sequences with any ambiguous base (N) or sequences from PhiX (control library used during sequencing) were also filtered out. Further 17 and 21 bases were trimmed from the left side (5’ end) of R1 and R2 sequences, respectively to remove the primer sequences.

The next step was to infer the error rates and use that to denoise the data and infer sequence variants. This was performed separately for forward and reverse reads using their respective error rates.

The next step was merging both denoised forward and reverse data to obtain full denoised sequences. Merging was performed by aligning the denoised forward sequences with reverse-complement of the corresponding denoised reverse sequences. The merging was based on the successful alignment of at least 12 bases from the 3’ end without any mismatches. The merged denoised data was then used to prepare the Amplicon Sequence Variant (ASV) table. Furthermore, the length of targeted regions was expected at approximately 402 and 428 base pairs. Therefore, only the variants with merged lengths in the range of 400 to 430 were kept in both approaches (Figure S13, Figure S14).


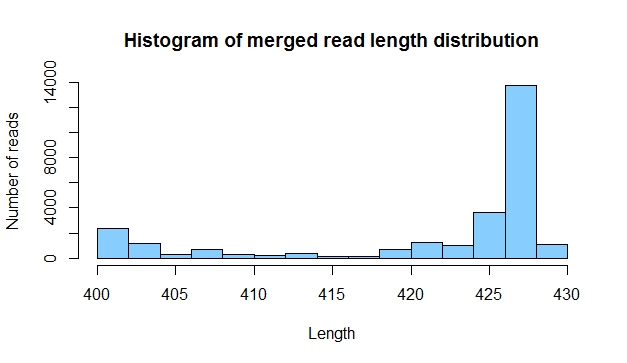


Figure S13: Distribution of merged read length from CI approach


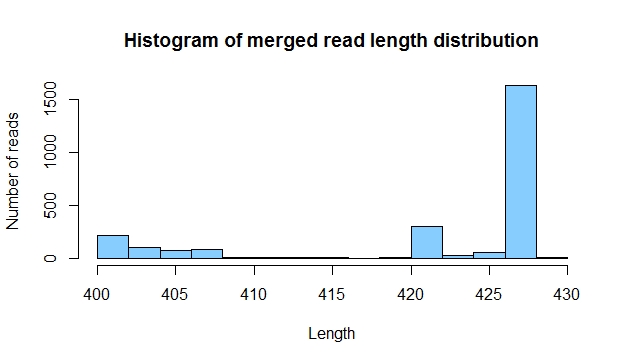


Figure S14: Distribution of merged read length from CD approach.

The next step was to remove the chimeric sequences or bimeric sequences from the dataset. Around 95.65% of sequences remained after chimera removal, which indicates high-quality data. A check was also made to evaluate the proportion of sequences remaining after chimera removal. Additionally, the number of reads at each step of the pipeline was tracked to identify any issues with the pipeline parameters.

**For metagenomic study through CI approach**

Functions within DADA2 were used to check the quality, trim, and quality filter data; infer error rate; denoise data; merge pairs; remove chimera and lastly to generate amplicon sequence variants (ASV) table. Table S6 summarizes the read counts across each step of the DADA2 pipeline. The ASVs were assigned taxonomy using GTDB database.

Table S6: Details of reads at each step during DADA2 processing.

| Sample | Input reads x2 | Quality filtered reads x2 | Denoised forward reads | Denoised reverse reads | Merged reads | Non-chimeric sequences |
| --- | --- | --- | --- | --- | --- | --- |
| F01-1 | 83845 | 49327 | 46353 | 44424 | 31604 | 30201 |
| F01-2 | 46103 | 27653 | 25706 | 23771 | 16660 | 15803 |
| F01-3 | 45673 | 27776 | 25608 | 24004 | 16684 | 15867 |
| F01-4 | 51978 | 31757 | 29556 | 28186 | 19976 | 19147 |
| F01-5 | 33867 | 18188 | 16451 | 14581 | 9380 | 8661 |
| F02-1 | 54969 | 32996 | 30822 | 28810 | 20297 | 19106 |
| F02-2 | 50990 | 30920 | 28737 | 26949 | 18720 | 17684 |
| F02-3 | 41485 | 22237 | 20348 | 18526 | 12401 | 11795 |
| F02-4 | 43235 | 9624 | 8411 | 6372 | 3805 | 3308 |
| F02-5 | 69989 | 43862 | 41277 | 38991 | 28059 | 26833 |
| F03-1 | 78552 | 39170 | 36404 | 33155 | 21926 | 20828 |
| F03-2 | 67229 | 36193 | 34139 | 31699 | 22839 | 21976 |
| F03-3 | 74255 | 36419 | 34134 | 31499 | 22247 | 21294 |
| F03-4 | 66761 | 35605 | 33282 | 31002 | 21067 | 20317 |
| F03-5 | 66184 | 41505 | 39083 | 37311 | 28154 | 26989 |
| F04-1 | 64154 | 26819 | 24563 | 21266 | 13415 | 12771 |
| F04-2 | 44761 | 23116 | 21101 | 18307 | 11506 | 10723 |
| F04-3 | 77227 | 30097 | 27737 | 23766 | 15711 | 14726 |
| F04-4 | 27920 | 12566 | 10908 | 8238 | 4741 | 4305 |
| F04-5 | 71496 | 39548 | 36902 | 34480 | 23581 | 22277 |
| F05-1 | 76069 | 50382 | 47369 | 46011 | 34105 | 32701 |
| F05-2 | 73171 | 50430 | 47840 | 46548 | 35943 | 34204 |
| F05-3 | 66316 | 44399 | 41682 | 40754 | 30089 | 28800 |
| F05-4 | 68102 | 45932 | 43268 | 41914 | 31644 | 30222 |
| F05-5 | 55342 | 36206 | 33813 | 32648 | 23202 | 21982 |
| F07-1 | 70746 | 41871 | 39375 | 37446 | 27424 | 26293 |
| F07-2 | 94105 | 56475 | 53576 | 51371 | 37689 | 36127 |
| F07-3 | 88516 | 50837 | 48285 | 45914 | 34258 | 32775 |
| F07-4 | 64739 | 34490 | 32096 | 29184 | 20256 | 19158 |
| F07-5 | 52643 | 35042 | 32630 | 31307 | 22907 | 21907 |
| F08-1 | 253757 | 126021 | 120735 | 117518 | 92829 | 88419 |
| F08-2 | 71119 | 44858 | 41683 | 39866 | 28269 | 26811 |
| F08-3 | 54438 | 34049 | 31598 | 29972 | 20923 | 19769 |
| F08-4 | 56439 | 32529 | 30040 | 27844 | 19107 | 18108 |
| F08-5 | 108166 | 73519 | 69691 | 68026 | 53005 | 50793 |
| F10-1 | 68340 | 24042 | 21851 | 18078 | 10504 | 9942 |
| F10-2 | 70677 | 26975 | 24769 | 20949 | 13324 | 12546 |
| F10-3 | 60873 | 22462 | 20309 | 16691 | 9765 | 9143 |
| F10-4 | 69815 | 24127 | 21815 | 17871 | 10835 | 9999 |
| F10-5 | 51952 | 34167 | 31352 | 29977 | 20183 | 18973 |
| F11-1 | 86628 | 60763 | 56928 | 55769 | 41323 | 39291 |
| F11-2 | 75116 | 53177 | 49651 | 48596 | 35401 | 33671 |
| F11-3 | 69063 | 49714 | 46439 | 45167 | 33426 | 31577 |
| F11-4 | 68690 | 49984 | 46795 | 45920 | 35571 | 34084 |
| F11-5 | 50148 | 33370 | 30811 | 29237 | 20127 | 19033 |
| F12-1 | 74636 | 52767 | 49806 | 48386 | 36590 | 35038 |
| F12-2 | 66250 | 45818 | 43054 | 42156 | 31713 | 30226 |
| F12-3 | 75163 | 53154 | 50015 | 49137 | 37425 | 35842 |
| F12-4 | 62607 | 43529 | 40864 | 39715 | 30010 | 28798 |
| F12-5 | 47032 | 30490 | 28131 | 26906 | 18511 | 17756 |
| F13-1 | 84638 | 32810 | 30687 | 27460 | 18407 | 17370 |
| F13-2 | 113388 | 44082 | 41586 | 38063 | 26508 | 25391 |
| F13-3 | 57556 | 23887 | 22156 | 18987 | 13147 | 12392 |
| F13-4 | 69517 | 28160 | 26021 | 22921 | 14566 | 13863 |
| F13-5 | 95853 | 47193 | 44276 | 41251 | 28866 | 27545 |
| F14-1 | 54377 | 19545 | 17882 | 14488 | 8991 | 8490 |
| F14-2 | 51699 | 22308 | 20577 | 16988 | 11493 | 10645 |
| F14-3 | 58213 | 24821 | 22835 | 19973 | 13380 | 12866 |
| F14-4 | 72965 | 26633 | 24659 | 21398 | 14130 | 13273 |
| F14-5 | 81915 | 43262 | 41010 | 38472 | 29230 | 28333 |
| F15-1 | 79675 | 46506 | 43478 | 41835 | 30758 | 29262 |
| F15-2 | 85488 | 51092 | 48042 | 45625 | 32864 | 31027 |
| F15-3 | 88837 | 53646 | 50526 | 48482 | 35996 | 34474 |
| F15-4 | 389621 | 237667 | 231498 | 229547 | 193558 | 184941 |
| F15-5 | 73693 | 37342 | 35230 | 32594 | 24113 | 23051 |

The ASV table, taxonomy information and sample metadata were merged in a single phyloseq object. This phyloseq object contains data from 65 samples and 17,719 ASVs. Since ASVs differ by a single nucleotide, there is a chance that some of these ASVs arise because of errors in sequencing or other PCR artifacts. To reduce such biases, all ASVs with read support <20 were removed. Left behind 8,042 ASVs from 65 samples.

Further, all the samples had a different number of reads. Therefore, to eliminate biases due to less amount of data, the samples with reads < 9,600 and more than > 1,70,000 were removed. This leaving behind 8,042 ASVs from 59 samples.

This final phyloseq object was used for all subsequent steps like for alpha and beta diversity, physico-chemical parameter association, statistical comparison, core microbiome etc. Additionally, a phyloseq object with normalized abundance was also prepared to be used for some of the analysis steps. Normalization was done by converting abundances into fractions across each sample. The important sanity check here is to check the sufficient amount of data for analysis. This is done by plotting a rarefaction plot. The rarefaction plot of 59 samples showed asymptotes for all the samples highlighting the sufficient data for analysis.

**For comparative analysis of CI and CD approach**

The data generated from sequencing run of the CD approach was taken further for data analysis along with data from the CI counterpart samples. Since the DADA2 pipeline recommends processing the data from different runs separately and joining the tables at later stage, CI data was considered as data from run1, and CD data was considered as data from run2 for processing. Table S7 summarizes the read counts during each step of DADA2 pipeline processing. DADA2 inferred a total of 18,765 ASVs which were assigned taxonomy using the GTDB database.

Table S7: Summary statistics of reads through each step of analysis pipeline.

| Sample | Input reads x2 | Quality filtered reads x2 | Denoised forward reads | Denoised reverse reads | Merged reads | Non-chimeric sequences |
| --- | --- | --- | --- | --- | --- | --- |
| CI approach samples | | | | | | |
| F01-1 | 83845 | 49327 | 46353 | 44424 | 31604 | 30201 |
| F01-2 | 46103 | 27653 | 25706 | 23771 | 16660 | 15803 |
| F01-3 | 45673 | 27776 | 25608 | 24004 | 16684 | 15867 |
| F01-4 | 51978 | 31757 | 29556 | 28186 | 19976 | 19147 |
| F01-5 | 33867 | 18188 | 16451 | 14581 | 9380 | 8661 |
| F02-1 | 54969 | 32996 | 30822 | 28810 | 20297 | 19106 |
| F02-2 | 50990 | 30920 | 28737 | 26949 | 18720 | 17684 |
| F02-3 | 41485 | 22237 | 20348 | 18526 | 12401 | 11795 |
| F02-4 | 43235 | 9624 | 8411 | 6372 | 3805 | 3308 |
| F02-5 | 69989 | 43862 | 41277 | 38991 | 28059 | 26833 |
| F03-1 | 78552 | 39170 | 36404 | 33155 | 21926 | 20828 |
| F03-2 | 67229 | 36193 | 34139 | 31699 | 22839 | 21976 |
| F03-3 | 74255 | 36419 | 34134 | 31499 | 22247 | 21294 |
| F03-4 | 66761 | 35605 | 33282 | 31002 | 21067 | 20317 |
| F03-5 | 66184 | 41505 | 39083 | 37311 | 28154 | 26989 |
| F04-1 | 64154 | 26819 | 24563 | 21266 | 13415 | 12771 |
| F04-2 | 44761 | 23116 | 21101 | 18307 | 11506 | 10723 |
| F04-3 | 77227 | 30097 | 27737 | 23766 | 15711 | 14726 |
| F04-4 | 27920 | 12566 | 10908 | 8238 | 4741 | 4305 |
| F04-5 | 71496 | 39548 | 36902 | 34480 | 23581 | 22277 |
| F05-1 | 76069 | 50382 | 47369 | 46011 | 34105 | 32701 |
| F05-2 | 73171 | 50430 | 47840 | 46548 | 35943 | 34204 |
| F05-3 | 66316 | 44399 | 41682 | 40754 | 30089 | 28800 |
| F05-4 | 68102 | 45932 | 43268 | 41914 | 31644 | 30222 |
| F05-5 | 55342 | 36206 | 33813 | 32648 | 23202 | 21982 |
| F07-1 | 70746 | 41871 | 39375 | 37446 | 27424 | 26293 |
| F07-2 | 94105 | 56475 | 53576 | 51371 | 37689 | 36127 |
| F07-3 | 88516 | 50837 | 48285 | 45914 | 34258 | 32775 |
| F07-4 | 64739 | 34490 | 32096 | 29184 | 20256 | 19158 |
| F07-5 | 52643 | 35042 | 32630 | 31307 | 22907 | 21907 |
| F08-1 | 253757 | 126021 | 120735 | 117518 | 92829 | 88419 |
| F08-2 | 71119 | 44858 | 41683 | 39866 | 28269 | 26811 |
| F08-3 | 54438 | 34049 | 31598 | 29972 | 20923 | 19769 |
| F08-4 | 56439 | 32529 | 30040 | 27844 | 19107 | 18108 |
| F08-5 | 108166 | 73519 | 69691 | 68026 | 53005 | 50793 |
| F10-1 | 68340 | 24042 | 21851 | 18078 | 10504 | 9942 |
| F10-2 | 70677 | 26975 | 24769 | 20949 | 13324 | 12546 |
| F10-3 | 60873 | 22462 | 20309 | 16691 | 9765 | 9143 |
| F10-4 | 69815 | 24127 | 21815 | 17871 | 10835 | 9999 |
| F10-5 | 51952 | 34167 | 31352 | 29977 | 20183 | 18973 |
| F11-1 | 86628 | 60763 | 56928 | 55769 | 41323 | 39291 |
| F11-2 | 75116 | 53177 | 49651 | 48596 | 35401 | 33671 |
| F11-3 | 69063 | 49714 | 46439 | 45167 | 33426 | 31577 |
| F11-4 | 68690 | 49984 | 46795 | 45920 | 35571 | 34084 |
| F11-5 | 50148 | 33370 | 30811 | 29237 | 20127 | 19033 |
| F12-1 | 74636 | 52767 | 49806 | 48386 | 36590 | 35038 |
| F12-2 | 66250 | 45818 | 43054 | 42156 | 31713 | 30226 |
| F12-3 | 75163 | 53154 | 50015 | 49137 | 37425 | 35842 |
| F12-4 | 62607 | 43529 | 40864 | 39715 | 30010 | 28798 |
| F12-5 | 47032 | 30490 | 28131 | 26906 | 18511 | 17756 |
| F13-1 | 84638 | 32810 | 30687 | 27460 | 18407 | 17370 |
| F13-2 | 113388 | 44082 | 41586 | 38063 | 26508 | 25391 |
| F13-3 | 57556 | 23887 | 22156 | 18987 | 13147 | 12392 |
| F13-4 | 69517 | 28160 | 26021 | 22921 | 14566 | 13863 |
| F13-5 | 95853 | 47193 | 44276 | 41251 | 28866 | 27545 |
| F14-1 | 54377 | 19545 | 17882 | 14488 | 8991 | 8490 |
| F14-2 | 51699 | 22308 | 20577 | 16988 | 11493 | 10645 |
| F14-3 | 58213 | 24821 | 22835 | 19973 | 13380 | 12866 |
| F14-4 | 72965 | 26633 | 24659 | 21398 | 14130 | 13273 |
| F14-5 | 81915 | 43262 | 41010 | 38472 | 29230 | 28333 |
| F15-1 | 79675 | 46506 | 43478 | 41835 | 30758 | 29262 |
| F15-2 | 85488 | 51092 | 48042 | 45625 | 32864 | 31027 |
| F15-3 | 88837 | 53646 | 50526 | 48482 | 35996 | 34474 |
| F15-4 | 389621 | 237667 | 231498 | 229547 | 193558 | 184941 |
| F15-5 | 73693 | 37342 | 35230 | 32594 | 24113 | 23051 |
| CD approach samples | | | | | | |
| F01 | 150210 | 129395 | 129063 | 129147 | 128499 | 30211 |
| F02 | 133157 | 113844 | 113682 | 113666 | 113352 | 15812 |
| F03 | 161419 | 138531 | 138298 | 138268 | 137728 | 15867 |
| F04 | 130475 | 111976 | 111695 | 111727 | 111180 | 19147 |
| F05 | 142911 | 120562 | 120280 | 120289 | 119543 | 8661 |
| F07 | 123571 | 105829 | 105616 | 105599 | 105160 | 19105 |
| F08 | 135239 | 118234 | 118028 | 118019 | 117410 | 17681 |
| F10 | 89198 | 76895 | 76625 | 76701 | 76173 | 11795 |
| F11 | 95747 | 83070 | 82798 | 82847 | 82343 | 3308 |
| F12 | 92769 | 80823 | 80595 | 80660 | 80201 | 26833 |
| F13 | 98910 | 85477 | 85263 | 85288 | 84755 | 20828 |
| F14 | 72661 | 63528 | 63355 | 63348 | 62971 | 21964 |
| F15 | 93561 | 80138 | 79975 | 79947 | 79562 | 21294 |

The ASV table, taxonomy information, and sample metadata were merged in a single phyloseq object. This phyloseq object contained data from 78 samples and 18,765 ASVs. Furthermore, since all the samples had different sequencing depths and each ASV are supported by a different number of reads, a check was made to evaluate this information for filtering. Therefore, to eliminate biases due to less amount of data, the samples with reads < 9,600 were removed. This leaving behind 73 samples.

All the ASVs with total counts <30 were removed from the analysis, and the Phyloseq object was saved. This was done to remove the possible false-positive ASVs, as most ASVs were observed in only one sample with significantly less read support. After this pre-processing step, the number of ASVs reduced to 6,970. These 6,970 ASVs from 73 samples were processed downstream for all further comparative analysis as given in R script.
